# Single-cell RNA-seq reveals the immune escape and drug resistance mechanisms of mantle cell lymphoma

**DOI:** 10.20892/j.issn.2095-3941.2020.0073

**Published:** 2020-08-15

**Authors:** Liang Wang, Steven Mo, Xin Li, Yingzhi He, Jing Yang

**Affiliations:** ^1^Department of Hematology, Beijing Tongren Hospital, Capital Medical University, Beijing 100730, China; ^2^Beijing Advanced Innovation Center for Big Data-Based Precision Medicine, Beihang University and Capital Medical University, Beijing Tongren Hospital, Beijing 100730, China; ^3^Nanning Life-Ontology Biotechnology Co., Ltd., Nanning 530000, China; ^4^Department of Hematology, Zhujiang Hospital of Southern Medical University, Guangzhou 510280, China

**Keywords:** Cell heterogeneity, immune escape, mantle cell lymphoma, multidrug resistance, scRNA-seq

## Abstract

**Objective:** Mantle cell lymphoma (MCL) is a rare subtype of non-Hodgkin lymphoma (NHL) with high heterogeneity and a high recurrence rate. How heterogenous cell populations contribute to relapse remains to be elucidated.

**Methods:** We performed single cell RNA sequencing (scRNA-seq) on approximately 4,000 bone marrow cells sampled from one patient with multidrug resistant MCL. We identified 10 subpopulations comprising 4 malignant B cell subtypes, 3 T cell subtypes, 2 dendritic cell subtypes and 1 natural killer (NK) cell subtype. Subsequently, we identified cell markers, including a series of genes associated with immune escape and drug resistance. In addition, we explored the roles of these genes in the mechanism of immune escape and drug resistance, and we verified the expression imbalance and clinical prognostic potential by using GEO datasets including 211 MCL samples.

**Results:** The major immune escape mechanisms of MCL included anti-perforin activity, decreased immunogenicity and direct inhibition of apoptosis and cell killing, as mediated by type I and II B cells. The drug resistance mechanisms of different cell clusters included drug metabolism, DNA damage repair, apoptosis and survival promotion. Type III B cells closely communicate with other cells. The key genes involved in the resistance mechanisms showed dysregulated expression and may have significant clinical prognostic value.

**Conclusion:** This study investigated potential immune escape and drug resistance mechanisms in MCL. The results may guide individualized treatment and promote the development of therapeutic drugs.

## Introduction

Mantle cell lymphoma (MCL) is a relatively rare subtype of non-Hodgkin lymphoma (NHL) characterized by chromosomal translocation (11;14) resulting in constitutive overexpression of cyclin D1. Most patients with MCLs must be treated quickly, and the treatment dose intensity should be individualized according to patient fitness status. For example, younger patients are usually treated with high dose cytarabine combined with autologous stem cell transplantation for consolidation therapy and rituximab for maintenance therapy. Moreover, conventional chemotherapy has a high remission rate in previously untreated patients with MCL, and most patients eventually relapse and have a median overall survival of 5 to 7 years^1,2^. Bruton’s tyrosine kinase (BTK) is a key component of the B cell receptor (BCR) signaling pathway, which promotes lymphomagenesis and the progression of B-cell NHLs^3,4^. Ibrutinib, which irreversibly binds Cys-481 in the ATP-binding pocket of BTK, is an approved second-line therapy for MCL^5^ with a 68% overall response rate and 21% complete response (CR)^6^. However, relapse is inevitable, owing to primary or secondary resistance to ibrutinib^7^. Interestingly, mutations in the BCR pathway (particularly in BTK and its downstream targets, such as *PLCG2*) are usually found in chronic lymphocytic leukemia, whereas mutations in the NF-κB pathway are associated with ibrutinib resistance in MCL^8,9^. Recently, Agarwal et al. first demonstrated that the mutation or deletion of genes encoding the SWI/SNF complex renders patients with MCLs resistant to ibrutinib^10^. However, the mechanism of ibrutinib resistance in MCL is largely unknown. There is a great unmet need in the management of relapsed/refractory MCL; therefore, a deep understanding of the tumor biology and mechanism of lymphomagenesis is necessary to enable the development of novel therapeutic agents.

Although the widespread presence of tumor heterogeneity has been largely confirmed, the biological relationships between cell subpopulations and microenvironments in tumors remain unclear, but may be involved in tumor resistance and recurrence^11^. Compared with traditional methods, single-cell RNA sequencing (scRNA-seq) technology can better address the problem of cell population heterogeneity^12–15^ and provide more information by sequencing of the smallest independent genetic unit of life^16^. In the present study, different cell clusters in the bone marrow of a patient with multidrug resistant MCL were identified. Moreover, we explored the genetic relationships and proliferation potential of intratumoral cells at the levels of differentiation and evolution. After observing the gene expression in bone marrow cells, we identified a series of genes that were associated with immune evasion and drug resistance pathways, and had high expression; these genes may serve as cell markers. Moreover, we further describe the role of these highly expressed genes in immune evasion processes and drug resistance pathways.

## Materials and methods

### Case presentation

A 50 year-old male patient was diagnosed with MCL (stage IV, simplified MIPI score = 3) in 2011. The immunohistochemical results of lymph node biopsy were as follows: CD20+, CD5+, CD23-, CD79a+, Cyclin-D1+, SOX-11+, Bcl-2+, Bcl-6-, CD3-, CD10-, TdT-, MUM-1-, Ki-67 20%+; FISH: t (11; 14) (q13; q32) positive. After diagnosis, he was treated with 6 cycles of R-HyperCVAD/R-MA, and a CR was obtained, which lasted for 24 months. In 2013, the disease relapsed for the first time, and the patient was treated with 3 cycles of bortezomib + rituximab + high-dose cytarabine. Although CR was attained, the collection of autologous stem cells failed. Thalidomide maintenance was initiated until the disease relapsed for the second time 20 months later, in 2015. Subsequently, 2 cycles of R-CHOP/R-DHAP were given, followed by lenalidomide maintenance. In 2017, the patient relapsed for the third time, with a progression free survival (PFS) of 26 months, with pancytopenia and high tumor burden. Afterward, ibrutinib monotherapy was administered at a dose of 560 mg per day. Two months later, CR was confirmed with both PET-CT scan and bone marrow biopsy. Relapse again occurred 7 months later in 2018, with substantial bone marrow infiltration. The ratio of mantle cell lymphoma cells in a bone marrow specimen was 29.5% at the time of sampling. Thus, this patient appeared to be resistant to multiple drugs, including rituximab, cytarabine, bortezomib, lenalidomide and ibrutinib. At that time, the patient had already experienced widespread infiltration of MCL cells (**Supplementary Figure S1**). When he developed resistance to ibrutinib, 10 mL bone marrow aspiration was obtained for scRNA-seq. This study was approved by the ethics committee of Beijing Tongren Hospital (No. TRECKY2018-039), and an informed consent form was signed by the patient.

### Preparation of bone marrow single cell suspension

According to standard operating procedures, mononuclear cells in the bone marrow were isolated with lymphocyte separation fluid (1:1 Ficoll separation solution:anticoagulant). Then, according to the needs of the experiment, phosphate buffered saline (PBS) with 0.5% bovine serum albumin (BSA) was added to adjust the cell concentration for sequencing, and samples were cryopreserved in 90% FBS/10% DMSO for storage in liquid nitrogen.

### 10x sample processing and cDNA library preparation

Samples were prepared as described in the 10x Genomics Single Cell 3′ v2 Reagent Kit user guide. Briefly, the samples were washed twice in PBS (Life Technologies) + 0.04% BSA (Sigma) and re-suspended in PBS + 0.04% BSA. Sample viability was assessed *via* Trypan Blue (Thermo Fisher) and with a hemocytometer (Thermo Fisher). After counting, the appropriate volumes for samples were calculated for a target capture of 6,000 cells and loaded onto a 10× Genomics single-cell-A chip. After droplet generation, samples were transferred into pre-chilled 8-well tubes (Eppendorf) and heat-sealed, and reverse transcription was performed with a Veriti 96-well thermal cycler (Thermo Fisher). After the reverse transcription, cDNA was recovered with Recovery Agent from 10× Genomics, followed by a Silane DynaBead clean-up (Thermo Fisher) as outlined in the user guide. Purified cDNA was amplified for 12 cycles before being cleaned up with SPRIselect beads (Beckman). Samples were diluted 4:1 and analyzed with a Bioanalyzer (Agilent Technologies) to determine cDNA concentration. cDNA libraries were prepared as outlined in the Single Cell 3′ Reagent Kits v2 user guide with appropriate modifications to the PCR cycles on the basis of the calculated cDNA concentration (as recommended by 10× Genomics).

### Sequencing

The molarity of each library was calculated according to library size, as measured with a Bioanalyzer (Agilent Technologies) and qPCR amplification data. Samples were pooled and normalized to 10 nM, then diluted to 2 nM with elution buffer with 0.1% Tween20 (Sigma). Samples were sequenced on a Novaseq 6000 instrument with the following run parameters: read 1, 26 cycles; read 2, 98 cycles; index, 1–8 cycles. A median sequencing depth of 50,000 reads/cell was targeted for samples.

### Sequence filtering and comparison

After Casava base recognition, the original obtained image file was converted into sequenced reads and stored in FASTQ format. The BCL file was split according to the sample index to obtain the FASTQ sequence of each sample. Then the 10X Barcode and UMI sequences were extracted from R1 according to the library structure and 10X Barcode filter. R2 was the insert part (cDNA insert/RNA reads). The RNA reads (inserts) were aligned to the human genome reference sequence with STAR alignment software. Subsequently, the CellRanger (10× Genomics) analysis pipeline was used to generate a digital gene expression matrix from the data. Then, the CellRanger (10× Genomics) analysis pipeline was used to generate a digital gene expression matrix from the data.

### Data processing with the Seurat package

*Seurat* (http://satijalab.org/seurat/)^17^ is an R package allowing users to identify and interpret sources of heterogeneity from single-cell transcriptomic measurements^18^. First, a suitable threshold was determined to filter unwanted cells from the dataset according to the number of unique genes detected in each cell, the total number of molecules detected within a cell and the percentage of reads mapping to the mitochondrial genome. Then the *LogNormalize* method was used to normalize the data. We identified a subset of features that were highly expressed in some cells but weakly expressed in others, exhibiting high cell-to-cell variation in the dataset. By default, we returned 2,000 features per dataset, which were used in downstream analysis. Subsequently, the *FindClusters* function was applied to identify different cell clusters, and the *UMP* method was used for visualization. Moreover, we identified markers for every cluster (compared with all remaining cells) with the *FindAllMarkers* function, retaining only positive genes. The *FindMarkers* function was applied to differential expression analysis and ROC analysis. For each gene, we evaluated (with the AUC) a classifier built on the gene alone, to classify two groups of cells. The *DoHeatmap* function was used to generate an expression heatmap for given cells and features.

### Functional enrichment analysis and calculation of cell stemness index

The *clusterProfiler* package^19^ in R was used for functional enrichment analysis of marker genes in various cell clusters. According to the enriched cancer signal pathway, the specific cell cluster was defined as a malignant B cell cluster. *P* < 0.05 was considered significant. Additionally, under the default parameters, the TCGAbiolinks package^20^ in R was used to calculate the stemness index of each cell.

### Pseudotime analysis

*Monocle* is an R package that introduces the strategy of ordering single cells in pseudotime. Single cells were placed along a trajectory corresponding to a biological process, such as cell differentiation, by taking advantage of an individual cell’s asynchronous progression. This package allows users to determine the track of a cell’s transition from one state to another during development, in the presence of disease and over the lifetime. In our study, data were used for pseudotime analysis with *Monocle 2* after being filtered, normalized and clustered with the Seurat package.

### Exploration of genes involved in the mechanism, in an independent data set

To expand the applicability and value of our research, we examined a clinical cohort of patients with MCL to verify the expression imbalance and clinical prognostic potential of key marker genes involved in the mechanisms (“mechanism genes”). From Gene Expression Omnibus (GEO) (https://www.ncbi.nlm.nih.gov/geo/)^21^, we downloaded 3 datasets related to MCL. Among them, GSE30189 was based on the GPL6884 platform, including 17 MCL samples and 4 control samples; GSE10793 was based on the GPL30789 platform, including 71 MCL samples; and GSE93291 was based on the GPL570 platform, including 123 MCL samples. The *normalizeBetweenArrays* function in the *limma* package^22–24^ was used to normalize gene expression profiles in GSE10793 and GSE93291. Moreover, the *limma* package was used for differential expression analysis, in which *P* < 0.05 was considered significant. Subsequently, we extracted common genes between the set of mechanism genes and differentially expressed genes for clustering analysis. A heatmap of common genes was visualized with the *pheatmap* package (https://cran.r-project.org/web/packages/pheatmap/index.html) and *ggplots* package^25^. In addition, genes in common between the set of mechanism genes and genes from GSE10793 and GSE93291 were selected for survival analysis with the *survfit* function in the *survival* package. The *ggsurvplot* package in the *survminer* package (https://cran.r-project.org/web/packages/survival/index.html) was applied to visualize the survival curve.

## Results

**Figure 1:**
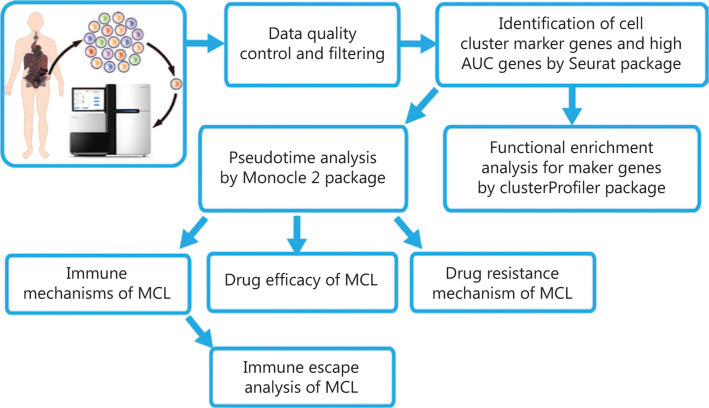
Flow chart of this study.

The flow chart of this study is demonstrated in **Figure 1**.

### Identification of cell types in MCL bone marrow

**Figure 2:**
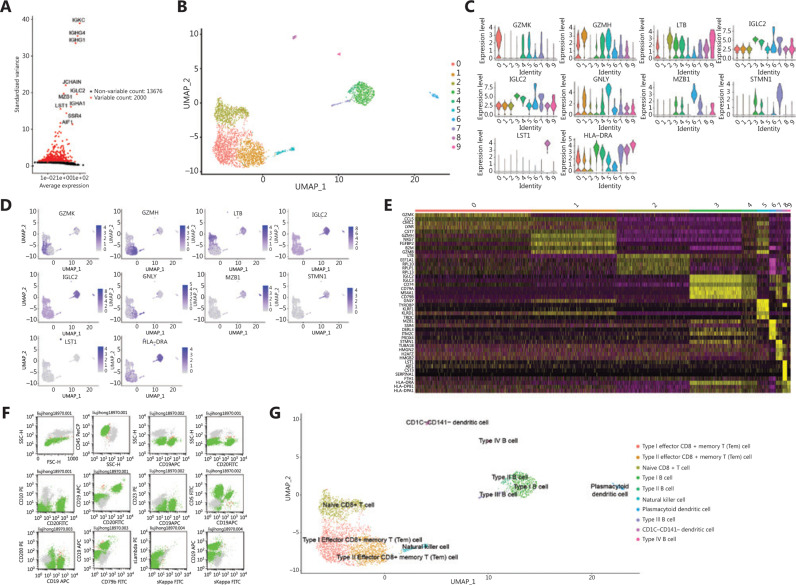
Identification of cell subpopulations in the bone marrow of a patient with mantle cell lymphoma. (A) Characteristic genes with high variation. Red indicates genes with significant differences. (B) Clustering analysis of 10x single-cell transcriptome data from bone marrow cells in patients with mantle cell lymphoma (*n* = 3,989). Each dot represents a single cell and is colored according to cluster, as shown in the key. (C) Expression levels of selected markers, shown in a violin plot. The Supplementary Figure shows the expression levels of marker genes in different clusters. (D) Expression patterns of selected markers projected on the UMAP plot. Blue indicates high expression, and gray indicates low or no expression. For each cell type, one marker is shown in the main figures. (E) Expression heatmap of cell markers in each cluster. Each row represents a gene, and each column represents a single cell. (F) Detection of bone marrow by flow cytometry. Forward scattering (FSC) was used to characterize cell size. Side scattering (SSC) was used to characterize the complexity of cell particles. (G) Identification of cell cluster subpopulations.

A total of 3,989 cells and 15,676 genes were filtered for downstream analysis after removal of low complexity transcriptomes, weakly expressed genes and transcriptomic doublets. In our study, 10 feature genes (highly expressed in some cells and weakly expressed in others) showed high cell-to-cell variation in the dataset: *IGKC*, *IGHG4*, *IGHG1*, *JCHAIN*, *IGLC2*, *MZB1*, *IGHA1*, *LST1*, *SSR4* and *AIF1* (**Figure 2A**). All filtered cells were split into 10 clusters (**Figure 2B**), primarily into clusters 0, 1, 2, 3. Moreover, the marker genes (area under curve (AUC > 0.7) of each cell cluster were identified by ROC analysis (**Supplementary Table S1**). These features were able to serve as biomarkers to distinguish cell clusters with good classification efficiency. In particular, we identified stemness genes from the features, thus suggesting that the dedifferentiation of tumor cells leads to increased stemness (**Supplementary Figure S2A**)^26^. We further identified differentially expressed features of each cell cluster (**Supplementary Table S2, Figure 2C, 2D**) through differential expression analysis. The expression heatmap of features in each cluster showed that these features could be used to classify cell clusters (**Figure 2E**). In addition, we extracted the patient’s bone marrow for flow cytometry and analyzed it with SSC/CD19 gating. The results showed that 84,106/286,279 abnormal cells (29.5% occupied nucleated cells) were positive for expression of *CD5*, *CD19*, *CD20* and λ*-LC*, and negative for expression of *CD10*, *CD23*, *CD38*, *CD56*, *CD79b*, *CD200* and κ*-LC* (**Figure 2F**). According to our analysis, CD19 was mainly expressed in type I and type III B cells (**Supplementary Table S1**). Furthermore, according to the cell markers and cancer-related signaling pathways involved in each cell cluster, the following 10 clusters were identified as specific cell types: 4 malignant B cell subtypes, 3 T cell subtypes, 2 dendritic cell subtypes and 1 NK cell subtype (**Figure 2G, Supplementary Table S3**). We additionally found that many known tumor antigen genes of MCL were differentially over-expressed in four malignant B cell clusters (**Supplementary Figure S2B**). Simultaneously, we investigated the expression of a series of recognized clinical diagnostic markers for MCL, including *CCND1*, *CD5*, *CD19* and *CD200* (**Supplementary Figure S2C**). After mapping their high expression to a single cell atlas, we observed that the cell clusters could be well defined and distinguished, in agreement with our previous results, thus further suggesting that these four cell clusters were malignant B cells.

### The proliferation and differentiation potential of cell clusters

**Figure 3:**
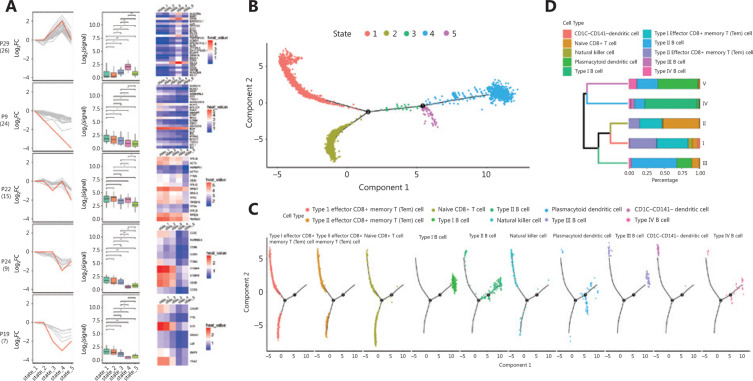
Pseudotime analysis of cell subpopulations in the bone marrow of a patient with mantle cell lymphoma in different states. (A) The expression of characteristic gene sets in each state. Line plots (left panels) and box plots (right panels) show fold changes (log_2_ scale) and absolute expression levels (log_2_ scale), respectively. In each line plot, one representative gene is highlighted in red. (B) The ordering of different cell clusters along pseudotime in a two-dimensional state-space defined by Monocle2. Cell orders were inferred from the expression of the most dispersed genes across cell clusters. Each point corresponds to a single cell, and each color represents a state. (C) Distribution of cell clusters on quasi-time trajectories. Each color represents a state. (D) Unsupervised hierarchical clustering of the 10 clusters according to the state of cells in each cluster. The proportions of different cell sources are shown.

To further study the potential relationships among cell clusters in MCL, we first identified the characteristic gene sets reflecting cell states for each cell cluster (**Figure 3A**). According to similarity of gene expression, cells were clustered and formed a relative cell trajectory in the simulation time *via* pseudotime analysis. The trajectory showed that these cells could be divided into five different states (**Figure 3B**). A closer relationship was observed between state 1 and state 2, as well as between state 4 and state 5 (**Figure 3C**). Malignant B cells were concentrated in state 4, and plasmacytoid dendritic cells were mainly distributed in states 4 and 5, whereas other microenvironment cells were concentrated in state 1 (**Figure 3D, Supplementary Figure S3A, 3B**). Unexpectedly, we found a higher differentiation potential in type I and IV B cells that had lower maturity and higher malignancy (**Supplementary Figure S3C**).

### Immunological mechanism of the microenvironment

Immunoglobulins are produced when the body is stimulated by antigens, such as pathogens. Its main function is to react with antigens, thus producing antigen antibody complexes, blocking the invasion of pathogens into human body and causing pathogens to lose their pathogenicity. In this study, the abnormal elevation of IgG and IgE in the patient indicated that his body had been stimulated by antigens (**Supplementary Table S4**); this response might have been associated with immune mechanisms. Notably the autoimmune hemolytic complications in this patient during treatment were associated with the increased expression of immunoglobulin Fc receptor genes (such as *FCRLA*, *FCMR*, *FCGRT*, *FCER2*, *FCGR2B* and *FCRL2*). After IgG sensitization of red blood cells (RBCs), Fc receptors on the macrophage surface can recognize and bind RBCs^27^, thus resulting in autoimmune hemolysis. In addition, T-helper cells and their cytokines play a major role in regulating IgE synthesis^28–30^. T helper cells are subsets of T cells that have important roles in the differentiation of immune cells, the binding of effector cell subsets and the induction of responses^31^. Autoimmune hemolysis has been suggested to be associated with disorder in the immune environment.

**Figure 4:**
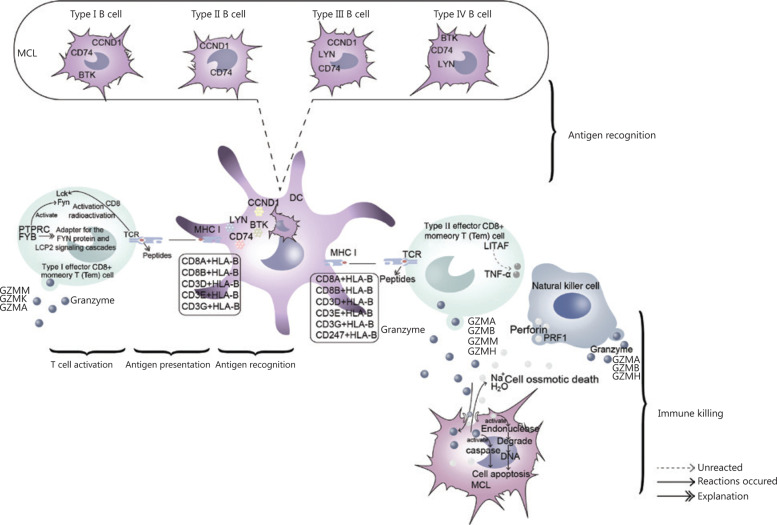
Immune mechanisms of the MCL microenvironment. As indicated in the figure, this mechanism mainly included antigen recognition, antigen presentation, T cell activation and immune killing.

Furthermore, we explored the potential immune mechanism of MCL in the bone marrow microenvironment (**Figure 4, Supplementary Figure S4**). During antigen recognition, tumor cells are phagocytized and degraded into peptide segments by dendritic cells^32–35^. The degraded peptide segments bind MHC1 molecules and form the MHC1 class complex^36^. This complex interacts with T cell receptors (TCRs) and induces activation of the TCR signaling pathway, thereby eventually leading to the T cell immune response^37^. Activation of the Src family kinases Lck and Fyn is key in activation of the TCR signaling pathway. In type I T cells, the elevated expression of FYB and PTRPC, a CD8 synergistic receptor, enhances the activation of FYN and Lck, respectively, thus promoting signal transduction of T cell activation and the expression of associated genes. In terms of immune killing, *PRF1* (perforin-coding gene) was highly expressed in NK cells. Perforin with Ca^2+^ can form channels on target cells, and the cells are subsequently dissolved osmotically^38–40^. However, we observed high expression of the granzyme genes *GZMM*, *GZMA*, *GZMB*, *GZMH* and *GZMK* in type I T cells, type II T cells and NK cells. In cooperation with perforin, released granzyme acts on the nucleus and triggers a caspase cascade reaction, thus causing apoptosis^41^.

### Immune escape of tumor cells

**Figure 5:**
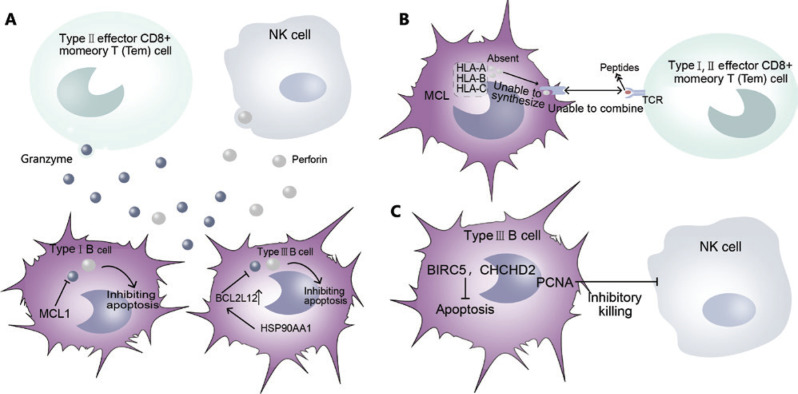
The immune escape mechanism of MCL cells. (A) Anti-perforin. (B) Low immunogenicity. (C) Inhibition of NK cell killing.

The occurrence of cancer is closely associated with the immune function of the body, which is activated when it encounters foreign invaders. Therefore, when cancer has occurred and developed, cancer cells can survive and proliferate *in vivo* by avoiding recognition and attack by the body’s immune system. Numerous clinical data have shown that low or suppressed immune function in the host often leads to an increase in tumor incidence. In this study, we identified several potential immune escape mechanisms of MCL malignant cells (**Supplementary Figure S5**), such as the anti-perforin pathway (**Figure 5A**) and the low tumor cell immunogenicity pathway (**Figure 5B**). On the one hand, the overexpression of BCL2 family members (MCL1 in type I B cells and BCL2 L12 in type III B cells) inhibits perforin and decreases the damage caused by cancer cells. In addition, high expression of HSP90AA1 further promotes the expression of BCL2 L12. On the other hand, the lack of MHCI class genes (including HLA-A, HLA-B and HLA-C) in MCL malignant cells leads to an absence of MHC complexes, thus avoiding recognition by type I, II T cells. In addition, BIRC5, CHD2 and PCNA are highly expressed in type III B cells. BIRC5 and CHCHD2 inhibit cell apoptosis, whereas PCNA inhibits the killing effects of NK cells, thus promoting the survival of malignant cells by inhibiting cell apoptosis and cell killing (**Figure 5C**), in another potential immune escape mechanism of malignant cells.

### Multidrug resistance of tumor cells

**Figure 6:**
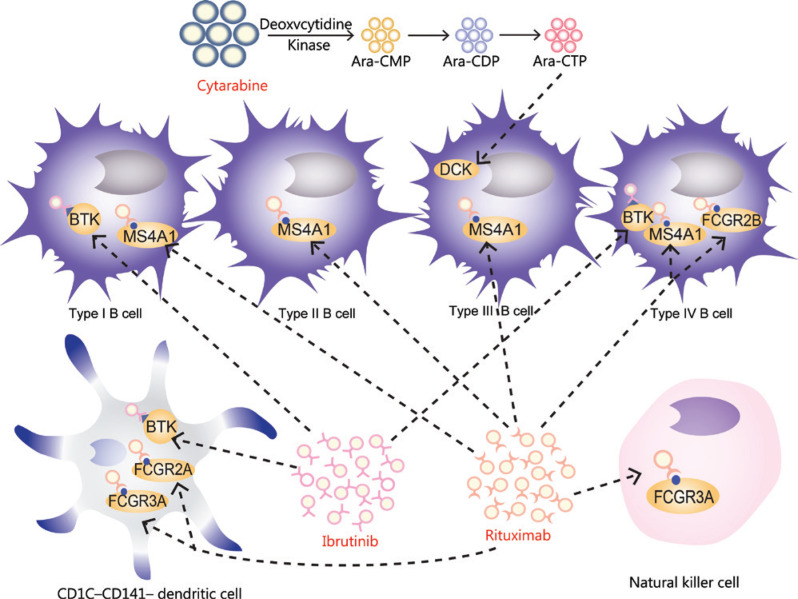
Drug target genes are highly expressed in cell clusters.

Drug resistance is one of the principal reasons for the failure of anti-infection drugs and cancer chemotherapy. In addition to determining the expression and functions of genes in cells, this study further elucidated the potential drug resistance mechanism in MCL at the cell level. First, some drug target genes were found to be highly expressed in the patient (**Figure 6**). For example, BTK is a gene upstream of the BCR signaling pathway, whose encoded protein is essential for the development, differentiation and signal transduction of B lymphocytes^3,42^. Ibrutinib inhibits the proliferation of malignant B cells by inhibiting BTK^43,44^. MS4A1 (also called CD20) encodes a B lymphocyte surface molecule involved in the development and differentiation of B cells into plasma cells^45,46^. Rituximab specifically binds MSA1, thus inactivating it or causing its shedding from the cell surface^47^. In addition, FC receptors on B cells promote rituximab internalization, thereby decreasing its clinical efficacy^48^. Moreover, we observed high expression of FC receptor-encoding genes, including FCGR2A, FCGR2B and FCGR3A. In conclusion, high expression of these drug target genes indicated the emergence of drug resistance and the enhancement of B cell viability during drug resistance.

**Figure 7:**
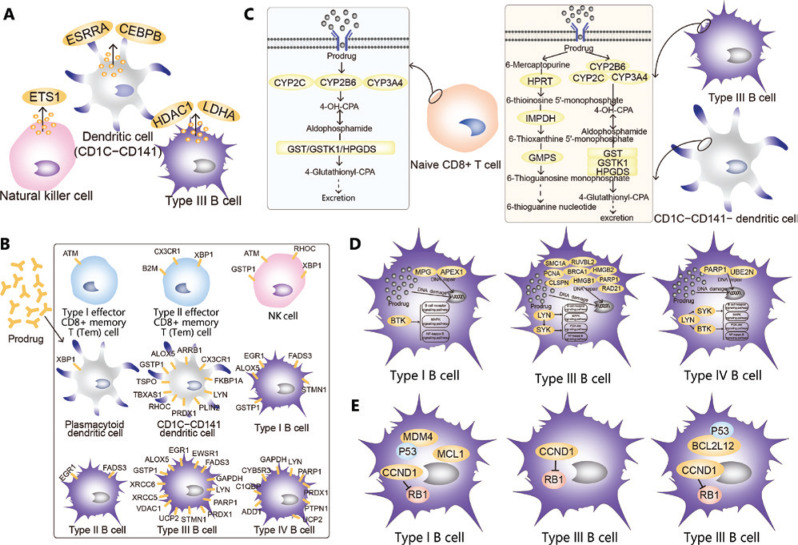
Potential mechanisms of drug resistance in MCL. (A) Secretory protein and extracellular matrix related drug resistance. Microenvironment cells protected malignant B cells from drug resistance by secreting proteins and extracellular matrix. (B) Cell surface receptors associated with drug resistance. Cell surface receptors bound to drugs, thus reducing drug concentrations in cells and diminishing the toxicity of drugs to cancer cells. (C) Drug resistance related to drug metabolism. Drug metabolic pathways led to drug resistance. (D) Survival promotion and DNA damage repair related to drug resistance. Malignant B cell resistance induced by DNA damage repair and survival promotion. (E) Cancer gene related drug resistance. Malignant B cells promoted their survival by inhibiting the activity of tumor suppressor proteins, thus resulting in drug resistance.

Specific niches within the tumor microenvironment of lymphoma provide shelter for subpopulations of cancer cells through interactions between stromal cells and tumor cells, thus leading to drug resistance in malignant B cells^49,50^. This mechanism is mainly mediated by chemokines and integrins, which are associated with the extracellular matrix^51^. Here (**Supplementary Figure S6**), we found many genes encoding secretory proteins and extracellular matrix proteins in NK cells, CD1C CD141^−^ dendritic cells and type III B cells, thus indicating that malignant B cells may be protected by these proteins in the development of drug resistance (**Figure 7A**). In addition, drug-binding receptors on the cell surface might decrease the concentrations of drugs entering the cell, thus diminishing the toxicity of the drugs to cancer cells and promoting cell survival (**Figure 7B**). In naive CD8^+^ T cells, type III B cells and CD1C CD141^−^ dendritic cells, we identified significant overexpression of genes participating in drug metabolism, including cytochrome P450 and other enzymes (**Figure 7C**), thus suggesting that these cells might be resistant to drugs through drug metabolism. In addition, genes associated with DNA damage repair were significantly overexpressed in type I B, type III B and type IV B cells (**Figure 7D**). Considerable evidence in solid tumors suggests that increased repair or tolerance of DNA lesions may contribute to the ability of cancer cells to survive in environments with high genotoxic stress^52^. Various oncogenes, cancer stem cells, a hypoxic environment, transcription factors and bystander signaling are activated in cancer cells, thus allowing for effective repair of DNA damage^53^. These repaired cancer cells are often more resistant to further treatment, thus leading to disease recurrence^53^. In this study, the genes associated with DNA damage repair were highly expressed, thereby suggesting that the recurrence of cancer and resistance might be due to DNA damage repair. Moreover, BTK was highly expressed in type I and IV B cells, whereas LYN and SYK were highly expressed in type III and IV B cells. These genes are upstream of survival pathways, such as the B cell receptor, MAPK, NF-kappa B and PI3K-Akt signaling pathways^4,54^; therefore, these 3 types of malignant B cells resist drugs and promote cell survival (**Figure 7D**). In addition, type I, II and IV B cells produce MDM4, CCND1 and MCL1 proteins, which act on or bind P53, RB1 and other anti-cancer proteins^55–57^, and consequently decrease the activity of anti-cancer proteins, thus promoting cell survival (**Figure 7E**).

## Discussion

MCL is a highly aggressive B cell malignant lymphoma^58^. Frequent recurrence and drug resistance during treatment are major challenges in defining standard therapies, because of MCL’s biological and clinical heterogeneity^59,60^. In this study, bone marrow samples were collected from a multidrug resistant patient, and 10 cell clusters were identified by scRNA-seq: 4 malignant B cell clusters, 3 T cell clusters, 2 dendritic cell clusters and 1 NK cell cluster. Subsequently, we identified the potential mechanism of immune escape and multidrug resistance in MCL. However, because of the small number of sequenced specimens (*n* = 1), we validated the main conclusions in a larger sample size, in a clinical cohort of patients with MCL from the GEO database.

Tumorigenesis has been proposed to arise from the orderly and uncontrolled differentiation potential of stem cells, whereas tumor cells originating from early stem cells have greater proliferation and differentiation potential^61^. In this study, pseudotime analysis showed that these cell clusters might arise from the same ancestors. In addition, we observed greater proliferation and differentiation potential of type I B cells than other clusters, thus indicating that these cells have greater potential for immune escape and drug resistance mechanisms, and therefore might be used as drug targets in the future. We speculate that the different proliferation and differentiation potentials of cancer cells confer them with different advantages in immune escape and drug resistance mechanisms. Additionally, on the basis of the cell markers, we found high heterogeneity in each cell cluster (**Supplementary Figure S4**), which is the main driving force underlying tumor development, metastasis and drug resistance^62^. Tumor cells are genetically unstable, owing to somatic mutations, which eventually lead to subclones with different mutations and manifestations^63,64^. Under the pressure of specific chemotherapeutic drugs, heterogeneous tumors can select subclones with intrinsic or acquired drug resistance to ensure the survival of tumor cells, thus promoting disease development^65^. Therefore the relapse of disease appears to be closely associated with the high heterogeneity of MCL. Further analysis revealed that the differentially expressed genes shared by 4 malignant cell types were mainly involved in biological processes such as immune response, antigen presentation, B cell receptor signal transduction, and promotion of B cell survival *in vitro* (**Supplementary Figure S7C**). These findings indicated that these malignant B cells play an important role in the malignant process of MCL, for example in immune escape and drug resistance.

Further investigations showed that malignant cells of MCL mainly avoid immune killing *via* inhibiting perforin, decreasing autoimmunity and directly inhibiting apoptosis or NK cell killing. Perforin is a cytotoxic molecule in the body, and the perforin-dependent pathway is the main mechanism of killing cancer cells^39,66^. BCL2 inhibits the apoptotic pathway of perforin and granulase B^67^. In this study, the high expression of BCL2 family genes suggested that the BCL2 family might play a major role in the immune escape of MCL. In the follow-up treatment of our patient, his condition was completely alleviated after the use of the BCL-2 inhibitor venetoclax, thus verifying our hypothesis and confirming our conclusion regarding the immune escape mechanism.

High expression of some targeted genes by resistant drugs might signify that the effect of drugs on target genes is weakened or ineffective. On one hand, a change in drug targets or the consumption of drugs in metabolic pathways may occur, thus preventing the drugs’ standard actions. On the other hand, the high heterogeneity of MCL malignant cells might play a role when cells are first treated by drugs. As a result of division and proliferation, daughter cells show changes in molecular biology or genes, thus leading to drug resistance. In contrast, other target genes of drugs were not found to be highly expressed, possibly because of partial drug efficacy or low concentrations of drugs in cells. According to the correlations among the molecular characteristics of the cell clusters (**Supplementary Figure S7D**), we found that type III B cells and plasmacytoid dendritic cells communicated more closely with other cells. We speculated that type III B cells, as malignant cells with active molecular communication, play a major role in both immune escape and drug resistance. In addition, plasmacytoid dendritic cells might act as antigen presenting cells, activating immune killer cells (mainly T cells), which in turn play a bridging role in the immune mechanisms specific to the microenvironment.

In the treatment of cancer, drug resistance has a major role in the recurrence and incurable status of cancer. There are many reasons for drug resistance, such as the tumor microenvironment, cancer stem cells, inactivation of anti-cancer drugs, increased drug efflux, reduced drug absorption, improved drug metabolism and drug target gene mutation^68–70^. In previous studies, overexpression of cyclin D1 and Bcl-2 has been considered to be the cause of cell cycle disorder and the decrease in apoptotis^71^, effects closely associated with drug resistance. In addition, we identified different resistance mechanisms in different cell clusters, thus possibly explaining why traditional chemotherapy is not effective for some patients. After studying independent datasets of an MCL cohort, we identified abnormal expression of the genes involved in these mechanisms (**Supplementary Figure S8A**). Moreover, most genes involved in these mechanisms, particularly CCND1 and ALOX5, were significantly associated with the prognosis of MCL (**Supplementary Figure S8B, S8C**). These genes not only were abnormally expressed in MCL but also were significantly correlated with the prognosis of MCL. *CCND1* is associated with a variety of cancers^72–75^, and its abnormal expression is associated with the drug resistance of MCL^56^; therefore, this gene may become a potential therapeutic target for MCL. In addition, ALOX5 is a key gene effector of JAK2V617F driving PV, thus making it a candidate therapeutic target for the treatment of refractory myeloproliferative tumors^76^. Here, we found that ALOX5 was associated with the immune mechanism of MCL, thus providing new ideas and strategies for further research on MCL.

## Conclusions

The high heterogeneity of malignant MCL cells enables them to evade immune attack and drug toxicity through different mechanisms, thus leading to drug resistance and a high recurrence rate. According to our findings, in clinical practice, MCL may be treated with strategies targeting different cell components, such as inhibiting drug metabolism or anti-apoptotic pathways. Although our research was mainly based on the analysis of bioinformatics and validation in a clinical cohort of patients with MCL from the GEO database in several molecular experiments, our work may provide multiple candidates for investigation in future research on drug resistance mechanisms in MCL.
