# Supplementary material for: Single-cell RNA-seq reveals the immune escape and drug resistance mechanisms of mantle cell lymphoma
Source: Cancer Biol Med. 2020 Aug 15;17(3):726–39. doi: 10.20892/j.issn.2095-3941.2020.0073 (PMC7476085; doi:10.20892/j.issn.2095-3941.2020.0073)
Supplement: Supplementary file 1 [file cbm-17-726-s001.pdf]

Supplementary materials

Table S1 Marker genes for each cell subpopulation

Gene_marker	myAUC	Avg_diff	Power	Avg_logFC	Pct.1	Pct.2	*P*_val_adj	Cluster
GZMK	0.841	1.663182738	0.682	1.663182738	0.796	0.211	NA	0
CCL5	0.802	0.792291655	0.604	0.792291655	0.997	0.816	NA	0
CMC1	0.744	0.972272686	0.488	0.972272686	0.78	0.394	NA	0
LYAR	0.711	0.783510565	0.422	0.783510565	0.655	0.282	NA	0
CST7	0.702	0.477310958	0.404	0.477310958	0.91	0.597	NA	0
GZMH	0.916	1.510234259	0.832	1.510234259	0.969	0.444	NA	1
NKG7	0.877	1.05303263	0.754	1.05303263	0.999	0.785	NA	1
FGFBP2	0.827	1.485499676	0.654	1.485499676	0.765	0.187	NA	1
B2M	0.815	0.303392763	0.63	0.303392763	1	1	NA	1
GZMB	0.797	1.285614678	0.594	1.285614678	0.727	0.207	NA	1
CST7	0.77	0.710949366	0.54	0.710949366	0.951	0.617	NA	1
CCL5	0.757	0.623697673	0.514	0.623697673	0.998	0.835	NA	1
GZMA	0.756	0.736590732	0.512	0.736590732	0.915	0.553	NA	1
SH3BGRL3	0.753	0.544690885	0.506	0.544690885	0.991	0.902	NA	1
KLRD1	0.751	0.903127928	0.502	0.903127928	0.774	0.36	NA	1
S100A4	0.75	0.602483617	0.5	0.602483617	0.984	0.841	NA	1
PRF1	0.737	0.89105827	0.474	0.89105827	0.693	0.296	NA	1
ACTB	0.737	0.454706549	0.474	0.454706549	1	1	NA	1
CTSW	0.736	0.675718729	0.472	0.675718729	0.862	0.529	NA	1
CD3D	0.723	0.515111944	0.446	0.515111944	0.973	0.787	NA	1
IL32	0.708	0.44979189	0.416	0.44979189	0.992	0.822	NA	1
HCST	0.705	0.510270528	0.41	0.510270528	0.924	0.777	NA	1
HLA-C	0.705	0.316764661	0.41	0.316764661	1	0.998	NA	1
LTB	0.846	1.262633582	0.692	1.262633582	0.926	0.474	NA	2
EEF1A1	0.797	0.346447159	0.594	0.346447159	1	1	NA	2
RPL10	0.792	0.346185404	0.584	0.346185404	1	1	NA	2
RPLP1	0.787	0.400891514	0.574	0.400891514	1	1	NA	2
RPL13	0.785	0.319839963	0.57	0.319839963	1	1	NA	2

Table S1 Continued

Gene_marker	myAUC	Avg_diff	Power	Avg_logFC	Pct.1	Pct.2	*P*_val_adj	Cluster
RPL32	0.776	0.33152658	0.552	0.33152658	1	1	NA	2
RPS25	0.773	0.386838669	0.546	0.386838669	1	0.998	NA	2
RPL34	0.768	0.357749148	0.536	0.357749148	1	1	NA	2
RPS6	0.766	0.32181637	0.532	0.32181637	1	1	NA	2
RPL21	0.757	0.299862331	0.514	0.299862331	1	1	NA	2
IL7R	0.754	1.01777479	0.508	1.01777479	0.733	0.281	NA	2
RPS8	0.753	0.339865853	0.506	0.339865853	1	0.999	NA	2
RPL36	0.746	0.320563065	0.492	0.320563065	1	1	NA	2
RPS18	0.746	0.28150499	0.492	0.28150499	1	1	NA	2
LDHB	0.744	0.813717458	0.488	0.813717458	0.832	0.53	NA	2
TPT1	0.744	0.4321877	0.488	0.4321877	0.996	0.977	NA	2
RPS27A	0.743	0.299561708	0.486	0.299561708	1	1	NA	2
FXYD5	0.73	0.7250378	0.46	0.7250378	0.825	0.555	NA	2
RPS3A	0.729	0.304044778	0.458	0.304044778	1	1	NA	2
RPL18A	0.728	0.277247751	0.456	0.277247751	1	1	NA	2
RPL39	0.727	0.27774111	0.454	0.27774111	1	1	NA	2
RPL11	0.724	0.259302292	0.448	0.259302292	1	1	NA	2
RPL36A	0.722	0.374102656	0.444	0.374102656	0.999	0.986	NA	2
RPS12	0.722	0.286011451	0.444	0.286011451	1	1	NA	2
RPL9	0.714	0.321438143	0.428	0.321438143	1	0.996	NA	2
RPL12	0.708	0.275941409	0.416	0.275941409	0.999	0.999	NA	2
RPL35A	0.705	0.255497185	0.41	0.255497185	1	1	NA	2
RPS13	0.704	0.302885047	0.408	0.302885047	0.999	0.997	NA	2
RPL31	0.703	0.272156715	0.406	0.272156715	1	0.999	NA	2
IGLC2	0.985	1.568590115	0.97	1.568590115	1	0.939	NA	3
IGLC3	0.981	1.661713956	0.962	1.661713956	0.996	0.412	NA	3
CD74	0.958	1.326480013	0.916	1.326480013	1	0.928	NA	3
CD79A	0.954	2.108262151	0.908	2.108262151	0.956	0.164	NA	3
MS4A1	0.953	2.241975953	0.906	2.241975953	0.951	0.144	NA	3
CD79B	0.948	1.996487639	0.896	1.996487639	0.953	0.215	NA	3

Table S1 Continued

Gene_marker	myAUC	Avg_diff	Power	Avg_logFC	Pct.1	Pct.2	*P*_val_adj	Cluster
HLA-DRA	0.942	1.679595904	0.884	1.679595904	0.998	0.558	NA	3
IGLC7	0.932	2.212569393	0.864	2.212569393	0.909	0.122	NA	3
IGHM	0.925	1.031200992	0.85	1.031200992	0.951	0.378	NA	3
IGLC6	0.922	2.374780226	0.844	2.374780226	0.88	0.066	NA	3
CCND1	0.914	2.014724192	0.828	2.014724192	0.874	0.101	NA	3
CD69	0.908	1.595866207	0.816	1.595866207	0.958	0.55	NA	3
VPREB3	0.903	1.935116145	0.806	1.935116145	0.852	0.09	NA	3
BTG2	0.893	1.72931512	0.786	1.72931512	0.882	0.227	NA	3
H3F3B	0.87	0.978617323	0.74	0.978617323	0.991	0.953	NA	3
EGR1	0.869	1.78657996	0.738	1.78657996	0.796	0.109	NA	3
IER2	0.869	1.481617247	0.738	1.481617247	0.925	0.522	NA	3
SMIM14	0.847	1.644623051	0.694	1.644623051	0.749	0.097	NA	3
CD37	0.841	0.974961817	0.682	0.974961817	0.942	0.696	NA	3
MT-ND4	0.839	0.639351731	0.678	0.639351731	0.998	0.984	NA	3
RPS11	0.833	0.596069729	0.666	0.596069729	0.995	0.981	NA	3
MT-ND3	0.825	0.626965758	0.65	0.626965758	1	0.978	NA	3
KLF6	0.82	1.060020331	0.64	1.060020331	0.922	0.646	NA	3
JUN	0.813	1.07909617	0.626	1.07909617	0.951	0.731	NA	3
CD24	0.802	1.474685646	0.604	1.474685646	0.638	0.049	NA	3
ZFP36L1	0.802	1.200476064	0.604	1.200476064	0.821	0.363	NA	3
MARCKSL1	0.777	1.185481393	0.554	1.185481393	0.672	0.169	NA	3
TNFRSF13C	0.768	1.307013475	0.536	1.307013475	0.579	0.063	NA	3
RPS5	0.766	0.443319731	0.532	0.443319731	0.996	0.985	NA	3
FOS	0.763	1.154297491	0.526	1.154297491	0.787	0.428	NA	3
DUSP2	0.76	1.174507654	0.52	1.174507654	0.812	0.528	NA	3
CH17-373J23.1	0.753	1.250636827	0.506	1.250636827	0.645	0.188	NA	3
MALAT1	0.753	0.334287	0.506	0.334287	1	1	NA	3
RPS23	0.75	0.343238572	0.5	0.343238572	1	1	NA	3
HLA-DMA	0.748	1.018449331	0.496	1.018449331	0.683	0.243	NA	3
CD9	0.74	1.245715836	0.48	1.245715836	0.512	0.044	NA	3

Table S1 Continued

Gene_marker	myAUC	Avg_diff	Power	Avg_logFC	Pct.1	Pct.2	*P*_val_adj	Cluster
RP5-887A10.1	0.739	1.408687628	0.478	1.408687628	0.506	0.035	NA	3
FCRL2	0.738	1.183046235	0.476	1.183046235	0.514	0.047	NA	3
FAM129C	0.737	1.186769319	0.474	1.186769319	0.506	0.04	NA	3
ARHGAP24	0.737	1.171619277	0.474	1.171619277	0.503	0.036	NA	3
CLECL1	0.734	1.141155999	0.468	1.141155999	0.514	0.06	NA	3
GNG11	0.733	1.181735457	0.466	1.181735457	0.497	0.04	NA	3
DUSP1	0.732	0.72726576	0.464	0.72726576	0.905	0.711	NA	3
HLA-DQA2	0.729	0.806894695	0.458	0.806894695	0.734	0.334	NA	3
LY86	0.728	1.095702368	0.456	1.095702368	0.488	0.04	NA	3
HLA-DMB	0.728	1.018478384	0.456	1.018478384	0.548	0.112	NA	3
IGHD	0.727	1.126875383	0.454	1.126875383	0.49	0.044	NA	3
RP11-386I14.4	0.726	1.08388621	0.452	1.08388621	0.661	0.285	NA	3
RP11-231C14.7	0.721	1.124857811	0.442	1.124857811	0.475	0.042	NA	3
TCL1A	0.719	1.13480931	0.438	1.13480931	0.47	0.036	NA	3
TSC22D3	0.719	0.556829576	0.438	0.556829576	0.929	0.762	NA	3
PTMA	0.719	0.354679733	0.438	0.354679733	1	0.999	NA	3
GSTP1	0.718	0.777266861	0.436	0.777266861	0.778	0.493	NA	3
RHOB	0.717	1.260208226	0.434	1.260208226	0.479	0.06	NA	3
CD19	0.717	1.085021295	0.434	1.085021295	0.481	0.061	NA	3
NCF1	0.717	0.91275865	0.434	0.91275865	0.556	0.151	NA	3
ITM2C	0.716	1.012729233	0.432	1.012729233	0.532	0.122	NA	3
MT-ND1	0.715	0.563116939	0.43	0.563116939	0.938	0.775	NA	3
MT-CO2	0.712	0.346669126	0.424	0.346669126	0.998	0.997	NA	3
CXCR4	0.709	0.738836566	0.418	0.738836566	0.803	0.568	NA	3
SEC62	0.706	0.732654752	0.412	0.732654752	0.743	0.437	NA	3
SMARCB1	0.703	0.912583928	0.406	0.912583928	0.519	0.142	NA	3
IGLC2	0.836	0.347225889	0.672	0.347225889	1	0.945	NA	4
IGLC3	0.821	0.295402194	0.642	0.295402194	0.976	0.472	NA	4
CD79B	0.804	0.71033081	0.608	0.71033081	0.915	0.29	NA	4
MS4A1	0.798	0.673833584	0.596	0.673833584	0.878	0.228	NA	4

Table S1 Continued

Gene_marker	myAUC	Avg_diff	Power	Avg_logFC	Pct.1	Pct.2	*P*_val_adj	Cluster
CD74	0.798	0.598285058	0.596	0.598285058	1	0.936	NA	4
BTG2	0.792	0.795356092	0.584	0.795356092	0.854	0.294	NA	4
CCND1	0.787	0.767314033	0.574	0.767314033	0.799	0.182	NA	4
MT-ND4	0.783	0.450337734	0.566	0.450337734	1	0.985	NA	4
CD79A	0.778	0.634841931	0.556	0.634841931	0.848	0.249	NA	4
IGLC7	0.765	0.679873532	0.53	0.679873532	0.774	0.207	NA	4
HLA-DRA	0.762	0.537652465	0.524	0.537652465	0.963	0.604	NA	4
EGR1	0.755	0.790014526	0.51	0.790014526	0.713	0.182	NA	4
IER2	0.751	0.60201819	0.502	0.60201819	0.945	0.562	NA	4
H3F3B	0.744	0.46571021	0.488	0.46571021	1	0.956	NA	4
KLF6	0.741	0.582141464	0.482	0.582141464	0.945	0.673	NA	4
MT-ND3	0.736	0.380952708	0.472	0.380952708	1	0.98	NA	4
IGLC6	0.733	0.481392546	0.466	0.481392546	0.671	0.157	NA	4
VPREB3	0.731	0.670434248	0.462	0.670434248	0.665	0.174	NA	4
MALAT1	0.723	0.260883932	0.446	0.260883932	1	1	NA	4
CD69	0.712	0.460281783	0.424	0.460281783	0.902	0.594	NA	4
CXCR4	0.702	0.565701781	0.404	0.565701781	0.915	0.587	NA	4
MT-ND1	0.702	0.413669242	0.404	0.413669242	0.976	0.79	NA	4
GNLY	0.978	2.723132533	0.956	2.723132533	0.992	0.415	NA	5
TYROBP	0.932	2.151456962	0.864	2.151456962	0.901	0.078	NA	5
KLRF1	0.924	2.242124133	0.848	2.242124133	0.863	0.032	NA	5
KLRD1	0.888	1.263424072	0.776	1.263424072	0.962	0.437	NA	5
TRDC	0.879	1.931205686	0.758	1.931205686	0.779	0.033	NA	5
GZMB	0.876	1.784732992	0.752	1.784732992	0.916	0.305	NA	5
FCER1G	0.871	1.855822643	0.742	1.855822643	0.779	0.044	NA	5
ID2	0.85	1.213664129	0.7	1.213664129	0.924	0.422	NA	5
NKG7	0.847	0.924740766	0.694	0.924740766	0.992	0.828	NA	5
CTSW	0.843	0.991041423	0.686	0.991041423	0.969	0.592	NA	5
SRGN	0.816	0.886395147	0.632	0.886395147	0.954	0.702	NA	5
IFITM2	0.815	1.090718996	0.63	1.090718996	0.901	0.585	NA	5

Table S1 Continued

Gene_marker	myAUC	Avg_diff	Power	Avg_logFC	Pct.1	Pct.2	*P*_val_adj	Cluster
KLRC1	0.812	2.043367204	0.624	2.043367204	0.634	0.017	NA	5
CD7	0.808	1.217271549	0.616	1.217271549	0.832	0.4	NA	5
HOPX	0.803	1.291944956	0.606	1.291944956	0.748	0.216	NA	5
CMC1	0.791	0.969731961	0.582	0.969731961	0.901	0.5	NA	5
PLAC8	0.78	1.197874936	0.56	1.197874936	0.695	0.184	NA	5
IFITM3	0.732	1.125425877	0.464	1.125425877	0.527	0.07	NA	5
PRF1	0.732	0.941528478	0.464	0.941528478	0.748	0.374	NA	5
CST7	0.727	0.607811752	0.454	0.607811752	0.931	0.685	NA	5
CD63	0.72	0.811796574	0.44	0.811796574	0.695	0.336	NA	5
GZMA	0.713	0.559437474	0.426	0.559437474	0.939	0.625	NA	5
HCST	0.71	0.480784124	0.42	0.480784124	0.962	0.806	NA	5
IL2RB	0.709	0.970288789	0.418	0.970288789	0.542	0.143	NA	5
KLRB1	0.701	0.923972139	0.402	0.923972139	0.595	0.215	NA	5
MZB1	0.998	2.711108644	0.996	2.711108644	1	0.178	NA	6
SSR4	0.996	1.877800812	0.992	1.877800812	1	0.645	NA	6
DERL3	0.96	1.644166938	0.92	1.644166938	0.973	0.065	NA	6
ITM2C	0.922	1.304086053	0.844	1.304086053	0.986	0.164	NA	6
PRDX4	0.903	1.250268974	0.806	1.250268974	0.877	0.08	NA	6
XBP1	0.894	1.361604091	0.788	1.361604091	0.959	0.311	NA	6
TNFRSF17	0.884	1.110566148	0.768	1.110566148	0.781	0.009	NA	6
HERPUD1	0.882	1.244777479	0.764	1.244777479	0.986	0.333	NA	6
FKBP11	0.873	1.292225199	0.746	1.292225199	0.904	0.255	NA	6
FKBP2	0.859	1.058075859	0.718	1.058075859	0.932	0.214	NA	6
JCHAIN	0.855	4.089093936	0.71	4.089093936	0.795	0.113	NA	6
SEC11C	0.853	1.057319406	0.706	1.057319406	0.918	0.22	NA	6
UBE2J1	0.852	0.947665303	0.704	0.947665303	0.863	0.123	NA	6
HSP90B1	0.84	1.03043488	0.68	1.03043488	0.973	0.365	NA	6
IGHG4	0.839	4.459287464	0.678	4.459287464	1	0.974	NA	6
IGKC	0.838	4.795639548	0.676	4.795639548	1	0.995	NA	6
IGHG1	0.838	4.656961088	0.676	4.656961088	0.959	0.764	NA	6

Table S1 Continued

Gene_marker	myAUC	Avg_diff	Power	Avg_logFC	Pct.1	Pct.2	*P*_val_adj	Cluster
TXNDC11	0.838	0.702708868	0.676	0.702708868	0.781	0.068	NA	6
EAF2	0.831	0.74974738	0.662	0.74974738	0.685	0.016	NA	6
CD38	0.831	0.588450598	0.662	0.588450598	0.753	0.056	NA	6
SPCS3	0.821	0.808667372	0.642	0.808667372	0.863	0.173	NA	6
DNAJB9	0.821	0.733527434	0.642	0.733527434	0.822	0.123	NA	6
MEF2C	0.814	0.589655995	0.628	0.589655995	0.726	0.064	NA	6
NPC2	0.81	0.617570744	0.62	0.617570744	0.904	0.191	NA	6
SSR3	0.806	0.714707297	0.612	0.714707297	0.863	0.184	NA	6
MANF	0.803	0.790530232	0.606	0.790530232	0.877	0.202	NA	6
PDIA4	0.801	0.768006518	0.602	0.768006518	0.836	0.174	NA	6
SEL1L3	0.801	0.556122924	0.602	0.556122924	0.699	0.065	NA	6
GNG7	0.798	0.611336075	0.596	0.611336075	0.616	0.014	NA	6
CRELD2	0.796	0.590437309	0.592	0.590437309	0.74	0.099	NA	6
SDC1	0.792	0.505858246	0.584	0.505858246	0.589	0.003	NA	6
LMAN1	0.791	0.460372286	0.582	0.460372286	0.753	0.108	NA	6
TRIB1	0.787	0.562106926	0.574	0.562106926	0.589	0.01	NA	6
VIMP	0.78	0.565840479	0.56	0.565840479	0.89	0.227	NA	6
TXNDC5	0.777	0.535638049	0.554	0.535638049	0.562	0.005	NA	6
ERLEC1	0.777	0.46846779	0.554	0.46846779	0.795	0.152	NA	6
IGHG2	0.775	2.164210259	0.55	2.164210259	0.699	0.149	NA	6
MYDGF	0.775	0.627595575	0.55	0.627595575	0.877	0.239	NA	6
HM13	0.775	0.441673502	0.55	0.441673502	0.795	0.154	NA	6
CD79A	0.774	0.374109271	0.548	0.374109271	0.932	0.261	NA	6
JSRP1	0.769	0.810301936	0.538	0.810301936	0.548	0.008	NA	6
TXNDC15	0.769	0.346018086	0.538	0.346018086	0.63	0.057	NA	6
FAM46C	0.768	0.473027672	0.536	0.473027672	0.658	0.082	NA	6
PABPC4	0.766	0.455020623	0.532	0.455020623	0.753	0.147	NA	6
SELK	0.764	0.61577799	0.528	0.61577799	0.932	0.305	NA	6
SDF2L1	0.764	0.560508606	0.528	0.560508606	0.808	0.194	NA	6
CD59	0.762	0.556510903	0.524	0.556510903	0.616	0.064	NA	6

Table S1 Continued

Gene_marker	myAUC	Avg_diff	Power	Avg_logFC	Pct.1	Pct.2	*P*_val_adj	Cluster
LY96	0.762	0.336232806	0.524	0.336232806	0.671	0.092	NA	6
KDELR1	0.761	0.286659442	0.522	0.286659442	0.753	0.139	NA	6
PDK1	0.757	0.423721429	0.514	0.423721429	0.562	0.032	NA	6
IGHG3	0.754	3.651307515	0.508	3.651307515	0.904	0.354	NA	6
CHPF	0.754	0.431084142	0.508	0.431084142	0.534	0.017	NA	6
TPD52	0.753	0.288803467	0.506	0.288803467	0.685	0.111	NA	6
SPAG4	0.751	0.440217791	0.502	0.440217791	0.507	0.003	NA	6
ARSA	0.751	0.336735179	0.502	0.336735179	0.603	0.066	NA	6
PLPP5	0.75	0.442195772	0.5	0.442195772	0.63	0.088	NA	6
SPCS1	0.745	0.554935185	0.49	0.554935185	0.932	0.494	NA	6
ANKRD28	0.745	0.381987418	0.49	0.381987418	0.548	0.04	NA	6
SRPRB	0.744	0.32431765	0.488	0.32431765	0.589	0.067	NA	6
ZBP1	0.74	0.359807006	0.48	0.359807006	0.603	0.081	NA	6
IGHA2	0.739	1.946985611	0.478	1.946985611	0.712	0.148	NA	6
ARF4	0.737	0.275991622	0.474	0.275991622	0.767	0.18	NA	6
SELM	0.735	0.390527022	0.47	0.390527022	0.671	0.136	NA	6
GMPPB	0.734	0.319575312	0.468	0.319575312	0.562	0.063	NA	6
SEC14L1	0.731	0.293779748	0.462	0.293779748	0.521	0.039	NA	6
CECR1	0.728	0.263924544	0.456	0.263924544	0.562	0.071	NA	6
NUCB2	0.727	0.373230124	0.454	0.373230124	0.74	0.19	NA	6
DNAJB11	0.723	0.251747579	0.446	0.251747579	0.603	0.102	NA	6
SPCS2	0.721	0.561580207	0.442	0.561580207	0.918	0.469	NA	6
LMAN2	0.72	0.346821754	0.44	0.346821754	0.89	0.296	NA	6
IGKV1-12	0.719	1.279742679	0.438	1.279742679	0.452	0.013	NA	6
SEL1L	0.718	0.294303842	0.436	0.294303842	0.493	0.04	NA	6
IGKV3-20	0.716	0.4474702	0.432	0.4474702	0.589	0.114	NA	6
KDELR2	0.715	0.26920995	0.43	0.26920995	0.726	0.187	NA	6
CREB3L2	0.712	0.251392162	0.424	0.251392162	0.493	0.048	NA	6
HSPA5	0.711	0.493755595	0.422	0.493755595	0.808	0.268	NA	6
OSTC	0.711	0.25467129	0.422	0.25467129	0.822	0.254	NA	6

Table S1 Continued

Gene_marker	myAUC	Avg_diff	Power	Avg_logFC	Pct.1	Pct.2	*P*_val_adj	Cluster
SIL1	0.709	0.304907047	0.418	0.304907047	0.479	0.043	NA	6
PIM2	0.705	0.289600758	0.41	0.289600758	0.726	0.216	NA	6
SPATS2	0.704	0.317092918	0.408	0.317092918	0.452	0.031	NA	6
GAS6	0.704	0.264306953	0.408	0.264306953	0.411	0.003	NA	6
ATF4	0.704	0.251447012	0.408	0.251447012	0.699	0.191	NA	6
CPNE5	0.703	0.34041057	0.406	0.34041057	0.425	0.014	NA	6
IFI6	0.702	0.307820817	0.404	0.307820817	0.589	0.129	NA	6
STMN1	0.965	2.297809923	0.93	2.297809923	0.971	0.172	NA	7
TUBA1B	0.938	1.991089895	0.876	1.991089895	0.986	0.367	NA	7
HMGN2	0.907	1.348179172	0.814	1.348179172	1	0.665	NA	7
H2AFZ	0.905	1.385411674	0.81	1.385411674	0.986	0.474	NA	7
HMGB2	0.889	2.061483707	0.778	2.061483707	0.913	0.351	NA	7
TUBB	0.881	1.612038781	0.762	1.612038781	0.942	0.4	NA	7
PTMA	0.859	0.661983799	0.718	0.661983799	1	0.999	NA	7
HMGB1	0.851	0.995879835	0.702	0.995879835	1	0.853	NA	7
KIAA0101	0.826	1.453773822	0.652	1.453773822	0.667	0.018	NA	7
H3F3A	0.805	0.674090574	0.61	0.674090574	1	0.881	NA	7
RANBP1	0.794	0.806610228	0.588	0.806610228	0.87	0.295	NA	7
HLA-DRA	0.777	0.746101745	0.554	0.746101745	0.986	0.612	NA	7
ZWINT	0.775	1.173900193	0.55	1.173900193	0.565	0.016	NA	7
CARHSP1	0.774	0.842077465	0.548	0.842077465	0.739	0.173	NA	7
DEK	0.774	0.813493668	0.548	0.813493668	0.855	0.372	NA	7
HMGN1	0.769	0.625142124	0.538	0.625142124	0.942	0.661	NA	7
GAPDH	0.768	0.743400516	0.536	0.743400516	0.971	0.88	NA	7
CBX3	0.759	0.666868285	0.518	0.666868285	0.884	0.467	NA	7
TYMS	0.758	1.149808384	0.516	1.149808384	0.522	0.006	NA	7
ANP32B	0.758	0.813950802	0.516	0.813950802	0.797	0.36	NA	7
MCM7	0.754	0.949826267	0.508	0.949826267	0.58	0.07	NA	7
CD74	0.753	0.573761984	0.506	0.573761984	1	0.937	NA	7
DUT	0.748	0.925991613	0.496	0.925991613	0.696	0.248	NA	7

Table S1 Continued

Gene_marker	myAUC	Avg_diff	Power	Avg_logFC	Pct.1	Pct.2	*P*_val_adj	Cluster
PKM	0.747	0.69468829	0.494	0.69468829	0.899	0.431	NA	7
NASP	0.746	0.857084556	0.492	0.857084556	0.623	0.127	NA	7
MDK	0.741	1.287873859	0.482	1.287873859	0.565	0.103	NA	7
HNRNPA2B1	0.741	0.560379411	0.482	0.560379411	0.942	0.718	NA	7
IGLC6	0.739	1.457675486	0.478	1.457675486	0.609	0.17	NA	7
RAN	0.736	0.671980636	0.472	0.671980636	0.855	0.404	NA	7
PCNA	0.733	1.03961907	0.466	1.03961907	0.536	0.072	NA	7
H2AFV	0.732	0.811738345	0.464	0.811738345	0.71	0.273	NA	7
LSM5	0.729	0.730726681	0.458	0.730726681	0.739	0.291	NA	7
GSTP1	0.728	0.615119335	0.456	0.615119335	0.913	0.526	NA	7
MT-CO3	0.728	0.387814293	0.456	0.387814293	1	0.994	NA	7
HIST1H4C	0.723	1.720015824	0.446	1.720015824	0.623	0.206	NA	7
MKI67	0.721	1.17438697	0.442	1.17438697	0.449	0.008	NA	7
MS4A1	0.721	1.162497982	0.442	1.162497982	0.638	0.248	NA	7
NUCKS1	0.719	0.741354925	0.438	0.741354925	0.754	0.285	NA	7
H2AFY	0.719	0.63406173	0.438	0.63406173	0.696	0.225	NA	7
MARCKSL1	0.715	0.757168944	0.43	0.757168944	0.652	0.231	NA	7
HNRNPA1	0.713	0.371964168	0.426	0.371964168	0.971	0.91	NA	7
SIVA1	0.711	0.640679949	0.422	0.640679949	0.696	0.272	NA	7
NAP1L1	0.711	0.494014083	0.422	0.494014083	0.884	0.575	NA	7
RPA3	0.709	0.682117268	0.418	0.682117268	0.594	0.158	NA	7
CKS1B	0.708	1.049814082	0.416	1.049814082	0.464	0.052	NA	7
SUMO2	0.707	0.430121774	0.414	0.430121774	0.913	0.7	NA	7
SET	0.705	0.734089086	0.41	0.734089086	0.768	0.373	NA	7
IGLC3	0.705	0.668078306	0.41	0.668078306	0.783	0.487	NA	7
CKS2	0.704	0.935403319	0.408	0.935403319	0.478	0.074	NA	7
TCL1A	0.702	1.191313204	0.404	1.191313204	0.478	0.089	NA	7
LMNB1	0.701	0.685412585	0.402	0.685412585	0.449	0.039	NA	7
ANXA5	0.701	0.525765388	0.402	0.525765388	0.667	0.221	NA	7
LST1	1	3.713815859	1	3.713815859	1	0.078	NA	8

Table S1 Continued

Gene_marker	myAUC	Avg_diff	Power	Avg_logFC	Pct.1	Pct.2	*P*_val_adj	Cluster
AIF1	1	3.459377283	1	3.459377283	1	0.102	NA	8
CST3	1	3.094793865	1	3.094793865	1	0.03	NA	8
SERPINA1	1	2.671569858	1	2.671569858	1	0.007	NA	8
FTH1	1	2.402480627	1	2.402480627	1	0.971	NA	8
FTL	1	2.031734182	1	2.031734182	1	0.98	NA	8
CTSS	0.999	2.40944544	0.998	2.40944544	1	0.324	NA	8
FCER1G	0.998	3.269127652	0.996	3.269127652	1	0.057	NA	8
IFITM3	0.998	3.14950733	0.996	3.14950733	1	0.073	NA	8
TYROBP	0.998	3.137018335	0.996	3.137018335	1	0.094	NA	8
FCGR3A	0.995	2.390812576	0.99	2.390812576	1	0.106	NA	8
PSAP	0.995	2.102174646	0.99	2.102174646	1	0.401	NA	8
SAT1	0.993	2.380784655	0.986	2.380784655	1	0.435	NA	8
FCN1	0.99	2.860100925	0.98	2.860100925	0.98	0.01	NA	8
S100A11	0.99	2.006788942	0.98	2.006788942	1	0.411	NA	8
MS4A7	0.989	2.522592873	0.978	2.522592873	0.98	0.015	NA	8
FAM26F	0.983	2.013257254	0.966	2.013257254	0.98	0.034	NA	8
TYMP	0.98	2.11930838	0.96	2.11930838	0.98	0.1	NA	8
CD68	0.978	2.164736139	0.956	2.164736139	0.96	0.014	NA	8
S100A6	0.972	1.596372859	0.944	1.596372859	1	0.843	NA	8
CFD	0.969	2.339139765	0.938	2.339139765	0.94	0.01	NA	8
SPI1	0.965	2.080816672	0.93	2.080816672	0.94	0.033	NA	8
NPC2	0.963	1.769279211	0.926	1.769279211	0.98	0.194	NA	8
LINC01272	0.959	2.204156896	0.918	2.204156896	0.92	0.003	NA	8
S100A4	0.949	1.283928319	0.898	1.283928319	1	0.872	NA	8
IFITM2	0.948	1.586633872	0.896	1.586633872	0.98	0.59	NA	8
S100A9	0.946	2.160319107	0.892	2.160319107	0.9	0.015	NA	8
TMEM176B	0.939	1.665358491	0.878	1.665358491	0.88	0.002	NA	8
COTL1	0.928	1.086102857	0.856	1.086102857	1	0.679	NA	8
LGALS1	0.926	1.480099062	0.852	1.480099062	1	0.437	NA	8
OAZ1	0.925	0.887656228	0.85	0.887656228	1	0.901	NA	8

Table S1 Continued

Gene_marker	myAUC	Avg_diff	Power	Avg_logFC	Pct.1	Pct.2	*P*_val_adj	Cluster
FKBP1A	0.923	1.200480973	0.846	1.200480973	0.98	0.355	NA	8
STXBP2	0.917	1.734442856	0.834	1.734442856	0.9	0.168	NA	8
LILRB2	0.917	1.537165798	0.834	1.537165798	0.84	0.008	NA	8
CEBPB	0.916	1.350220261	0.832	1.350220261	0.94	0.19	NA	8
PILRA	0.909	1.3853189	0.818	1.3853189	0.82	0.003	NA	8
TKT	0.906	1.518017518	0.812	1.518017518	0.9	0.158	NA	8
EVI2B	0.893	1.279423535	0.786	1.279423535	0.98	0.44	NA	8
ASAH1	0.893	1.259915825	0.786	1.259915825	0.88	0.115	NA	8
MNDA	0.891	1.617733818	0.782	1.617733818	0.8	0.027	NA	8
HLA-DPA1	0.885	1.029041056	0.77	1.029041056	1	0.681	NA	8
LRRC25	0.879	1.49501645	0.758	1.49501645	0.76	0.002	NA	8
NEAT1	0.878	1.088630499	0.756	1.088630499	1	0.64	NA	8
LYN	0.876	1.270752793	0.752	1.270752793	0.82	0.073	NA	8
TIMP1	0.873	1.330983482	0.746	1.330983482	0.84	0.106	NA	8
CSF1R	0.869	1.366855929	0.738	1.366855929	0.74	0.003	NA	8
PECAM1	0.869	1.237113722	0.738	1.237113722	0.8	0.067	NA	8
DUSP1	0.867	0.955371745	0.734	0.955371745	1	0.734	NA	8
CLEC7A	0.859	1.313019878	0.718	1.313019878	0.72	0.001	NA	8
CARD16	0.854	1.152908908	0.708	1.152908908	0.92	0.335	NA	8
CALM2	0.854	0.911842072	0.708	0.911842072	0.98	0.714	NA	8
TCF7L2	0.853	1.220962789	0.706	1.220962789	0.72	0.012	NA	8
NAP1L1	0.852	0.951224388	0.704	0.951224388	1	0.575	NA	8
HLA-DRA	0.852	0.944127206	0.704	0.944127206	1	0.614	NA	8
C10orf54	0.85	1.252504808	0.7	1.252504808	0.86	0.242	NA	8
MT-CO1	0.85	0.522470568	0.7	0.522470568	1	0.998	NA	8
PPM1N	0.848	1.330522424	0.696	1.330522424	0.72	0.026	NA	8
SOD2	0.843	1.281383165	0.686	1.281383165	0.76	0.079	NA	8
C1orf162	0.842	1.333235891	0.684	1.333235891	0.78	0.153	NA	8
PYCARD	0.842	1.108458716	0.684	1.108458716	0.9	0.292	NA	8
TSPO	0.841	1.06884555	0.682	1.06884555	0.88	0.328	NA	8

Table S1 Continued

Gene_marker	myAUC	Avg_diff	Power	Avg_logFC	Pct.1	Pct.2	*P*_val_adj	Cluster
H3F3A	0.841	0.68404998	0.682	0.68404998	0.98	0.882	NA	8
LILRA5	0.84	1.291385234	0.68	1.291385234	0.68	0.001	NA	8
TNFSF10	0.839	1.334461738	0.678	1.334461738	0.76	0.114	NA	8
CPVL	0.839	1.17609639	0.678	1.17609639	0.68	0.002	NA	8
HCK	0.838	1.459964011	0.676	1.459964011	0.68	0.006	NA	8
CLEC12A	0.838	1.344483471	0.676	1.344483471	0.68	0.004	NA	8
FGL2	0.836	1.262466325	0.672	1.262466325	0.68	0.009	NA	8
RPS9	0.835	0.446450038	0.67	0.446450038	1	0.995	NA	8
MT2A	0.833	1.412777551	0.666	1.412777551	0.92	0.468	NA	8
ZFAND5	0.826	1.131006142	0.652	1.131006142	0.76	0.148	NA	8
VIM	0.825	0.807906408	0.65	0.807906408	0.98	0.563	NA	8
SLC11A1	0.824	1.089181269	0.648	1.089181269	0.66	0.01	NA	8
CFP	0.823	1.262863037	0.646	1.262863037	0.66	0.014	NA	8
C19orf38	0.822	1.113888738	0.644	1.113888738	0.66	0.017	NA	8
SLC31A2	0.817	1.126455526	0.634	1.126455526	0.64	0.006	NA	8
VAMP5	0.817	1.071662008	0.634	1.071662008	0.84	0.295	NA	8
CAPZA2	0.817	0.852405342	0.634	0.852405342	0.88	0.259	NA	8
DRAP1	0.817	0.766733154	0.634	0.766733154	0.96	0.475	NA	8
CYBA	0.812	0.549819073	0.624	0.549819073	1	0.926	NA	8
NCF2	0.806	1.175254129	0.612	1.175254129	0.62	0.008	NA	8
BRI3	0.806	1.114026269	0.612	1.114026269	0.68	0.07	NA	8
LYZ	0.805	1.243118387	0.61	1.243118387	0.62	0.012	NA	8
VMP1	0.804	1.070509468	0.608	1.070509468	0.78	0.197	NA	8
RHOC	0.803	1.052749458	0.606	1.052749458	0.76	0.177	NA	8
S100A8	0.802	1.624988557	0.604	1.624988557	0.62	0.021	NA	8
ARPC3	0.802	0.647134665	0.604	0.647134665	1	0.733	NA	8
LILRA1	0.8	1.204739295	0.6	1.204739295	0.6	0.001	NA	8
LILRB3	0.799	1.096368286	0.598	1.096368286	0.6	0.002	NA	8
ARRB2	0.798	0.865382246	0.596	0.865382246	0.84	0.247	NA	8
RNF130	0.796	1.016938286	0.592	1.016938286	0.66	0.063	NA	8

Table S1 Continued

Gene_marker	myAUC	Avg_diff	Power	Avg_logFC	Pct.1	Pct.2	*P*_val_adj	Cluster
OAS1	0.794	0.9523526	0.588	0.9523526	0.66	0.064	NA	8
ANXA5	0.79	0.750200383	0.58	0.750200383	0.82	0.221	NA	8
TMSB4X	0.789	0.277221408	0.578	0.277221408	1	1	NA	8
ATG3	0.788	1.084119802	0.576	1.084119802	0.68	0.118	NA	8
RNH1	0.788	0.856405451	0.576	0.856405451	0.78	0.222	NA	8
WARS	0.786	1.133973017	0.572	1.133973017	0.6	0.029	NA	8
SDCBP	0.785	0.891509301	0.57	0.891509301	0.74	0.157	NA	8
RNASET2	0.785	0.783470356	0.57	0.783470356	0.9	0.422	NA	8
NACA	0.785	0.49208631	0.57	0.49208631	1	0.909	NA	8
CASP1	0.784	0.982400087	0.568	0.982400087	0.76	0.209	NA	8
SLC25A6	0.784	0.532329483	0.568	0.532329483	1	0.84	NA	8
LILRB1	0.782	1.123027018	0.564	1.123027018	0.6	0.034	NA	8
TNFRSF1B	0.781	1.036229939	0.562	1.036229939	0.68	0.142	NA	8
BID	0.78	0.996609214	0.56	0.996609214	0.66	0.098	NA	8
MAPKAPK3	0.78	0.835943354	0.56	0.835943354	0.64	0.064	NA	8
HLA-DRB1	0.78	0.671248131	0.56	0.671248131	0.96	0.655	NA	8
ITM2B	0.78	0.662407391	0.56	0.662407391	0.98	0.829	NA	8
ARPC1B	0.78	0.521373049	0.56	0.521373049	0.98	0.702	NA	8
LILRA2	0.779	0.99304584	0.558	0.99304584	0.56	0.002	NA	8
GPBAR1	0.779	0.857675288	0.558	0.857675288	0.56	0.001	NA	8
YBX1	0.779	0.571360953	0.558	0.571360953	1	0.805	NA	8
VASP	0.778	0.918792429	0.556	0.918792429	0.76	0.213	NA	8
AP1S2	0.778	0.91133967	0.556	0.91133967	0.72	0.161	NA	8
LY96	0.777	1.031228754	0.554	1.031228754	0.64	0.095	NA	8
CSTB	0.777	0.825114307	0.554	0.825114307	0.86	0.473	NA	8
GDI2	0.777	0.761335669	0.554	0.761335669	0.82	0.404	NA	8
RGS2	0.776	1.130006501	0.552	1.130006501	0.64	0.102	NA	8
HMOX1	0.776	0.964224339	0.552	0.964224339	0.56	0.007	NA	8
ATP6V1B2	0.776	0.905392252	0.552	0.905392252	0.62	0.056	NA	8
ACTB	0.776	0.490301291	0.552	0.490301291	1	1	NA	8

Table S1 Continued

Gene_marker	myAUC	Avg_diff	Power	Avg_logFC	Pct.1	Pct.2	*P*_val_adj	Cluster
BCL2A1	0.775	1.049520206	0.55	1.049520206	0.66	0.12	NA	8
TMSB10	0.775	0.344070047	0.55	0.344070047	1	1	NA	8
GPX1	0.774	0.972518421	0.548	0.972518421	0.82	0.323	NA	8
RAB24	0.774	0.800303667	0.548	0.800303667	0.6	0.041	NA	8
LGALS9	0.773	0.85671542	0.546	0.85671542	0.68	0.121	NA	8
CD300E	0.77	1.126396129	0.54	1.126396129	0.54	0.001	NA	8
CDKN1C	0.769	1.094244225	0.538	1.094244225	0.54	0.003	NA	8
PLAUR	0.767	0.970776948	0.534	0.970776948	0.54	0.006	NA	8
LAMTOR4	0.767	0.677319874	0.534	0.677319874	0.94	0.48	NA	8
PGK1	0.765	0.738448475	0.53	0.738448475	0.86	0.343	NA	8
IFI30	0.764	0.978802169	0.528	0.978802169	0.54	0.011	NA	8
SMCO4	0.764	0.87018692	0.528	0.87018692	0.6	0.061	NA	8
DUSP6	0.763	0.868489884	0.526	0.868489884	0.56	0.028	NA	8
ZFP36	0.761	0.804047001	0.522	0.804047001	0.88	0.426	NA	8
MYL6	0.761	0.464767998	0.522	0.464767998	1	0.933	NA	8
RPS19	0.761	0.273806962	0.522	0.273806962	1	1	NA	8
C5AR1	0.76	1.067412458	0.52	1.067412458	0.52	0.001	NA	8
ANXA2	0.76	0.851478894	0.52	0.851478894	0.76	0.271	NA	8
VMO1	0.759	1.501564266	0.518	1.501564266	0.52	0.003	NA	8
CXCL16	0.759	1.079526426	0.518	1.079526426	0.52	0.004	NA	8
TMEM176A	0.759	0.918517506	0.518	0.918517506	0.52	0.002	NA	8
CD74	0.759	0.440648127	0.518	0.440648127	1	0.938	NA	8
MPEG1	0.758	0.972064658	0.516	0.972064658	0.54	0.021	NA	8
GCA	0.758	0.896023147	0.516	0.896023147	0.56	0.043	NA	8
FGR	0.755	0.818320377	0.51	0.818320377	0.7	0.184	NA	8
NOP10	0.755	0.706339608	0.51	0.706339608	0.84	0.388	NA	8
ATP6V0B	0.755	0.693411994	0.51	0.693411994	0.84	0.317	NA	8
UNC119	0.754	0.911042182	0.508	0.911042182	0.58	0.064	NA	8
RHOG	0.753	0.744443819	0.506	0.744443819	0.78	0.267	NA	8
BEST1	0.752	0.808917725	0.504	0.808917725	0.56	0.046	NA	8

Table S1 Continued

Gene_marker	myAUC	Avg_diff	Power	Avg_logFC	Pct.1	Pct.2	*P*_val_adj	Cluster
TESC	0.751	0.98310888	0.502	0.98310888	0.6	0.096	NA	8
HLA-DPB1	0.751	0.580807353	0.502	0.580807353	0.98	0.779	NA	8
MS4A4A	0.75	0.98525475	0.5	0.98525475	0.5	0.001	NA	8
LAPTM5	0.75	0.500334906	0.5	0.500334906	0.96	0.705	NA	8
PTPN6	0.749	0.734806828	0.498	0.734806828	0.76	0.283	NA	8
GRN	0.749	0.729660929	0.498	0.729660929	0.64	0.126	NA	8
IGSF6	0.748	1.022729276	0.496	1.022729276	0.5	0.004	NA	8
SLC7A7	0.747	0.882320195	0.494	0.882320195	0.54	0.042	NA	8
CPPED1	0.745	0.903456642	0.49	0.903456642	0.52	0.028	NA	8
HN1	0.745	0.666683238	0.49	0.666683238	0.78	0.249	NA	8
PRELID1	0.744	0.686972542	0.488	0.686972542	0.8	0.36	NA	8
LGALS3	0.741	0.902181761	0.482	0.902181761	0.52	0.036	NA	8
RGS19	0.74	0.753303382	0.48	0.753303382	0.72	0.228	NA	8
GABARAP	0.74	0.653525604	0.48	0.653525604	0.8	0.346	NA	8
CUX1	0.738	0.816940863	0.476	0.816940863	0.52	0.04	NA	8
MT-ATP6	0.738	0.467936472	0.476	0.467936472	1	0.901	NA	8
SIGLEC10	0.737	0.925535401	0.474	0.925535401	0.5	0.026	NA	8
FOS	0.737	0.807143685	0.474	0.807143685	0.86	0.473	NA	8
PGLS	0.737	0.692405357	0.474	0.692405357	0.68	0.182	NA	8
LAPTM4A	0.737	0.590966986	0.474	0.590966986	0.8	0.276	NA	8
ITGB2	0.736	0.527872042	0.472	0.527872042	1	0.668	NA	8
SLC2A6	0.735	0.882508114	0.47	0.882508114	0.5	0.028	NA	8
SERF2	0.734	0.355955757	0.468	0.355955757	1	0.967	NA	8
HLA-DQA2	0.733	0.725772886	0.466	0.725772886	0.82	0.383	NA	8
GNG5	0.733	0.652388586	0.466	0.652388586	0.78	0.329	NA	8
RBX1	0.733	0.57159991	0.466	0.57159991	0.84	0.342	NA	8
TUBA1B	0.732	0.615468567	0.464	0.615468567	0.8	0.373	NA	8
GMFG	0.732	0.47310163	0.464	0.47310163	0.98	0.697	NA	8
STX11	0.731	0.927816637	0.462	0.927816637	0.5	0.039	NA	8
TALDO1	0.731	0.741144033	0.462	0.741144033	0.68	0.219	NA	8

Table S1 Continued

Gene_marker	myAUC	Avg_diff	Power	Avg_logFC	Pct.1	Pct.2	*P*_val_adj	Cluster
MARCH1	0.731	0.694631154	0.462	0.694631154	0.56	0.08	NA	8
RNF149	0.731	0.655855157	0.462	0.655855157	0.68	0.193	NA	8
ZYX	0.731	0.646003736	0.462	0.646003736	0.66	0.17	NA	8
ID2	0.731	0.630673386	0.462	0.630673386	0.84	0.433	NA	8
CD48	0.731	0.578098124	0.462	0.578098124	0.86	0.532	NA	8
ARPC5	0.73	0.611100768	0.46	0.611100768	0.88	0.517	NA	8
KLF4	0.729	1.044444172	0.458	1.044444172	0.46	0.002	NA	8
APOBEC3A	0.729	0.944988123	0.458	0.944988123	0.46	0.001	NA	8
PSMA4	0.729	0.597439316	0.458	0.597439316	0.86	0.436	NA	8
THEMIS2	0.728	0.881475128	0.456	0.881475128	0.48	0.022	NA	8
RALB	0.727	0.691440221	0.454	0.691440221	0.52	0.052	NA	8
FCGR2A	0.726	0.926556969	0.452	0.926556969	0.46	0.008	NA	8
C20orf27	0.725	0.755180729	0.45	0.755180729	0.52	0.062	NA	8
CD86	0.724	0.803368936	0.448	0.803368936	0.46	0.011	NA	8
CAMK1	0.724	0.767444848	0.448	0.767444848	0.46	0.01	NA	8
SRGN	0.722	0.476090101	0.444	0.476090101	0.96	0.707	NA	8
CSTA	0.719	0.939556137	0.438	0.939556137	0.44	0.001	NA	8
NUP214	0.719	0.75176873	0.438	0.75176873	0.48	0.037	NA	8
SNAP23	0.719	0.639300847	0.438	0.639300847	0.64	0.168	NA	8
DOK2	0.719	0.575420816	0.438	0.575420816	0.82	0.325	NA	8
NAMPT	0.718	0.802692516	0.436	0.802692516	0.48	0.038	NA	8
GBP2	0.718	0.609678377	0.436	0.609678377	0.68	0.207	NA	8
FAM45A	0.718	0.592286905	0.436	0.592286905	0.56	0.097	NA	8
LTA4H	0.717	0.811727219	0.434	0.811727219	0.56	0.118	NA	8
TCIRG1	0.716	0.679347184	0.432	0.679347184	0.58	0.142	NA	8
COX5B	0.716	0.47585461	0.432	0.47585461	0.98	0.617	NA	8
SNX3	0.715	0.510592076	0.43	0.510592076	0.84	0.455	NA	8
MT-ND1	0.715	0.435973053	0.43	0.435973053	0.96	0.795	NA	8
MT-CYB	0.715	0.33810049	0.43	0.33810049	1	0.964	NA	8
SNX10	0.714	0.79911623	0.428	0.79911623	0.52	0.092	NA	8

Table S1 Continued

Gene_marker	myAUC	Avg_diff	Power	Avg_logFC	Pct.1	Pct.2	*P*_val_adj	Cluster
TBXAS1	0.714	0.789346606	0.428	0.789346606	0.46	0.031	NA	8
WSB1	0.714	0.765471525	0.428	0.765471525	0.64	0.223	NA	8
UBE2J1	0.713	0.605901203	0.426	0.605901203	0.58	0.131	NA	8
UBE2D1	0.711	0.721756106	0.422	0.721756106	0.52	0.089	NA	8
ZEB2	0.711	0.522990233	0.422	0.522990233	0.7	0.251	NA	8
PPT1	0.71	0.654342516	0.42	0.654342516	0.56	0.125	NA	8
ATP5E	0.709	0.302194048	0.418	0.302194048	1	0.976	NA	8
SERP1	0.708	0.589907086	0.416	0.589907086	0.84	0.49	NA	8
CD55	0.707	0.643552947	0.414	0.643552947	0.5	0.073	NA	8
ATP1B3	0.707	0.582156299	0.414	0.582156299	0.62	0.181	NA	8
CTSC	0.707	0.536359172	0.414	0.536359172	0.8	0.375	NA	8
ADGRE1	0.706	0.805083989	0.412	0.805083989	0.42	0.008	NA	8
HSBP1	0.706	0.758361698	0.412	0.758361698	0.56	0.153	NA	8
IFNGR1	0.706	0.63579641	0.412	0.63579641	0.5	0.07	NA	8
VAMP8	0.706	0.551241137	0.412	0.551241137	0.8	0.437	NA	8
TPI1	0.706	0.533305381	0.412	0.533305381	0.88	0.474	NA	8
MT-CO3	0.706	0.268293024	0.412	0.268293024	1	0.994	NA	8
H2AFY	0.705	0.543675616	0.41	0.543675616	0.66	0.228	NA	8
CDC42EP3	0.704	0.559954221	0.408	0.559954221	0.6	0.152	NA	8
POU2F2	0.703	0.762772322	0.406	0.762772322	0.54	0.144	NA	8
CTSD	0.703	0.606412457	0.406	0.606412457	0.66	0.245	NA	8
AP2S1	0.703	0.603823649	0.406	0.603823649	0.7	0.277	NA	8
ATP6V0D1	0.703	0.602236768	0.406	0.602236768	0.54	0.117	NA	8
BLOC1S1	0.703	0.565342665	0.406	0.565342665	0.8	0.402	NA	8
LSP1	0.703	0.468986388	0.406	0.468986388	0.94	0.675	NA	8
CMTM6	0.702	0.535207231	0.404	0.535207231	0.66	0.204	NA	8
ABI3	0.701	0.669688651	0.402	0.669688651	0.6	0.199	NA	8
TUBA1A	0.701	0.574824351	0.402	0.574824351	0.72	0.323	NA	8
HLA-DQA1	0.701	0.516028862	0.402	0.516028862	0.74	0.29	NA	8

Table S1 Continued

Gene_marker	myAUC	Avg_diff	Power	Avg_logFC	Pct.1	Pct.2	*P*_val_adj	Cluster
HLA-DRA	0.97	1.802287676	0.94	1.802287676	1	0.616	NA	9
CD74	0.963	1.523332208	0.926	1.523332208	1	0.938	NA	9
HLA-DPB1	0.938	1.290823725	0.876	1.290823725	1	0.779	NA	9
CD79A	0.895	1.377298675	0.79	1.377298675	0.971	0.267	NA	9
HLA-DPA1	0.883	1.180582682	0.766	1.180582682	0.971	0.682	NA	9
MEF2C	0.874	1.437322381	0.748	1.437322381	0.8	0.07	NA	9
HLA-DQA2	0.861	1.095393741	0.722	1.095393741	0.943	0.384	NA	9
IGKC	0.853	0.448295928	0.706	0.448295928	1	0.995	NA	9
MS4A1	0.852	1.193373882	0.704	1.193373882	0.914	0.249	NA	9
CD79B	0.851	0.993824382	0.702	0.993824382	0.971	0.31	NA	9
SUB1	0.843	1.020298531	0.686	1.020298531	0.971	0.703	NA	9
HLA-DQB1	0.834	1.135912876	0.668	1.135912876	0.857	0.283	NA	9
LAPTM5	0.831	0.950190236	0.662	0.950190236	0.943	0.706	NA	9
CLECL1	0.827	0.899005362	0.654	0.899005362	0.8	0.116	NA	9
HLA-DRB1	0.826	0.89917861	0.652	0.89917861	1	0.656	NA	9
BLK	0.823	1.017677452	0.646	1.017677452	0.714	0.057	NA	9
HLA-DMB	0.813	1.038590087	0.626	1.038590087	0.8	0.167	NA	9
CD37	0.807	0.731690298	0.614	0.731690298	1	0.727	NA	9
UBE2J1	0.802	1.035181246	0.604	1.035181246	0.714	0.131	NA	9
RPLP1	0.802	0.373029247	0.604	0.373029247	1	1	NA	9
OAZ1	0.801	0.577225947	0.602	0.577225947	1	0.901	NA	9
HLA-DQA1	0.8	0.970303743	0.6	0.970303743	0.829	0.291	NA	9
POU2F2	0.796	0.902221275	0.592	0.902221275	0.743	0.144	NA	9
CPNE5	0.794	1.405203589	0.588	1.405203589	0.6	0.016	NA	9
SNX3	0.79	0.864487117	0.58	0.864487117	0.886	0.456	NA	9
GABARAPL2	0.787	0.745667136	0.574	0.745667136	0.886	0.369	NA	9
ANXA4	0.784	0.92045491	0.568	0.92045491	0.629	0.051	NA	9
BANK1	0.777	1.145762049	0.554	1.145762049	0.629	0.085	NA	9
EEF2	0.776	0.522022166	0.552	0.522022166	1	0.892	NA	9

Table S1 Continued

Gene_marker	myAUC	Avg_diff	Power	Avg_logFC	Pct.1	Pct.2	*P*_val_adj	Cluster
GNB2L1	0.774	0.420458952	0.548	0.420458952	1	0.983	NA	9
HLA-DMA	0.771	0.815836231	0.542	0.815836231	0.8	0.299	NA	9
GNG7	0.765	1.191470838	0.53	1.191470838	0.543	0.02	NA	9
CCDC50	0.764	0.872589033	0.528	0.872589033	0.629	0.092	NA	9
CD19	0.757	0.965030779	0.514	0.965030779	0.629	0.115	NA	9
LAT2	0.751	0.960048803	0.502	0.960048803	0.571	0.075	NA	9
MT-CO3	0.75	0.410602597	0.5	0.410602597	1	0.994	NA	9
SNX2	0.748	0.927191253	0.496	0.927191253	0.6	0.108	NA	9
CAPG	0.747	0.923215012	0.494	0.923215012	0.6	0.112	NA	9
SPIB	0.746	0.920681384	0.492	0.920681384	0.571	0.075	NA	9
CALM2	0.745	0.543555456	0.49	0.543555456	1	0.714	NA	9
IRF8	0.744	0.78886217	0.488	0.78886217	0.543	0.044	NA	9
JCHAIN	0.742	0.972853389	0.484	0.972853389	0.571	0.121	NA	9
PLAC8	0.739	1.043250572	0.478	1.043250572	0.657	0.197	NA	9
MT-CO1	0.736	0.33774151	0.472	0.33774151	1	0.998	NA	9
LSP1	0.732	0.542944301	0.464	0.542944301	0.914	0.676	NA	9
TKT	0.724	0.737284673	0.448	0.737284673	0.629	0.163	NA	9
SYNGR2	0.723	0.611715231	0.446	0.611715231	0.686	0.204	NA	9
AL928768.3	0.722	2.16565284	0.444	2.16565284	0.457	0.015	NA	9
CRIP1	0.722	0.937604584	0.444	0.937604584	0.886	0.56	NA	9
CD86	0.722	0.864319519	0.444	0.864319519	0.457	0.013	NA	9
CD52	0.722	0.39674136	0.444	0.39674136	1	0.996	NA	9
RPL18A	0.722	0.283641324	0.444	0.283641324	1	1	NA	9
LY86	0.721	0.901869535	0.442	0.901869535	0.543	0.098	NA	9
ZFAND6	0.721	0.656343756	0.442	0.656343756	0.714	0.236	NA	9
GAPT	0.72	0.789682934	0.44	0.789682934	0.486	0.04	NA	9
NAP1L1	0.717	0.649465155	0.434	0.649465155	0.829	0.578	NA	9
HVCN1	0.713	0.853639775	0.426	0.853639775	0.457	0.032	NA	9
SND1	0.712	0.789407235	0.424	0.789407235	0.514	0.088	NA	9

Table S1 Continued

Gene_marker	myAUC	Avg_diff	Power	Avg_logFC	Pct.1	Pct.2	*P*_val_adj	Cluster
ACP5	0.711	0.687687046	0.422	0.687687046	0.571	0.144	NA	9
CTSH	0.71	0.643961283	0.42	0.643961283	0.514	0.08	NA	9
CIB1	0.706	0.956834571	0.412	0.956834571	0.886	0.461	NA	9
LTB	0.703	1.059066414	0.406	1.059066414	0.8	0.56	NA	9
GPX1	0.703	0.532845191	0.406	0.532845191	0.743	0.326	NA	9
UQCRH	0.702	0.505231117	0.404	0.505231117	0.857	0.527	NA	9
PDLIM1	0.701	0.558123126	0.402	0.558123126	0.571	0.141	NA	9
CYBA	0.701	0.349461349	0.402	0.349461349	1	0.926	NA	9

Table S2 Differentially expressed genes in each cell subpopulation

Gene_marker	*P*_val	Avg_logFC	Pct.1	Pct.2	*P*_val_adj	Cluster
GZMK	0	1.663182738	0.796	0.211	0	0
CCL5	7.73E-205	0.792291655	0.997	0.816	1.21E-200	0
CMC1	1.01E-151	0.972272686	0.78	0.394	1.59E-147	0
LYAR	2.95E-128	0.783510565	0.655	0.282	4.63E-124	0
CST7	3.46E-95	0.477310958	0.91	0.597	5.42E-91	0
CD8B	3.07E-92	0.695031612	0.679	0.355	4.81E-88	0
KLRG1	1.83E-91	0.693846525	0.552	0.241	2.86E-87	0
COTL1	3.02E-83	0.500765291	0.843	0.611	4.73E-79	0
SH2D1A	6.11E-81	0.663805292	0.495	0.218	9.58E-77	0
GZMM	2.27E-78	0.55693349	0.696	0.406	3.56E-74	0
GZMA	3.66E-77	0.47906139	0.84	0.544	5.74E-73	0
CD160	1.95E-75	0.69070374	0.384	0.134	3.06E-71	0
CD8A	1.70E-69	0.532511998	0.605	0.319	2.67E-65	0
TRAC	4.16E-69	0.388435975	0.943	0.74	6.52E-65	0
CTSW	1.04E-65	0.398593657	0.81	0.513	1.63E-61	0
HCST	2.65E-62	0.339944985	0.929	0.758	4.15E-58	0
CD3D	1.25E-60	0.344559655	0.955	0.772	1.97E-56	0

Table S2 Continued

Gene_marker	*P*_val	Avg_logFC	Pct.1	Pct.2	*P*_val_adj	Cluster
IL32	1.80E-55	0.323735348	0.985	0.806	2.82E-51	0
TRGC2	3.12E-52	0.570372388	0.463	0.235	4.89E-48	0
CD3E	4.16E-51	0.354669634	0.863	0.651	6.52E-47	0
CCL4	1.16E-47	0.779671224	0.589	0.382	1.82E-43	0
CD7	8.91E-46	0.408841361	0.57	0.344	1.40E-41	0
TRBC2	1.43E-42	0.347870338	0.884	0.791	2.23E-38	0
FYB	6.51E-42	0.381058016	0.66	0.466	1.02E-37	0
ZFP36L2	1.25E-41	0.321190051	0.874	0.762	1.96E-37	0
CD2	3.06E-40	0.344834621	0.743	0.548	4.80E-36	0
TRAT1	3.43E-36	0.405250483	0.399	0.218	5.38E-32	0
FYN	2.57E-31	0.388209133	0.457	0.282	4.03E-27	0
GPR171	2.61E-31	0.391666116	0.307	0.153	4.08E-27	0
ITGB2	4.68E-31	0.271214138	0.779	0.625	7.34E-27	0
PTPRC	7.16E-28	0.266517589	0.874	0.782	1.12E-23	0
CD3G	2.44E-27	0.275904375	0.659	0.475	3.82E-23	0
CD27	6.18E-27	0.377873882	0.495	0.344	9.69E-23	0
GUK1	3.14E-26	0.283921983	0.706	0.59	4.92E-22	0
PPP2R5C	1.34E-25	0.319855699	0.638	0.501	2.10E-21	0
NUCB2	1.95E-24	0.392863916	0.288	0.161	3.06E-20	0
RARRES3	3.05E-24	0.282311802	0.689	0.572	4.78E-20	0
LAG3	2.79E-23	0.333306563	0.284	0.153	4.37E-19	0
LAT	5.80E-23	0.288806499	0.619	0.474	9.09E-19	0
GIMAP4	1.63E-22	0.305962773	0.547	0.402	2.56E-18	0
PRKCH	7.18E-22	0.302303953	0.381	0.235	1.13E-17	0
MYO1F	8.76E-22	0.272023638	0.582	0.434	1.37E-17	0
CLEC2B	2.10E-19	0.300140315	0.417	0.282	3.30E-15	0
MATK	3.43E-18	0.274153428	0.26	0.146	5.37E-14	0
ARL4C	5.40E-17	0.258342628	0.401	0.275	8.47E-13	0
CD96	6.60E-16	0.251941128	0.253	0.149	1.03E-11	0
LINC00861	7.30E-16	0.256587849	0.352	0.236	1.14E-11	0

Table S2 Continued

Gene_marker	*P*_val	Avg_logFC	Pct.1	Pct.2	*P*_val_adj	Cluster
MT1X	6.63E-15	0.258190879	0.259	0.157	1.04E-10	0
STOM	2.43E-14	0.26854211	0.311	0.204	3.81E-10	0
TC2N	1.30E-13	0.260946776	0.278	0.181	2.04E-09	0
LINC00152	2.61E-13	0.284821391	0.37	0.272	4.08E-09	0
CNN2	1.01E-12	0.256740758	0.509	0.421	1.58E-08	0
DENND2D	2.19E-12	0.266205163	0.346	0.256	3.44E-08	0
ATM	3.50E-12	0.258628	0.336	0.243	5.49E-08	0
CCDC109B	1.31E-11	0.253559679	0.279	0.194	2.06E-07	0
GZMH	0	1.510234259	0.969	0.444	0	1
FGFBP2	4.52E-289	1.485499676	0.765	0.187	7.09E-285	1
NKG7	2.96E-264	1.05303263	0.999	0.785	4.63E-260	1
GZMB	7.89E-236	1.285614678	0.727	0.207	1.24E-231	1
B2M	4.44E-184	0.303392763	1	1	6.95E-180	1
CST7	5.53E-140	0.710949366	0.951	0.617	8.66E-136	1
KLRD1	1.27E-139	0.903127928	0.774	0.36	2.00E-135	1
PRF1	1.00E-135	0.89105827	0.693	0.296	1.57E-131	1
GZMA	2.72E-128	0.736590732	0.915	0.553	4.26E-124	1
CCL5	8.99E-124	0.623697673	0.998	0.835	1.41E-119	1
SH3BGRL3	1.45E-119	0.544690885	0.991	0.902	2.27E-115	1
S100A4	7.12E-117	0.602483617	0.984	0.841	1.12E-112	1
CTSW	1.49E-110	0.675718729	0.862	0.529	2.33E-106	1
ACTB	6.64E-105	0.454706549	1	1	1.04E-100	1
CD3D	7.66E-94	0.515111944	0.973	0.787	1.20E-89	1
GNLY	2.97E-87	0.682172946	0.647	0.371	4.66E-83	1
S1PR5	1.44E-84	0.705348049	0.375	0.11	2.26E-80	1
PLEK	5.86E-84	0.741920134	0.557	0.264	9.19E-80	1
ID2	5.87E-84	0.653871191	0.684	0.366	9.20E-80	1
IL32	2.07E-81	0.44979189	0.992	0.822	3.25E-77	1
HCST	1.35E-79	0.510270528	0.924	0.777	2.12E-75	1
HLA-C	4.23E-79	0.316764661	1	0.998	6.62E-75	1

Table S2 Continued

Gene_marker	*P*_val	Avg_logFC	Pct.1	Pct.2	*P*_val_adj	Cluster
LAIR2	1.95E-76	0.696689934	0.288	0.068	3.06E-72	1
ADGRG1	1.64E-73	0.662468629	0.3	0.079	2.57E-69	1
RAP1B	4.09E-71	0.594501307	0.705	0.469	6.41E-67	1
CX3CR1	1.02E-68	0.628501433	0.26	0.063	1.60E-64	1
S100A6	2.19E-68	0.406468499	0.952	0.814	3.43E-64	1
FCRL6	3.79E-66	0.618644711	0.337	0.108	5.94E-62	1
CD8A	6.50E-65	0.615468922	0.62	0.345	1.02E-60	1
PFN1	2.16E-62	0.312470139	0.999	0.991	3.39E-58	1
TBX21	2.19E-62	0.586491814	0.313	0.099	3.44E-58	1
LITAF	1.89E-61	0.550116009	0.716	0.511	2.97E-57	1
SPON2	3.45E-59	0.654701489	0.342	0.125	5.40E-55	1
AC092580.4	7.18E-56	0.564226619	0.618	0.373	1.12E-51	1
C12orf75	6.61E-53	0.575935314	0.6	0.384	1.04E-48	1
CFL1	8.26E-53	0.328535831	0.978	0.956	1.30E-48	1
CD99	1.34E-52	0.437769864	0.829	0.673	2.10E-48	1
CD8B	1.75E-51	0.511671535	0.647	0.399	2.74E-47	1
ARPC2	8.70E-51	0.420652206	0.887	0.78	1.36E-46	1
CD3G	1.08E-47	0.502129033	0.681	0.488	1.69E-43	1
GZMM	3.16E-47	0.453369186	0.677	0.442	4.96E-43	1
ZEB2	3.52E-47	0.623468094	0.418	0.209	5.51E-43	1
SRGN	6.62E-45	0.36324483	0.831	0.675	1.04E-40	1
ITGB2	2.78E-44	0.418807369	0.801	0.635	4.36E-40	1
CALM1	5.32E-43	0.372636323	0.856	0.727	8.34E-39	1
ANXA1	8.36E-43	0.447529482	0.644	0.435	1.31E-38	1
HLA-E	1.75E-41	0.290645683	0.963	0.93	2.74E-37	1
CD3E	2.22E-40	0.356234712	0.854	0.676	3.48E-36	1
CCL4	3.69E-40	0.268213033	0.62	0.394	5.79E-36	1
CD2	1.48E-39	0.391746637	0.752	0.566	2.33E-35	1
EMP3	7.31E-38	0.377226858	0.803	0.658	1.15E-33	1
MYL12A	5.14E-37	0.299101414	0.946	0.88	8.05E-33	1

Table S2 Continued

Gene_marker	*P*_val	Avg_logFC	Pct.1	Pct.2	*P*_val_adj	Cluster
ACTG1	3.04E-35	0.311916072	0.942	0.905	4.76E-31	1
BIN2	5.11E-34	0.430621826	0.569	0.395	8.00E-30	1
TRAC	5.61E-34	0.289308701	0.936	0.763	8.80E-30	1
LSP1	8.29E-34	0.3406872	0.785	0.647	1.30E-29	1
MYO1F	1.52E-32	0.409722045	0.615	0.439	2.38E-28	1
LGALS1	4.36E-32	0.439668558	0.578	0.404	6.84E-28	1
EFHD2	5.30E-32	0.482308651	0.281	0.131	8.30E-28	1
CTSC	7.90E-32	0.46926035	0.504	0.344	1.24E-27	1
CLEC2D	1.22E-30	0.396091928	0.636	0.48	1.92E-26	1
APMAP	9.37E-30	0.449172187	0.405	0.245	1.47E-25	1
TRGC2	1.20E-29	0.440859585	0.446	0.264	1.87E-25	1
C9orf142	2.26E-29	0.398479932	0.546	0.389	3.55E-25	1
CHST12	9.66E-29	0.43992547	0.407	0.248	1.51E-24	1
RARRES3	6.36E-28	0.341407664	0.712	0.578	9.97E-24	1
ABI3	1.23E-26	0.438392425	0.316	0.171	1.93E-22	1
CLIC3	2.42E-25	0.448915835	0.304	0.162	3.79E-21	1
SAMD3	5.68E-25	0.427900147	0.358	0.213	8.90E-21	1
RAC2	4.31E-23	0.30531938	0.733	0.64	6.76E-19	1
SPN	4.40E-23	0.42635224	0.359	0.222	6.90E-19	1
GIMAP7	1.29E-22	0.312579019	0.652	0.505	2.03E-18	1
PYHIN1	2.39E-22	0.345240402	0.481	0.334	3.75E-18	1
MT2A	3.46E-22	0.36307764	0.58	0.442	5.43E-18	1
IL2RG	7.84E-22	0.286605629	0.735	0.649	1.23E-17	1
PPP1CA	1.82E-21	0.297460345	0.697	0.6	2.85E-17	1
PPP1R18	1.95E-21	0.362945369	0.496	0.374	3.06E-17	1
FGR	6.48E-21	0.361399684	0.29	0.161	1.02E-16	1
CAP1	1.05E-20	0.335089402	0.59	0.493	1.64E-16	1
LAG3	2.20E-20	0.365216697	0.292	0.165	3.44E-16	1
BATF	2.71E-20	0.385519347	0.305	0.182	4.24E-16	1
MYL12B	7.00E-19	0.255398051	0.786	0.714	1.10E-14	1

Table S2 Continued

Gene_marker	*P*_val	Avg_logFC	Pct.1	Pct.2	*P*_val_adj	Cluster
CLIC1	8.00E-19	0.267595512	0.735	0.655	1.25E-14	1
FAM49B	3.02E-18	0.317768639	0.552	0.452	4.74E-14	1
GIMAP4	8.41E-18	0.303572633	0.54	0.419	1.32E-13	1
LY6E	1.00E-17	0.26816915	0.69	0.602	1.57E-13	1
DSTN	2.57E-17	0.346963432	0.395	0.278	4.03E-13	1
CCND3	3.81E-17	0.29830062	0.533	0.424	5.97E-13	1
PATL2	3.06E-16	0.352714478	0.275	0.166	4.80E-12	1
SLC9A3R1	2.03E-15	0.32917303	0.437	0.335	3.18E-11	1
ARL6IP1	2.32E-15	0.267699726	0.513	0.429	3.63E-11	1
KLRG1	7.05E-15	0.284817849	0.436	0.308	1.10E-10	1
CD48	9.49E-15	0.269099648	0.604	0.516	1.49E-10	1
ARPC5L	1.68E-14	0.33690671	0.396	0.293	2.64E-10	1
FKBP11	2.86E-14	0.28541183	0.352	0.242	4.48E-10	1
YWHAQ	5.31E-14	0.291282964	0.534	0.467	8.33E-10	1
LCP1	5.50E-14	0.286959033	0.518	0.434	8.62E-10	1
OSTF1	7.26E-14	0.316451766	0.389	0.293	1.14E-09	1
HOPX	9.02E-14	0.286385648	0.318	0.209	1.41E-09	1
XBP1	1.27E-13	0.277137769	0.407	0.298	1.99E-09	1
STOM	1.31E-13	0.286538685	0.316	0.213	2.05E-09	1
ARPC5	1.62E-13	0.28102033	0.571	0.507	2.53E-09	1
MATK	1.95E-13	0.295494043	0.256	0.159	3.06E-09	1
VAMP5	4.19E-13	0.311826054	0.373	0.281	6.57E-09	1
RNF167	5.31E-13	0.292356045	0.412	0.315	8.33E-09	1
TPST2	9.25E-13	0.30313541	0.323	0.227	1.45E-08	1
ARL6IP5	9.38E-13	0.25278143	0.632	0.582	1.47E-08	1
LBH	1.20E-12	0.284177622	0.414	0.323	1.89E-08	1
FLNA	1.31E-12	0.286177262	0.363	0.268	2.05E-08	1
DBI	1.64E-12	0.270347676	0.535	0.463	2.57E-08	1
CLEC2B	2.52E-12	0.308722612	0.392	0.303	3.94E-08	1
MBP	1.40E-11	0.259774827	0.359	0.27	2.20E-07	1

Table S2 Continued

Gene_marker	*P*_val	Avg_logFC	Pct.1	Pct.2	*P*_val_adj	Cluster
LINC00152	2.49E-11	0.278805237	0.37	0.282	3.91E-07	1
MYO1G	4.49E-11	0.264962519	0.409	0.326	7.03E-07	1
CD247	6.15E-11	0.255839601	0.408	0.319	9.65E-07	1
CYTH4	7.28E-11	0.272416692	0.276	0.191	1.14E-06	1
PTP4A2	1.15E-10	0.277677644	0.407	0.335	1.80E-06	1
SUPT4H1	2.02E-10	0.274555693	0.336	0.259	3.16E-06	1
GRAP2	2.45E-10	0.270645182	0.252	0.169	3.84E-06	1
RORA	2.51E-10	0.281195376	0.327	0.244	3.93E-06	1
APOBEC3G	2.93E-10	0.289023833	0.359	0.282	4.59E-06	1
PLAC8	3.50E-10	0.260161766	0.266	0.181	5.48E-06	1
SCP2	4.16E-10	0.257006692	0.438	0.375	6.52E-06	1
RAB29	6.45E-10	0.26034471	0.255	0.177	1.01E-05	1
ARRB2	4.76E-09	0.268823176	0.31	0.237	7.46E-05	1
C5orf56	4.37E-08	0.267615797	0.299	0.23	0.000685569	1
TRBC1	5.01E-08	0.259964517	0.531	0.468	0.000785336	1
KLRB1	0.000315036	0.378719672	0.262	0.218	1	1
C1orf56	0.008639527	0.258011862	0.3	0.277	1	1
LTB	2.88E-215	1.262633582	0.926	0.474	4.51E-211	2
EEF1A1	2.19E-145	0.346447159	1	1	3.43E-141	2
IL7R	1.31E-142	1.01777479	0.733	0.281	2.06E-138	2
RPL10	3.98E-141	0.346185404	1	1	6.24E-137	2
RPLP1	1.45E-136	0.400891514	1	1	2.28E-132	2
RPL13	1.76E-134	0.319839963	1	1	2.76E-130	2
RPL32	6.83E-126	0.33152658	1	1	1.07E-121	2
RPS25	2.22E-123	0.386838669	1	0.998	3.49E-119	2
RPL34	2.53E-119	0.357749148	1	1	3.97E-115	2
MAL	9.11E-119	0.788375569	0.279	0.031	1.43E-114	2
RPS6	4.12E-117	0.32181637	1	1	6.46E-113	2
RPL21	1.85E-109	0.299862331	1	1	2.91E-105	2
LDHB	1.19E-106	0.813717458	0.832	0.53	1.86E-102	2

Table S2 Continued

Gene_marker	*P*_val	Avg_logFC	Pct.1	Pct.2	*P*_val_adj	Cluster
RPS8	3.92E-106	0.339865853	1	0.999	6.14E-102	2
RPS18	6.88E-101	0.28150499	1	1	1.08E-96	2
RPL36	9.09E-101	0.320563065	1	1	1.42E-96	2
TPT1	5.47E-99	0.4321877	0.996	0.977	8.58E-95	2
RPS27A	3.08E-98	0.299561708	1	1	4.84E-94	2
FXYD5	1.95E-93	0.7250378	0.825	0.555	3.06E-89	2
RPS3A	2.36E-87	0.304044778	1	1	3.70E-83	2
RPL18A	1.46E-86	0.277247751	1	1	2.29E-82	2
RPL39	3.28E-86	0.27774111	1	1	5.14E-82	2
LEF1	1.42E-83	0.721456439	0.321	0.072	2.22E-79	2
RPL11	1.42E-83	0.259302292	1	1	2.23E-79	2
RPL36A	1.26E-82	0.374102656	0.999	0.986	1.98E-78	2
RPS12	1.54E-82	0.286011451	1	1	2.41E-78	2
SELL	2.60E-81	0.786729762	0.523	0.199	4.07E-77	2
ITGB1	1.28E-80	0.761012071	0.605	0.281	2.00E-76	2
RPL9	2.11E-76	0.321438143	1	0.996	3.31E-72	2
RPL12	1.09E-72	0.275941409	0.999	0.999	1.71E-68	2
RPL35A	2.85E-70	0.255497185	1	1	4.47E-66	2
RPS13	1.47E-69	0.302885047	0.999	0.997	2.30E-65	2
RPL31	3.51E-69	0.272156715	1	0.999	5.49E-65	2
RPS4X	1.83E-66	0.259570207	1	0.999	2.87E-62	2
RPL7	2.69E-64	0.281083412	1	0.999	4.22E-60	2
RPL30	1.43E-62	0.264564495	1	0.998	2.24E-58	2
TNFRSF25	3.25E-62	0.577650835	0.294	0.08	5.10E-58	2
RPL10A	4.99E-62	0.290855477	1	0.992	7.83E-58	2
RPL4	2.57E-60	0.321465258	0.981	0.977	4.02E-56	2
TMEM123	6.59E-60	0.610694636	0.546	0.259	1.03E-55	2
RPL8	2.79E-55	0.275566392	0.999	0.992	4.38E-51	2
KLRB1	2.43E-54	0.861815174	0.431	0.179	3.81E-50	2
RPL6	9.21E-53	0.250558711	1	0.997	1.44E-48	2

Table S2 Continued

Gene_marker	*P*_val	Avg_logFC	Pct.1	Pct.2	*P*_val_adj	Cluster
EIF3E	1.24E-52	0.434338504	0.91	0.773	1.95E-48	2
GPR183	4.06E-43	0.532258103	0.311	0.116	6.36E-39	2
RPL22	4.06E-40	0.277875585	0.99	0.968	6.37E-36	2
HINT1	5.81E-40	0.379461217	0.881	0.778	9.11E-36	2
GSTK1	2.59E-39	0.450707259	0.739	0.542	4.06E-35	2
BIRC3	3.47E-38	0.545228207	0.302	0.121	5.44E-34	2
RPLP0	1.51E-37	0.29559465	0.981	0.965	2.36E-33	2
SNHG25	4.36E-37	0.442382149	0.766	0.621	6.83E-33	2
CORO1B	1.94E-36	0.603431315	0.404	0.208	3.04E-32	2
GATA3	1.06E-32	0.466134535	0.265	0.106	1.66E-28	2
PABPC1	1.07E-31	0.284868051	0.948	0.899	1.68E-27	2
PIK3IP1	5.62E-30	0.472942649	0.39	0.207	8.80E-26	2
S100A11	2.26E-29	0.365317694	0.587	0.378	3.54E-25	2
NOSIP	1.32E-28	0.537037679	0.44	0.27	2.07E-24	2
TRAT1	1.76E-28	0.401255649	0.434	0.236	2.75E-24	2
TRADD	2.41E-27	0.453524438	0.36	0.194	3.78E-23	2
EEF2	3.14E-27	0.264093201	0.934	0.883	4.92E-23	2
JUNB	4.06E-26	0.544400181	0.8	0.715	6.36E-22	2
CD82	7.20E-25	0.367119619	0.274	0.128	1.13E-20	2
TCF7	2.45E-23	0.388684173	0.334	0.18	3.84E-19	2
SARAF	2.91E-23	0.331337141	0.796	0.674	4.57E-19	2
NDFIP1	2.96E-23	0.399854168	0.412	0.249	4.64E-19	2
CRIP1	3.08E-22	0.416879014	0.675	0.535	4.82E-18	2
ITM2A	1.59E-21	0.532890673	0.48	0.324	2.49E-17	2
PLP2	1.69E-21	0.420811315	0.354	0.207	2.65E-17	2
GLTSCR2	2.79E-20	0.258384393	0.884	0.793	4.37E-16	2
C1orf162	5.50E-20	0.356962598	0.267	0.135	8.62E-16	2
LEPROTL1	1.78E-19	0.365324806	0.422	0.268	2.79E-15	2
RPS4Y1	1.04E-18	0.29966858	0.859	0.825	1.63E-14	2
HSPA8	1.52E-18	0.311713677	0.702	0.576	2.38E-14	2

Table S2 Continued

Gene_marker	*P*_val	Avg_logFC	Pct.1	Pct.2	*P*_val_adj	Cluster
LIMS1	3.26E-18	0.359770069	0.262	0.141	5.11E-14	2
CYLD	6.33E-18	0.323559757	0.283	0.153	9.93E-14	2
EEF1B2	6.58E-18	0.257721603	0.863	0.788	1.03E-13	2
S100A10	9.47E-16	0.286797044	0.708	0.575	1.48E-11	2
VIM	2.06E-15	0.287442664	0.672	0.543	3.23E-11	2
TRBC1	4.17E-15	0.421404073	0.591	0.456	6.53E-11	2
CD44	9.73E-14	0.298727822	0.528	0.396	1.52E-09	2
EVI2B	2.45E-13	0.299530656	0.543	0.423	3.84E-09	2
TNFAIP3	4.45E-13	0.308743337	0.297	0.185	6.98E-09	2
NBEAL1	9.12E-12	0.262139978	0.551	0.432	1.43E-07	2
FYB	1.38E-11	0.279472837	0.625	0.502	2.17E-07	2
SNHG8	1.67E-11	0.250141841	0.379	0.262	2.62E-07	2
PBXIP1	1.30E-10	0.251395413	0.302	0.199	2.03E-06	2
NDUFS5	5.70E-10	0.251554564	0.521	0.42	8.94E-06	2
SPOCK2	8.03E-10	0.293095125	0.333	0.236	1.26E-05	2
PPP1R2	6.98E-09	0.254685517	0.444	0.35	0.000109416	2
RGS1	4.20E-06	0.291544858	0.262	0.193	0.065864362	2
IGLC6	0	2.374780226	0.88	0.066	0	3
MS4A1	0	2.241975953	0.951	0.144	0	3
IGLC7	0	2.212569393	0.909	0.122	0	3
CD79A	0	2.108262151	0.956	0.164	0	3
CCND1	0	2.014724192	0.874	0.101	0	3
CD79B	0	1.996487639	0.953	0.215	0	3
VPREB3	0	1.935116145	0.852	0.09	0	3
EGR1	0	1.78657996	0.796	0.109	0	3
IGLC3	0	1.661713956	0.996	0.412	0	3
SMIM14	0	1.644623051	0.749	0.097	0	3
CD24	0	1.474685646	0.638	0.049	0	3
IGLC2	3.70E-292	1.568590115	1	0.939	5.79E-288	3
BTG2	1.43E-282	1.72931512	0.882	0.227	2.23E-278	3

Table S2 Continued

Gene_marker	*P*_val	Avg_logFC	Pct.1	Pct.2	*P*_val_adj	Cluster
IGHM	9.18E-268	1.031200992	0.951	0.378	1.44E-263	3
RP5-887A10.1	1.21E-262	1.408687628	0.506	0.035	1.90E-258	3
CD74	6.62E-261	1.326480013	1	0.928	1.04E-256	3
HLA-DRA	3.63E-257	1.679595904	0.998	0.558	5.69E-253	3
ARHGAP24	4.64E-257	1.171619277	0.503	0.036	7.28E-253	3
TNFRSF13C	9.92E-254	1.307013475	0.579	0.063	1.56E-249	3
FAM129C	1.69E-250	1.186769319	0.506	0.04	2.66E-246	3
CD9	3.70E-245	1.245715836	0.512	0.044	5.80E-241	3
GNG11	3.89E-242	1.181735457	0.497	0.04	6.11E-238	3
FCRL2	1.32E-237	1.183046235	0.514	0.047	2.07E-233	3
LY86	2.58E-235	1.095702368	0.488	0.04	4.05E-231	3
TCL1A	1.01E-229	1.13480931	0.47	0.036	1.58E-225	3
IGHD	2.60E-226	1.126875383	0.49	0.044	4.08E-222	3
SOX11	1.65E-224	1.289088914	0.421	0.026	2.59E-220	3
RP11-231C14.7	5.06E-221	1.124857811	0.475	0.042	7.93E-217	3
CD69	1.36E-220	1.595866207	0.958	0.55	2.14E-216	3
CLECL1	4.66E-211	1.141155999	0.514	0.06	7.31E-207	3
MARCKS	5.02E-199	1.017590781	0.426	0.037	7.87E-195	3
RHOB	1.93E-188	1.260208226	0.479	0.06	3.03E-184	3
CD19	3.49E-186	1.085021295	0.481	0.061	5.48E-182	3
IER2	6.26E-184	1.481617247	0.925	0.522	9.81E-180	3
RALGPS2	3.91E-180	0.992476462	0.41	0.04	6.13E-176	3
MARCKSL1	1.40E-171	1.185481393	0.672	0.169	2.19E-167	3
H3F3B	1.93E-171	0.978617323	0.991	0.953	3.02E-167	3
FADS3	8.76E-169	0.995015975	0.413	0.046	1.37E-164	3
MDK	9.55E-169	1.241618879	0.448	0.057	1.50E-164	3
BANK1	6.38E-159	0.913235055	0.39	0.042	1.00E-154	3
TCF4	3.87E-158	0.917390766	0.383	0.041	6.07E-154	3
SPIB	5.94E-157	0.954272165	0.362	0.035	9.31E-153	3
CD72	2.31E-154	0.89608058	0.383	0.042	3.62E-150	3

Table S2 Continued

Gene_marker	*P*_val	Avg_logFC	Pct.1	Pct.2	*P*_val_adj	Cluster
HLA-DMB	9.23E-151	1.018478384	0.548	0.112	1.45E-146	3
ALOX5	9.69E-150	0.854002085	0.348	0.033	1.52E-145	3
CD37	1.28E-148	0.974961817	0.942	0.696	2.00E-144	3
BLNK	5.17E-148	0.855023528	0.359	0.038	8.11E-144	3
MT-ND4	3.59E-144	0.639351731	0.998	0.984	5.63E-140	3
FCRLA	7.64E-144	0.792631652	0.324	0.028	1.20E-139	3
PHACTR1	5.30E-143	0.921520857	0.342	0.035	8.31E-139	3
AP3B1	1.78E-142	0.95736193	0.452	0.075	2.78E-138	3
ZFP36L1	9.77E-141	1.200476064	0.821	0.363	1.53E-136	3
MARCH1	2.32E-140	0.87025507	0.362	0.042	3.64E-136	3
RPS11	1.14E-138	0.596069729	0.995	0.981	1.78E-134	3
CH17-373J23.1	1.24E-138	1.250636827	0.645	0.188	1.94E-134	3
SERPINF1	2.44E-138	0.734730006	0.277	0.018	3.83E-134	3
CD83	9.88E-135	0.842175517	0.317	0.031	1.55E-130	3
LINC00926	1.67E-133	0.841162823	0.337	0.038	2.62E-129	3
MT-ND3	1.16E-132	0.626965758	1	0.978	1.82E-128	3
KLF6	1.37E-132	1.060020331	0.922	0.646	2.15E-128	3
ITM2C	6.59E-131	1.012729233	0.532	0.122	1.03E-126	3
HRK	1.29E-130	0.72434187	0.262	0.018	2.02E-126	3
RGS12	1.62E-130	0.860393442	0.293	0.026	2.53E-126	3
SWAP70	4.70E-127	0.745476477	0.293	0.027	7.36E-123	3
JUN	8.83E-125	1.07909617	0.951	0.731	1.38E-120	3
CCDC191	1.86E-124	0.729494765	0.273	0.023	2.92E-120	3
BTK	8.47E-121	0.802235806	0.315	0.038	1.33E-116	3
NCF1	4.67E-118	0.91275865	0.556	0.151	7.33E-114	3
BLK	2.82E-117	0.693664079	0.282	0.028	4.42E-113	3
HLA-DMA	3.65E-117	1.018449331	0.683	0.243	5.72E-113	3
POU2F2	2.85E-116	0.930241173	0.461	0.1	4.47E-112	3
LHPP	6.28E-114	0.805268246	0.364	0.058	9.85E-110	3
ORAI2	7.87E-112	0.902036774	0.448	0.101	1.23E-107	3

Table S2 Continued

Gene_marker	*P*_val	Avg_logFC	Pct.1	Pct.2	*P*_val_adj	Cluster
SMARCB1	9.04E-109	0.912583928	0.519	0.142	1.42E-104	3
LINC01480	7.61E-107	0.816481497	0.288	0.035	1.19E-102	3
HLA-DOB	6.65E-102	0.644124763	0.253	0.027	1.04E-97	3
FOS	6.75E-102	1.154297491	0.787	0.428	1.06E-97	3
STMN1	5.65E-100	0.87885143	0.497	0.136	8.85E-96	3
ACADM	5.32E-96	0.86258309	0.479	0.13	8.33E-92	3
RNASE6	5.56E-96	0.725726223	0.282	0.039	8.72E-92	3
CCDC50	1.99E-94	0.695994506	0.333	0.059	3.13E-90	3
DUSP2	4.99E-93	1.174507654	0.812	0.528	7.83E-89	3
RP11-386I14.4	2.85E-91	1.08388621	0.661	0.285	4.46E-87	3
RPS5	2.18E-89	0.443319731	0.996	0.985	3.42E-85	3
PDLIM1	2.12E-86	0.748533055	0.412	0.102	3.32E-82	3
HLA-DQA2	4.07E-86	0.806894695	0.734	0.334	6.38E-82	3
GRN	2.45E-81	0.794632284	0.381	0.093	3.84E-77	3
MALAT1	4.29E-81	0.334287	1	1	6.72E-77	3
TRAF4	5.19E-80	0.660886425	0.251	0.038	8.14E-76	3
STX7	2.18E-79	0.740391217	0.39	0.099	3.42E-75	3
RPS23	2.55E-79	0.343238572	1	1	4.00E-75	3
RNF130	1.34E-78	0.586389162	0.26	0.041	2.10E-74	3
SPINT2	4.67E-77	0.707017881	0.301	0.06	7.32E-73	3
LGALS9	3.52E-76	0.709412448	0.362	0.091	5.51E-72	3
CXXC5	4.36E-76	0.623978939	0.262	0.044	6.84E-72	3
LAT2	1.84E-74	0.630499198	0.271	0.049	2.88E-70	3
CYB561A3	6.14E-74	0.747994897	0.37	0.095	9.62E-70	3
FCMR	2.37E-73	0.751508811	0.545	0.206	3.71E-69	3
MZB1	3.87E-71	0.498377001	0.468	0.149	6.07E-67	3
DUSP1	1.05E-69	0.72726576	0.905	0.711	1.65E-65	3
DERL3	3.10E-68	0.578051338	0.27	0.051	4.86E-64	3
QRSL1	4.11E-68	0.655736539	0.295	0.065	6.44E-64	3
GSTP1	1.87E-67	0.777266861	0.778	0.493	2.93E-63	3

Table S2 Continued

Gene_marker	*P*_val	Avg_logFC	Pct.1	Pct.2	*P*_val_adj	Cluster
TMEM107	1.70E-65	0.790995477	0.47	0.171	2.66E-61	3
DUSP22	3.47E-65	0.651500028	0.332	0.085	5.44E-61	3
FOSB	1.37E-64	0.841337982	0.59	0.272	2.15E-60	3
CCR7	2.54E-64	0.696465846	0.388	0.114	3.99E-60	3
HLA-DQA1	1.33E-63	0.735472252	0.587	0.249	2.09E-59	3
SEC62	6.33E-63	0.732654752	0.743	0.437	9.93E-59	3
TSC22D3	6.49E-62	0.556829576	0.929	0.762	1.02E-57	3
PTMA	5.62E-61	0.354679733	1	0.999	8.80E-57	3
HERPUD1	6.45E-61	0.946528425	0.599	0.304	1.01E-56	3
MT-ND1	2.29E-59	0.563116939	0.938	0.775	3.59E-55	3
CXCR4	2.50E-59	0.738836566	0.803	0.568	3.92E-55	3
MT-CO2	1.54E-57	0.346669126	0.998	0.997	2.42E-53	3
TMEM154	2.61E-55	0.580151719	0.297	0.077	4.09E-51	3
PNN	1.26E-53	0.704067184	0.587	0.291	1.98E-49	3
TMEM243	1.41E-53	0.667694027	0.47	0.194	2.22E-49	3
ZFP36	3.46E-53	0.818349462	0.656	0.396	5.42E-49	3
TUBB	4.51E-53	0.629339493	0.658	0.37	7.06E-49	3
PSAP	5.89E-53	0.613366204	0.667	0.367	9.23E-49	3
DDAH2	7.88E-52	0.56943864	0.277	0.071	1.23E-47	3
HIST1H4C	1.11E-50	0.589915675	0.439	0.177	1.73E-46	3
SNHG7	2.25E-50	0.651185643	0.432	0.171	3.53E-46	3
NSG1	2.26E-49	0.620435802	0.257	0.065	3.54E-45	3
RRAS2	1.21E-46	0.51860109	0.306	0.092	1.89E-42	3
HLA-DPA1	9.36E-46	0.496435966	0.852	0.658	1.47E-41	3
FOXP1	5.96E-44	0.598432407	0.423	0.176	9.34E-40	3
DRAM2	1.01E-43	0.557528164	0.395	0.158	1.59E-39	3
KTN1	1.39E-43	0.626147771	0.552	0.288	2.17E-39	3
HLA-DQB1	2.48E-42	0.615271005	0.514	0.252	3.90E-38	3
RPS16	1.83E-41	0.250939129	1	0.998	2.87E-37	3
SYPL1	1.92E-41	0.514121765	0.342	0.122	3.01E-37	3

Table S2 Continued

Gene_marker	*P*_val	Avg_logFC	Pct.1	Pct.2	*P*_val_adj	Cluster
IGHG2	3.14E-41	0.388537312	0.35	0.128	4.93E-37	3
H3F3A	9.31E-41	0.397447889	0.945	0.873	1.46E-36	3
CHI3L2	1.22E-40	0.556643841	0.31	0.105	1.91E-36	3
TUBA1A	1.34E-40	0.693446693	0.55	0.293	2.10E-36	3
RIC3	2.33E-40	0.465504596	0.259	0.076	3.66E-36	3
RP11-347P5.1	2.59E-40	0.628105185	0.687	0.443	4.06E-36	3
HLA-DRB1	1.91E-38	0.526381489	0.827	0.632	3.00E-34	3
MSI2	2.37E-38	0.466519721	0.266	0.084	3.71E-34	3
TAF7	9.50E-36	0.52591954	0.495	0.256	1.49E-31	3
PDE4B	4.79E-35	0.460416459	0.251	0.08	7.52E-31	3
PPP1R15A	7.56E-34	0.53663606	0.658	0.446	1.19E-29	3
PRDM2	1.23E-33	0.513005296	0.324	0.13	1.93E-29	3
FCGRT	2.06E-32	0.499268449	0.288	0.108	3.23E-28	3
PPM1K	1.48E-31	0.530306983	0.348	0.153	2.33E-27	3
GGA1	1.68E-30	0.481646878	0.435	0.215	2.64E-26	3
JMJD1C	2.66E-30	0.468236581	0.301	0.12	4.17E-26	3
RP11-160E2.6	3.04E-27	0.453733201	0.432	0.22	4.77E-23	3
PMAIP1	4.96E-27	0.402883373	0.271	0.107	7.77E-23	3
RTN4	8.79E-27	0.51318619	0.466	0.266	1.38E-22	3
TRAF5	9.90E-27	0.422056054	0.257	0.101	1.55E-22	3
ZBTB20	1.47E-26	0.427882881	0.253	0.098	2.30E-22	3
MT-ND5	1.63E-26	0.357378979	0.905	0.807	2.56E-22	3
APEX1	2.67E-26	0.468284654	0.333	0.157	4.19E-22	3
WASF2	1.22E-25	0.478909038	0.466	0.271	1.91E-21	3
RCSD1	2.84E-25	0.462848315	0.459	0.262	4.45E-21	3
RNASET2	1.69E-24	0.423644591	0.603	0.4	2.64E-20	3
JUND	2.10E-24	0.500364308	0.408	0.226	3.30E-20	3
TIA1	3.10E-24	0.419253842	0.273	0.116	4.86E-20	3
RP5-1171I10.5	4.71E-24	0.533672728	0.468	0.279	7.39E-20	3
MT-ND2	1.60E-23	0.25204314	0.987	0.979	2.50E-19	3

Table S2 Continued

Gene_marker	*P*_val	Avg_logFC	Pct.1	Pct.2	*P*_val_adj	Cluster
PSMA3-AS1	1.66E-23	0.429196446	0.505	0.306	2.61E-19	3
CHMP1B	3.40E-23	0.442876501	0.377	0.2	5.34E-19	3
MCL1	7.83E-23	0.498760752	0.601	0.435	1.23E-18	3
MPG	1.59E-22	0.426097602	0.33	0.161	2.49E-18	3
BTG1	2.14E-22	0.302627728	0.942	0.904	3.36E-18	3
PRKCB	5.64E-22	0.422613964	0.454	0.268	8.84E-18	3
LAPTM4A	7.65E-22	0.4572657	0.435	0.258	1.20E-17	3
PHF14	2.20E-21	0.398307821	0.337	0.171	3.45E-17	3
PNISR	2.45E-21	0.375515796	0.78	0.622	3.84E-17	3
GTF2I	3.24E-21	0.374042003	0.266	0.119	5.07E-17	3
HNRNPA0	3.26E-20	0.3805986	0.672	0.511	5.11E-16	3
TLK1	1.44E-19	0.39771236	0.271	0.13	2.25E-15	3
CNPY3	2.86E-18	0.382633136	0.361	0.202	4.48E-14	3
CHD9	1.01E-17	0.372255423	0.282	0.14	1.59E-13	3
HPCAL1	6.46E-17	0.331116239	0.271	0.134	1.01E-12	3
GDI2	9.80E-17	0.353058017	0.552	0.386	1.54E-12	3
ACP5	2.13E-16	0.390984868	0.257	0.13	3.34E-12	3
MT-ND4L	2.60E-16	0.373876916	0.466	0.31	4.07E-12	3
EIF1B	9.57E-16	0.365972253	0.39	0.244	1.50E-11	3
ANKRD10	1.62E-15	0.347355961	0.281	0.15	2.54E-11	3
HSP90AB1	3.70E-15	0.369417714	0.579	0.433	5.80E-11	3
TRA2B	1.11E-14	0.347658483	0.537	0.392	1.74E-10	3
HMGN1	2.41E-14	0.268700663	0.783	0.647	3.78E-10	3
ARL5A	2.80E-14	0.321986546	0.43	0.279	4.38E-10	3
WDR74	3.44E-14	0.353879598	0.29	0.162	5.40E-10	3
ST13	1.23E-13	0.324963405	0.543	0.392	1.93E-09	3
LAMTOR1	1.24E-13	0.343978473	0.499	0.352	1.94E-09	3
GPX1	1.85E-13	0.334091122	0.454	0.31	2.90E-09	3
LSM7	2.29E-13	0.341694615	0.515	0.374	3.59E-09	3
NPC2	2.36E-13	0.321289185	0.315	0.187	3.70E-09	3

Table S2 Continued

Gene_marker	*P*_val	Avg_logFC	Pct.1	Pct.2	*P*_val_adj	Cluster
ESD	2.84E-13	0.31652421	0.35	0.212	4.45E-09	3
AP1S2	3.19E-13	0.343385066	0.273	0.152	5.00E-09	3
MDM4	3.22E-13	0.469390729	0.375	0.244	5.04E-09	3
EIF3L	3.67E-13	0.310789888	0.678	0.539	5.75E-09	3
SNX3	3.86E-13	0.323038	0.579	0.441	6.05E-09	3
SET	6.16E-13	0.353343497	0.506	0.36	9.66E-09	3
C12orf57	1.60E-12	0.385741237	0.603	0.497	2.52E-08	3
TTC3	3.20E-12	0.284173403	0.37	0.232	5.02E-08	3
NEMF	4.27E-12	0.266728186	0.268	0.149	6.70E-08	3
AKAP9	4.31E-12	0.324278099	0.362	0.235	6.75E-08	3
IKZF3	4.63E-12	0.357082157	0.282	0.166	7.26E-08	3
SPG21	1.60E-11	0.300834825	0.301	0.183	2.51E-07	3
RBM23	3.27E-11	0.322570545	0.271	0.163	5.13E-07	3
KMT2E	6.57E-10	0.301099657	0.439	0.317	1.03E-05	3
PEBP1	1.19E-09	0.268512762	0.528	0.412	1.86E-05	3
CDK5RAP3	1.30E-09	0.279936204	0.271	0.169	2.04E-05	3
RSF1	1.35E-08	0.269047322	0.264	0.167	0.000211047	3
CHCHD10	1.95E-08	0.293867431	0.326	0.224	0.000305297	3
TNRC6B	3.51E-08	0.292871807	0.404	0.304	0.000549988	3
SFPQ	3.70E-08	0.256689815	0.359	0.252	0.000579975	3
RNASEH2B	3.72E-08	0.275210003	0.313	0.217	0.00058327	3
EIF2S3	4.38E-08	0.277537187	0.321	0.224	0.00068603	3
ATP5O	1.34E-07	0.252062683	0.521	0.433	0.002096307	3
NFKBIA	2.27E-07	0.34411828	0.279	0.19	0.003552291	3
SLC2A3	2.42E-07	0.259015818	0.332	0.234	0.003786889	3
SLC38A2	4.68E-07	0.259340475	0.346	0.252	0.007334196	3
CCND1	3.11E-69	0.767314033	0.799	0.182	4.88E-65	4
MS4A1	3.05E-64	0.673833584	0.878	0.228	4.79E-60	4
CD79B	9.98E-58	0.71033081	0.915	0.29	1.56E-53	4
EGR1	5.79E-56	0.790014526	0.713	0.182	9.07E-52	4

Table S2 Continued

Gene_marker	*P*_val	Avg_logFC	Pct.1	Pct.2	*P*_val_adj	Cluster
IGLC7	5.18E-55	0.679873532	0.774	0.207	8.12E-51	4
CD79A	2.37E-53	0.634841931	0.848	0.249	3.72E-49	4
BTG2	3.89E-53	0.795356092	0.854	0.294	6.10E-49	4
IGLC6	3.80E-52	0.481392546	0.671	0.157	5.95E-48	4
IGLC3	1.24E-50	0.295402194	0.976	0.472	1.95E-46	4
IGLC2	2.45E-48	0.347225889	1	0.945	3.83E-44	4
VPREB3	1.26E-47	0.670434248	0.665	0.174	1.97E-43	4
MARCH1	7.32E-44	0.625698494	0.39	0.073	1.15E-39	4
ARHGAP24	3.05E-39	0.554082635	0.415	0.087	4.77E-35	4
CD74	2.08E-38	0.598285058	1	0.936	3.26E-34	4
CD24	1.29E-36	0.486674571	0.482	0.115	2.02E-32	4
IGHD	5.65E-36	0.455572494	0.415	0.092	8.86E-32	4
RP11-231C14.7	2.59E-35	0.492433031	0.402	0.089	4.06E-31	4
MT-ND4	8.80E-35	0.450337734	1	0.985	1.38E-30	4
HLA-DRA	1.05E-31	0.537652465	0.963	0.604	1.65E-27	4
HLA-DMB	5.45E-31	0.543082938	0.53	0.157	8.55E-27	4
IER2	7.62E-30	0.60201819	0.945	0.562	1.19E-25	4
RP5-887A10.1	1.19E-29	0.594663797	0.366	0.088	1.87E-25	4
RALGPS2	4.58E-29	0.447599096	0.348	0.079	7.18E-25	4
PHACTR1	7.79E-29	0.516951589	0.311	0.067	1.22E-24	4
FADS3	8.59E-28	0.475538053	0.354	0.085	1.35E-23	4
TCF4	5.22E-27	0.441308153	0.329	0.077	8.18E-23	4
CD19	7.17E-27	0.409338215	0.402	0.107	1.12E-22	4
KLF6	1.73E-26	0.582141464	0.945	0.673	2.72E-22	4
LINC00926	2.60E-26	0.43151208	0.305	0.069	4.07E-22	4
H3F3B	2.64E-26	0.46571021	1	0.956	4.13E-22	4
MT-ND3	1.05E-24	0.380952708	1	0.98	1.64E-20	4
CH17-373J23.1	2.52E-24	0.521421537	0.616	0.236	3.94E-20	4
TNFRSF13C	4.51E-24	0.374093164	0.421	0.122	7.06E-20	4
SOX11	7.41E-24	0.408549825	0.299	0.071	1.16E-19	4

Table S2 Continued

Gene_marker	*P*_val	Avg_logFC	Pct.1	Pct.2	*P*_val_adj	Cluster
FAM129C	9.90E-24	0.375606952	0.354	0.093	1.55E-19	4
BLNK	3.64E-23	0.381157758	0.299	0.073	5.71E-19	4
MALAT1	3.35E-22	0.260883932	1	1	5.26E-18	4
CD9	1.05E-21	0.399230389	0.348	0.098	1.64E-17	4
CD69	2.11E-21	0.460281783	0.902	0.594	3.31E-17	4
ORAI2	5.24E-21	0.372509943	0.427	0.136	8.21E-17	4
CCR7	6.91E-21	0.541840061	0.415	0.14	1.08E-16	4
CXCR4	1.15E-19	0.565701781	0.915	0.587	1.80E-15	4
SPIB	1.46E-19	0.343062093	0.274	0.071	2.29E-15	4
ZFP36L1	6.09E-19	0.502438259	0.768	0.412	9.55E-15	4
MT-ND1	1.23E-18	0.413669242	0.976	0.79	1.93E-14	4
CLECL1	2.65E-18	0.304428651	0.36	0.112	4.15E-14	4
TCL1A	4.25E-18	0.278089444	0.305	0.087	6.67E-14	4
SMARCB1	5.38E-18	0.379117176	0.494	0.181	8.44E-14	4
STX7	1.35E-17	0.400674452	0.384	0.129	2.11E-13	4
AP3B1	2.21E-17	0.382630527	0.36	0.117	3.46E-13	4
FCMR	5.89E-17	0.417884802	0.567	0.239	9.24E-13	4
MT-CO3	7.39E-17	0.274798223	1	0.994	1.16E-12	4
SMIM14	1.49E-16	0.292396918	0.47	0.174	2.34E-12	4
MT-CO2	3.00E-16	0.272675003	1	0.997	4.71E-12	4
RP11-386I14.4	3.04E-16	0.486145084	0.652	0.323	4.76E-12	4
FCRL2	6.85E-16	0.342570188	0.317	0.102	1.07E-11	4
CD72	7.16E-16	0.296477947	0.274	0.081	1.12E-11	4
POU2F2	1.14E-15	0.331006355	0.39	0.139	1.79E-11	4
ITM2C	3.07E-15	0.297925052	0.445	0.167	4.82E-11	4
MT-ND2	5.04E-15	0.326013099	1	0.979	7.89E-11	4
GNG11	7.21E-15	0.308458952	0.299	0.095	1.13E-10	4
FOSB	1.07E-13	0.43794152	0.591	0.304	1.67E-09	4
CELF1	1.62E-13	0.369444957	0.415	0.167	2.53E-09	4
LHPP	3.39E-13	0.333525581	0.274	0.093	5.32E-09	4

Table S2 Continued

Gene_marker	*P*_val	Avg_logFC	Pct.1	Pct.2	*P*_val_adj	Cluster
HLA-DMA	4.57E-13	0.332860251	0.61	0.29	7.16E-09	4
RHOB	4.58E-13	0.318955189	0.305	0.11	7.18E-09	4
NFKBIZ	5.24E-13	0.309685533	0.262	0.087	8.22E-09	4
MT-CYB	5.44E-13	0.292790309	1	0.963	8.52E-09	4
CD37	6.42E-13	0.292563439	0.963	0.719	1.01E-08	4
JUN	8.82E-13	0.273215597	0.988	0.752	1.38E-08	4
ZBTB20	3.26E-12	0.32675279	0.305	0.111	5.11E-08	4
HERPUD1	4.86E-12	0.362910144	0.628	0.333	7.62E-08	4
MARCKSL1	5.03E-12	0.362718881	0.488	0.227	7.88E-08	4
NCF1	8.17E-12	0.370414776	0.439	0.197	1.28E-07	4
RP11-347P5.1	2.47E-11	0.431981334	0.738	0.465	3.88E-07	4
FOS	4.78E-11	0.330830043	0.762	0.465	7.49E-07	4
LINC01089	4.78E-11	0.282878116	0.274	0.101	7.50E-07	4
REL	8.23E-11	0.265018588	0.329	0.129	1.29E-06	4
PNISR	1.04E-10	0.311641217	0.915	0.632	1.63E-06	4
IRF9	2.30E-10	0.283759632	0.317	0.129	3.61E-06	4
PNN	3.87E-10	0.392844695	0.573	0.321	6.06E-06	4
RP5-1171I10.5	5.68E-10	0.348740531	0.543	0.295	8.91E-06	4
XRRA1	7.71E-10	0.263184831	0.28	0.11	1.21E-05	4
HLA-DQA1	8.44E-10	0.264541646	0.555	0.284	1.32E-05	4
TNRC6A	9.57E-10	0.253003179	0.256	0.098	1.50E-05	4
NEMF	1.10E-09	0.299550002	0.354	0.157	1.72E-05	4
TUBA1A	1.20E-09	0.372776259	0.567	0.318	1.88E-05	4
DUSP22	1.34E-09	0.29043995	0.274	0.112	2.10E-05	4
RCSD1	2.03E-09	0.312167533	0.53	0.279	3.19E-05	4
JMJD1C	2.30E-09	0.258062553	0.323	0.138	3.61E-05	4
ERV3-1	2.60E-09	0.256313813	0.256	0.1	4.07E-05	4
DUSP2	3.41E-09	0.352921308	0.78	0.558	5.34E-05	4
MIDN	3.93E-09	0.288098146	0.293	0.124	6.16E-05	4
TMEM243	5.87E-09	0.279242711	0.451	0.223	9.20E-05	4

Table S2 Continued

Gene_marker	*P*_val	Avg_logFC	Pct.1	Pct.2	*P*_val_adj	Cluster
JUND	6.93E-09	0.299833803	0.47	0.242	0.000108653	4
LINC00969	7.05E-09	0.252992543	0.287	0.12	0.000110561	4
SNHG7	7.52E-09	0.261789815	0.409	0.198	0.000117828	4
FCGRT	1.08E-08	0.264352067	0.293	0.126	0.000168818	4
TAF7	1.13E-08	0.291970081	0.524	0.278	0.000177859	4
PRDM2	1.26E-08	0.26933168	0.335	0.149	0.000197396	4
RBM23	1.48E-08	0.251560484	0.366	0.17	0.000231717	4
ZFP36	2.25E-08	0.292345442	0.665	0.422	0.000352303	4
DUSP1	4.37E-08	0.25737334	0.921	0.73	0.000685686	4
POLR2J3	5.97E-08	0.272237559	0.738	0.471	0.00093621	4
MARK3	1.36E-07	0.257063064	0.305	0.141	0.002128203	4
SEC62	2.09E-07	0.267132132	0.72	0.468	0.003270158	4
PRKCB	5.04E-07	0.252580611	0.512	0.284	0.007903225	4
KLRF1	0	2.242124133	0.863	0.032	0	5
KLRC1	2.21E-297	2.043367204	0.634	0.017	3.46E-293	5
TRDC	6.63E-294	1.931205686	0.779	0.033	1.04E-289	5
FCER1G	6.61E-240	1.855822643	0.779	0.044	1.04E-235	5
TYROBP	2.91E-219	2.151456962	0.901	0.078	4.57E-215	5
SH2D1B	1.50E-173	0.909052169	0.321	0.006	2.36E-169	5
IGFBP7	4.26E-156	0.874526849	0.298	0.006	6.67E-152	5
GNLY	2.73E-94	2.723132533	0.992	0.415	4.28E-90	5
KLRC2	1.41E-90	0.925866042	0.252	0.011	2.21E-86	5
IFITM3	2.50E-78	1.125425877	0.527	0.07	3.92E-74	5
GZMB	1.71E-69	1.784732992	0.916	0.305	2.68E-65	5
KLRD1	2.99E-61	1.263424072	0.962	0.437	4.69E-57	5
XCL1	1.29E-59	0.908443808	0.282	0.025	2.02E-55	5
HOPX	3.80E-57	1.291944956	0.748	0.216	5.96E-53	5
PLAC8	5.38E-55	1.197874936	0.695	0.184	8.43E-51	5
ID2	4.94E-51	1.213664129	0.924	0.422	7.74E-47	5
CTSW	1.94E-43	0.991041423	0.969	0.592	3.04E-39	5

Table S2 Continued

Gene_marker	*P*_val	Avg_logFC	Pct.1	Pct.2	*P*_val_adj	Cluster
FCGR3A	1.35E-42	0.964280937	0.489	0.104	2.11E-38	5
NKG7	6.80E-42	0.924740766	0.992	0.828	1.07E-37	5
CD7	2.92E-41	1.217271549	0.832	0.4	4.58E-37	5
IL2RB	5.85E-38	0.970288789	0.542	0.143	9.17E-34	5
TRGC1	1.41E-37	0.86436367	0.42	0.086	2.22E-33	5
IFITM2	5.97E-37	1.090718996	0.901	0.585	9.36E-33	5
NCR3	4.22E-36	0.662738321	0.313	0.051	6.61E-32	5
SRGN	1.08E-35	0.886395147	0.954	0.702	1.69E-31	5
CD300A	5.57E-34	0.773698749	0.305	0.052	8.74E-30	5
CMC1	1.42E-33	0.969731961	0.901	0.5	2.23E-29	5
XCL2	5.95E-27	0.891345548	0.466	0.139	9.32E-23	5
KLRB1	1.66E-26	0.923972139	0.595	0.215	2.60E-22	5
GPR65	8.16E-26	0.782895453	0.496	0.159	1.28E-21	5
SPON2	1.96E-25	1.166311398	0.481	0.164	3.07E-21	5
PRF1	6.57E-25	0.941528478	0.748	0.374	1.03E-20	5
CD63	6.61E-24	0.811796574	0.695	0.336	1.04E-19	5
PRSS23	2.42E-22	0.607057991	0.313	0.075	3.79E-18	5
FGFBP2	1.49E-19	0.936151839	0.649	0.307	2.34E-15	5
CST7	3.04E-19	0.607811752	0.931	0.685	4.77E-15	5
CHST12	1.38E-18	0.625485374	0.618	0.273	2.16E-14	5
TBX21	2.16E-18	0.663603468	0.412	0.139	3.39E-14	5
S1PR5	2.33E-18	0.623990709	0.45	0.161	3.65E-14	5
UBE2F	9.30E-18	0.608682941	0.382	0.129	1.46E-13	5
GZMA	1.79E-17	0.559437474	0.939	0.625	2.80E-13	5
RHOC	3.82E-17	0.672622357	0.45	0.175	5.99E-13	5
FGR	3.85E-17	0.665690827	0.458	0.182	6.03E-13	5
EFHD2	1.51E-16	0.59888965	0.42	0.156	2.37E-12	5
HCST	1.96E-16	0.480784124	0.962	0.806	3.07E-12	5
SELL	1.08E-15	1.035943497	0.519	0.253	1.69E-11	5
TTC38	3.41E-15	0.560340072	0.313	0.101	5.35E-11	5

Table S2 Continued

Gene_marker	*P*_val	Avg_logFC	Pct.1	Pct.2	*P*_val_adj	Cluster
CLIC3	9.18E-15	0.813261407	0.435	0.186	1.44E-10	5
LAIR2	5.88E-14	0.605315096	0.321	0.111	9.22E-10	5
RAP1B	6.72E-14	0.434185858	0.809	0.513	1.05E-09	5
MATK	1.19E-13	0.563249473	0.42	0.173	1.87E-09	5
MYO1F	1.56E-13	0.471130529	0.756	0.47	2.45E-09	5
C1orf162	2.79E-13	0.592165236	0.382	0.153	4.38E-09	5
DDIT4	5.71E-13	0.523461489	0.458	0.209	8.95E-09	5
IFITM1	1.13E-12	0.577419585	0.603	0.343	1.78E-08	5
ADGRG1	2.88E-12	0.519010731	0.328	0.123	4.52E-08	5
CD247	6.64E-12	0.632254145	0.595	0.331	1.04E-07	5
XBP1	5.82E-11	0.453005882	0.588	0.314	9.12E-07	5
RP11-160E2.6	7.12E-11	0.644634679	0.466	0.242	1.12E-06	5
LY6E	1.02E-10	0.488143262	0.809	0.616	1.60E-06	5
SAMD3	1.41E-10	0.41708839	0.496	0.238	2.21E-06	5
MT-CO1	1.59E-10	0.254641702	1	0.998	2.49E-06	5
ITGB2	5.00E-10	0.430486289	0.824	0.667	7.84E-06	5
HMGN3	6.06E-10	0.484946957	0.557	0.328	9.50E-06	5
CTSD	7.52E-10	0.517599635	0.458	0.244	1.18E-05	5
DRAP1	3.85E-09	0.355719811	0.718	0.473	6.04E-05	5
GZMH	4.66E-09	0.411273418	0.817	0.555	7.30E-05	5
AC092580.4	5.70E-09	0.523766339	0.626	0.422	8.94E-05	5
FAM49B	5.83E-09	0.470491271	0.679	0.468	9.14E-05	5
PTGER2	6.71E-09	0.441014665	0.26	0.104	0.000105263	5
TSPAN32	1.22E-08	0.417096746	0.282	0.117	0.000191762	5
BST2	1.26E-08	0.428408509	0.534	0.312	0.000198178	5
NDUFB7	1.45E-08	0.464893979	0.565	0.364	0.00022771	5
SDF4	2.45E-08	0.398526343	0.366	0.181	0.000383347	5
NEAT1	3.98E-08	0.325967879	0.817	0.639	0.000623124	5
PLEK	5.69E-08	0.382015083	0.557	0.323	0.000891529	5
LIMD2	1.27E-07	0.38256057	0.832	0.733	0.001991468	5

Table S2 Continued

Gene_marker	*P*_val	Avg_logFC	Pct.1	Pct.2	*P*_val_adj	Cluster
ATM	1.89E-07	0.380625597	0.466	0.265	0.002955339	5
DENND2D	2.43E-07	0.41669624	0.466	0.278	0.00381564	5
JAK1	4.59E-07	0.49693986	0.466	0.297	0.007196221	5
MT-ATP6	7.11E-07	0.273485264	0.939	0.902	0.011142771	5
SERPINB1	8.31E-07	0.459395067	0.382	0.215	0.013029933	5
POLR2L	9.52E-07	0.34938733	0.748	0.582	0.014928331	5
RARRES3	2.10E-06	0.299213014	0.786	0.602	0.032849825	5
PYHIN1	2.51E-06	0.38168525	0.557	0.361	0.039390676	5
LITAF	3.09E-06	0.332462831	0.725	0.552	0.048403664	5
GSTP1	3.69E-06	0.312085589	0.695	0.527	0.057884641	5
ZAP70	3.90E-06	0.333450356	0.473	0.281	0.061090846	5
C19orf66	4.01E-06	0.365663647	0.405	0.232	0.062854501	5
LINC01420	4.48E-06	0.33042679	0.336	0.182	0.070153453	5
ABI3	5.37E-06	0.421466294	0.351	0.199	0.084169892	5
TPST2	6.32E-06	0.379004458	0.405	0.243	0.099086912	5
ARPC5L	6.58E-06	0.340183247	0.489	0.31	0.10314337	5
UTS2	7.27E-06	0.308559383	0.359	0.196	0.113958507	5
DGKZ	7.83E-06	0.372212673	0.328	0.182	0.122752023	5
ABHD17A	8.74E-06	0.368652933	0.275	0.142	0.13698167	5
TRAPPC1	8.87E-06	0.293388167	0.672	0.501	0.139117989	5
C5orf56	9.18E-06	0.357682753	0.405	0.241	0.143960702	5
BIN2	1.12E-05	0.302913577	0.595	0.429	0.175530847	5
CH17-189H20.1	1.23E-05	0.378639288	0.389	0.234	0.193241717	5
MRPS6	1.28E-05	0.415751944	0.29	0.158	0.200013987	5
PTPN4	1.34E-05	0.345211469	0.328	0.182	0.209925421	5
OSTF1	1.60E-05	0.359786522	0.466	0.309	0.250794167	5
TRABD	1.68E-05	0.362001761	0.366	0.218	0.263651356	5
PIP4K2A	1.97E-05	0.364330074	0.389	0.238	0.308150254	5
STARD3NL	2.45E-05	0.311088847	0.267	0.143	0.384372825	5
CHD9	2.46E-05	0.33651378	0.29	0.156	0.386262147	5

Table S2 Continued

Gene_marker	*P*_val	Avg_logFC	Pct.1	Pct.2	*P*_val_adj	Cluster
ADAM8	3.75E-05	0.362368516	0.275	0.147	0.587478838	5
NDUFB2	3.96E-05	0.304792545	0.641	0.489	0.620516716	5
PRDX5	4.57E-05	0.334724524	0.511	0.368	0.717105996	5
NSMCE1	4.69E-05	0.320995917	0.321	0.185	0.734552756	5
TIMM13	5.13E-05	0.378711811	0.298	0.172	0.804283977	5
RNF213	5.35E-05	0.297797219	0.534	0.362	0.839000602	5
IKZF1	5.35E-05	0.264925149	0.366	0.211	0.839414072	5
DBI	5.46E-05	0.279015263	0.634	0.474	0.855362033	5
CD2	5.68E-05	0.276679881	0.756	0.603	0.891088261	5
STOM	5.75E-05	0.285265682	0.389	0.232	0.902060488	5
ARID5A	5.80E-05	0.29568152	0.26	0.136	0.909369348	5
C9orf142	5.89E-05	0.297243595	0.58	0.419	0.92322652	5
ARL4C	6.49E-05	0.365658545	0.466	0.309	1	5
LPCAT1	6.75E-05	0.25880793	0.26	0.134	1	5
SEC11A	7.92E-05	0.277924268	0.473	0.312	1	5
TCEB2	8.72E-05	0.260370067	0.786	0.697	1	5
SUN2	0.000101264	0.300784918	0.58	0.436	1	5
AKNA	0.00011485	0.350768827	0.412	0.279	1	5
NARF	0.000130591	0.270390699	0.252	0.137	1	5
SERBP1	0.000162861	0.33859728	0.534	0.389	1	5
CD164	0.000200579	0.272052389	0.634	0.489	1	5
RUNX3	0.000218807	0.265998296	0.26	0.142	1	5
RBM8A	0.00025086	0.317731237	0.473	0.349	1	5
PPP1R18	0.000254328	0.270596527	0.534	0.398	1	5
APMAP	0.000273618	0.35413775	0.42	0.277	1	5
ITGAL	0.000342897	0.250972562	0.359	0.217	1	5
CPNE1	0.000351174	0.279597836	0.313	0.189	1	5
GIMAP6	0.000352532	0.276963239	0.275	0.161	1	5
TPI1	0.000372132	0.31225363	0.595	0.475	1	5
TGFB1	0.000391833	0.270346615	0.275	0.158	1	5

Table S2 Continued

Gene_marker	*P*_val	Avg_logFC	Pct.1	Pct.2	*P*_val_adj	Cluster
LGALS1	0.00044951	0.320556203	0.557	0.44	1	5
PYCARD	0.000484197	0.260546629	0.435	0.295	1	5
DR1	0.000593854	0.271749296	0.282	0.175	1	5
CAST	0.00060675	0.348354628	0.344	0.225	1	5
PSMA1	0.000641724	0.256204033	0.458	0.325	1	5
LSM2	0.000665238	0.256632758	0.305	0.192	1	5
DYNLRB1	0.000699316	0.295730973	0.427	0.304	1	5
SPN	0.00071822	0.26191142	0.374	0.25	1	5
WDR74	0.000742919	0.317268242	0.282	0.176	1	5
FCRL6	0.000829888	0.250552878	0.267	0.157	1	5
CCDC85B	0.000941759	0.281903146	0.443	0.326	1	5
COX7A2L	0.000946284	0.262154019	0.557	0.416	1	5
IL10RA	0.0010297	0.31737324	0.427	0.314	1	5
MBP	0.001129941	0.295789417	0.405	0.286	1	5
SLA	0.001227328	0.258717094	0.328	0.209	1	5
ATF4	0.001294782	0.259946079	0.305	0.197	1	5
FLOT1	0.001344158	0.267233423	0.282	0.176	1	5
CLK1	0.00146978	0.250245608	0.382	0.264	1	5
SIGIRR	0.001477674	0.274665926	0.45	0.333	1	5
SAP18	0.001516558	0.251891551	0.55	0.428	1	5
DHRS7	0.001556288	0.273191265	0.382	0.27	1	5
EIF3J	0.001799743	0.288313114	0.374	0.271	1	5
DYNLL1	0.00182394	0.264133134	0.557	0.434	1	5
MAP1LC3B	0.001948835	0.253635669	0.412	0.29	1	5
CCND3	0.002161163	0.3006491	0.565	0.445	1	5
DOK2	0.002238324	0.335931454	0.443	0.328	1	5
SPCS3	0.002418507	0.251397731	0.282	0.182	1	5
UBE2L6	0.002807822	0.259034553	0.435	0.32	1	5
FIS1	0.003422225	0.251575135	0.382	0.277	1	5
CCDC69	0.003695378	0.259118288	0.282	0.189	1	5

Table S2 Continued

Gene_marker	*P*_val	Avg_logFC	Pct.1	Pct.2	*P*_val_adj	Cluster
SHKBP1	0.003909192	0.258775933	0.29	0.191	1	5
CTDSP1	0.003925109	0.257071137	0.252	0.163	1	5
TMEM134	0.003966342	0.273189522	0.252	0.164	1	5
ETS1	0.004744757	0.273991681	0.359	0.252	1	5
VAMP8	0.005304367	0.289326618	0.542	0.438	1	5
VPS29	0.006105706	0.266601322	0.321	0.228	1	5
EIF3G	0.006959053	0.272940792	0.542	0.452	1	5
FLNA	0.008539399	0.317338835	0.366	0.287	1	5
TNFRSF17	0	1.110566148	0.781	0.009	0	6
TXNDC5	0	0.535638049	0.562	0.005	0	6
SDC1	0	0.505858246	0.589	0.003	0	6
SPAG4	0	0.440217791	0.507	0.003	0	6
RP11-16E12.2	8.89E-282	0.39051497	0.397	0.002	1.39E-277	6
TRIB1	3.76E-257	0.562106926	0.589	0.01	5.89E-253	6
GAS6	9.70E-256	0.264306953	0.411	0.003	1.52E-251	6
JSRP1	1.33E-255	0.810301936	0.548	0.008	2.09E-251	6
EAF2	9.90E-252	0.74974738	0.685	0.016	1.55E-247	6
PYCR1	1.63E-243	0.288353193	0.37	0.002	2.56E-239	6
GNG7	2.68E-228	0.611336075	0.616	0.014	4.20E-224	6
DERL3	5.07E-178	1.644166938	0.973	0.065	7.94E-174	6
ELL2	7.06E-165	0.365533765	0.411	0.008	1.11E-160	6
CHPF	1.94E-159	0.431084142	0.534	0.017	3.04E-155	6
QPRT	2.03E-153	0.274090851	0.384	0.008	3.18E-149	6
IGKV1-12	2.26E-149	1.279742679	0.452	0.013	3.54E-145	6
KIAA0125	1.47E-139	0.71341401	0.411	0.011	2.30E-135	6
CPNE5	2.34E-123	0.34041057	0.425	0.014	3.66E-119	6
PRDX4	1.40E-119	1.250268974	0.877	0.08	2.19E-115	6
SEMA4A	1.03E-112	0.324466381	0.37	0.011	1.61E-108	6
CD38	6.19E-108	0.588450598	0.753	0.056	9.70E-104	6
IGLV3-1	9.69E-108	0.316619374	0.329	0.009	1.52E-103	6

Table S2 Continued

Gene_marker	*P*_val	Avg_logFC	Pct.1	Pct.2	*P*_val_adj	Cluster
PDK1	7.79E-106	0.423721429	0.562	0.032	1.22E-101	6
CKAP4	1.76E-100	0.276605211	0.342	0.011	2.76E-96	6
MZB1	1.17E-99	2.711108644	1	0.178	1.84E-95	6
TXNDC11	6.17E-97	0.702708868	0.781	0.068	9.67E-93	6
MEF2C	7.60E-89	0.589655995	0.726	0.064	1.19E-84	6
RASGRP3	7.61E-85	0.294264409	0.288	0.009	1.19E-80	6
ANKRD28	3.70E-82	0.381987418	0.548	0.04	5.79E-78	6
SEL1L3	2.16E-81	0.556122924	0.699	0.065	3.39E-77	6
ITM2C	9.70E-77	1.304086053	0.986	0.164	1.52E-72	6
SEC14L1	8.01E-75	0.293779748	0.521	0.039	1.26E-70	6
JCHAIN	2.47E-73	4.089093936	0.795	0.113	3.87E-69	6
TXNDC15	2.49E-73	0.346018086	0.63	0.057	3.90E-69	6
SPATS2	1.35E-71	0.317092918	0.452	0.031	2.12E-67	6
UBE2J1	6.16E-67	0.947665303	0.863	0.123	9.65E-63	6
SEL1L	2.16E-66	0.294303842	0.493	0.04	3.39E-62	6
CD59	2.55E-64	0.556510903	0.616	0.064	4.00E-60	6
SIL1	2.54E-58	0.304907047	0.479	0.043	3.99E-54	6
ARSA	6.05E-58	0.336735179	0.603	0.066	9.48E-54	6
HIST1H2BG	7.66E-58	0.324285795	0.288	0.016	1.20E-53	6
CRELD2	3.13E-57	0.590437309	0.74	0.099	4.90E-53	6
DNAJB9	3.77E-56	0.733527434	0.822	0.123	5.90E-52	6
FAM46C	5.05E-55	0.473027672	0.658	0.082	7.92E-51	6
CREB3L2	6.98E-55	0.251392162	0.493	0.048	1.09E-50	6
SRPRB	2.99E-54	0.32431765	0.589	0.067	4.68E-50	6
GMPPB	5.85E-53	0.319575312	0.562	0.063	9.17E-49	6
LMAN1	1.15E-51	0.460372286	0.753	0.108	1.81E-47	6
TNFRSF13B	1.31E-50	0.274616043	0.384	0.032	2.05E-46	6
SSR4	7.19E-50	1.877800812	1	0.645	1.13E-45	6
FCRL5	2.43E-49	0.296049159	0.466	0.047	3.81E-45	6
LY96	2.10E-48	0.336232806	0.671	0.092	3.30E-44	6

Table S2 Continued

Gene_marker	*P*_val	Avg_logFC	Pct.1	Pct.2	*P*_val_adj	Cluster
FKBP2	8.98E-47	1.058075859	0.932	0.214	1.41E-42	6
PLPP5	5.53E-46	0.442195772	0.63	0.088	8.67E-42	6
CECR1	1.52E-45	0.263924544	0.562	0.071	2.38E-41	6
ZBP1	3.93E-45	0.359807006	0.603	0.081	6.16E-41	6
FKBP11	8.58E-45	1.292225199	0.904	0.255	1.34E-40	6
SEC11C	1.91E-44	1.057319406	0.918	0.22	2.99E-40	6
XBP1	4.38E-44	1.361604091	0.959	0.311	6.87E-40	6
SPCS3	9.38E-44	0.808667372	0.863	0.173	1.47E-39	6
IGKV3-15	1.65E-40	0.439758659	0.301	0.026	2.59E-36	6
HERPUD1	7.41E-40	1.244777479	0.986	0.333	1.16E-35	6
TPD52	3.56E-39	0.288803467	0.685	0.111	5.59E-35	6
PDIA4	1.04E-38	0.768006518	0.836	0.174	1.63E-34	6
SSR3	2.89E-38	0.714707297	0.863	0.184	4.53E-34	6
NPC2	3.65E-38	0.617570744	0.904	0.191	5.72E-34	6
IGHG2	1.03E-36	2.164210259	0.699	0.149	1.62E-32	6
ERLEC1	1.48E-36	0.46846779	0.795	0.152	2.32E-32	6
HM13	1.36E-35	0.441673502	0.795	0.154	2.14E-31	6
MANF	2.76E-35	0.790530232	0.877	0.202	4.32E-31	6
KDELR1	8.02E-35	0.286659442	0.753	0.139	1.26E-30	6
PABPC4	1.34E-34	0.455020623	0.753	0.147	2.09E-30	6
IGLV6-57	3.81E-34	0.703982526	0.479	0.07	5.97E-30	6
DNAJB11	3.33E-33	0.251747579	0.603	0.102	5.22E-29	6
HDLBP	5.40E-33	0.294041836	0.479	0.071	8.46E-29	6
HSP90B1	1.80E-30	1.03043488	0.973	0.365	2.82E-26	6
SELM	2.63E-29	0.390527022	0.671	0.136	4.12E-25	6
IGKV3-20	1.17E-28	0.4474702	0.589	0.114	1.84E-24	6
IGHA2	3.41E-28	1.946985611	0.712	0.148	5.34E-24	6
VIMP	4.49E-28	0.565840479	0.89	0.227	7.04E-24	6
SDF2L1	5.84E-28	0.560508606	0.808	0.194	9.15E-24	6
MYDGF	4.05E-26	0.627595575	0.877	0.239	6.36E-22	6

Table S2 Continued

Gene_marker	*P*_val	Avg_logFC	Pct.1	Pct.2	*P*_val_adj	Cluster
CD79A	1.65E-24	0.374109271	0.932	0.261	2.59E-20	6
ARF4	3.18E-24	0.275991622	0.767	0.18	4.98E-20	6
IGHG1	2.15E-23	4.656961088	0.959	0.764	3.38E-19	6
IGHG4	2.41E-23	4.459287464	1	0.974	3.79E-19	6
IGKC	3.59E-23	4.795639548	1	0.995	5.63E-19	6
IFI6	3.73E-23	0.307820817	0.589	0.129	5.84E-19	6
NUCB2	1.64E-21	0.373230124	0.74	0.19	2.57E-17	6
SELK	5.45E-21	0.61577799	0.932	0.305	8.55E-17	6
RNASE6	2.40E-20	0.264504965	0.37	0.067	3.76E-16	6
IGLV3-21	4.94E-20	0.355488903	0.411	0.08	7.74E-16	6
KDELR2	1.05E-19	0.26920995	0.726	0.187	1.65E-15	6
IGKV1-5	2.29E-18	0.574151726	0.356	0.069	3.59E-14	6
IGHG3	4.90E-18	3.651307515	0.904	0.354	7.68E-14	6
ATF4	1.19E-17	0.251447012	0.699	0.191	1.87E-13	6
PIM2	2.32E-16	0.289600758	0.726	0.216	3.63E-12	6
OSTC	1.40E-15	0.25467129	0.822	0.254	2.19E-11	6
LMAN2	2.95E-15	0.346821754	0.89	0.296	4.62E-11	6
HSPA5	4.92E-15	0.493755595	0.808	0.268	7.71E-11	6
SPCS1	1.80E-14	0.554935185	0.932	0.494	2.82E-10	6
IGKV4-1	4.68E-14	0.331924001	0.37	0.089	7.34E-10	6
CITED2	1.26E-13	0.554862632	0.712	0.258	1.98E-09	6
IGKV1-39	2.13E-13	0.633583462	0.26	0.053	3.33E-09	6
PDIA6	1.72E-12	0.321591091	0.836	0.287	2.69E-08	6
SPCS2	2.59E-12	0.561580207	0.918	0.469	4.06E-08	6
IGHV1-2	1.66E-10	0.688256229	0.342	0.093	2.60E-06	6
IGLC3	2.33E-06	3.176393671	0.932	0.484	0.036538105	6
IGHA1	3.46E-06	4.36483451	0.973	0.601	0.054182562	6
PPIB	2.18E-05	0.549831223	0.959	0.531	0.34133864	6
IGHM	0.00203133	3.335341371	0.836	0.45	1	6
TYMS	1.74E-266	1.149808384	0.522	0.006	2.72E-262	7

Table S2 Continued

Gene_marker	*P*_val	Avg_logFC	Pct.1	Pct.2	*P*_val_adj	Cluster
KIAA0101	3.17E-224	1.453773822	0.667	0.018	4.97E-220	7
BIRC5	1.26E-204	0.907430036	0.391	0.004	1.97E-200	7
MKI67	2.57E-190	1.17438697	0.449	0.008	4.03E-186	7
CDC20	1.74E-179	0.681787552	0.275	0.002	2.73E-175	7
ZWINT	3.60E-179	1.173900193	0.565	0.016	5.64E-175	7
CDK1	2.17E-148	0.700332215	0.319	0.005	3.40E-144	7
TK1	1.22E-146	0.908010238	0.362	0.007	1.91E-142	7
AURKB	8.06E-133	0.755535192	0.29	0.004	1.26E-128	7
CCNB2	5.54E-129	0.756060182	0.261	0.003	8.69E-125	7
CLSPN	4.04E-122	0.65281055	0.304	0.006	6.34E-118	7
CENPF	2.07E-110	1.41968494	0.333	0.009	3.25E-106	7
RRM2	8.33E-97	0.803146367	0.29	0.008	1.31E-92	7
TOP2A	9.87E-96	1.497996159	0.304	0.009	1.55E-91	7
HMGB3	9.88E-93	0.846449642	0.348	0.013	1.55E-88	7
CDKN3	1.06E-88	0.63930761	0.275	0.008	1.66E-84	7
CENPU	1.08E-88	0.622164268	0.275	0.008	1.70E-84	7
ORC6	9.67E-87	0.62059914	0.333	0.012	1.52E-82	7
STMN1	3.45E-85	2.297809923	0.971	0.172	5.41E-81	7
UBE2C	4.04E-82	0.998639695	0.275	0.009	6.33E-78	7
MYBL2	1.34E-81	0.599695553	0.261	0.008	2.09E-77	7
RAD51AP1	3.41E-81	0.496837528	0.275	0.009	5.34E-77	7
CENPW	5.90E-78	0.57803599	0.304	0.012	9.25E-74	7
BRCA1	3.83E-76	0.566335855	0.304	0.012	6.01E-72	7
GGH	6.47E-74	0.487802424	0.29	0.011	1.01E-69	7
NUSAP1	3.81E-65	0.872556051	0.275	0.012	5.97E-61	7
LMNB1	5.68E-56	0.685412585	0.449	0.039	8.90E-52	7
MCM7	3.93E-54	0.949826267	0.58	0.07	6.16E-50	7
DHFR	4.48E-53	0.58796964	0.362	0.027	7.03E-49	7
NREP	2.27E-50	0.463448242	0.29	0.018	3.57E-46	7
CKS1B	2.58E-47	1.049814082	0.464	0.052	4.04E-43	7

Table S2 Continued

Gene_marker	*P*_val	Avg_logFC	Pct.1	Pct.2	*P*_val_adj	Cluster
TUBA1B	1.44E-46	1.991089895	0.986	0.367	2.26E-42	7
YEATS4	1.61E-46	0.649626759	0.435	0.044	2.52E-42	7
PCNA	3.29E-45	1.03961907	0.536	0.072	5.15E-41	7
HELLS	6.45E-44	0.841545237	0.435	0.048	1.01E-39	7
HMGB2	4.37E-38	2.061483707	0.913	0.351	6.85E-34	7
MDK	2.86E-36	1.287873859	0.565	0.103	4.48E-32	7
H2AFZ	1.49E-35	1.385411674	0.986	0.474	2.34E-31	7
CKS2	1.03E-34	0.935403319	0.478	0.074	1.62E-30	7
TUBB	3.56E-34	1.612038781	0.942	0.4	5.58E-30	7
HMGN2	2.80E-32	1.348179172	1	0.665	4.39E-28	7
NASP	2.80E-32	0.857084556	0.623	0.127	4.40E-28	7
SMC2	3.19E-32	0.581398167	0.333	0.038	5.00E-28	7
MCM3	3.98E-32	0.628655525	0.435	0.063	6.24E-28	7
CARHSP1	4.66E-31	0.842077465	0.739	0.173	7.31E-27	7
PTTG1	1.23E-29	1.197064676	0.435	0.074	1.92E-25	7
TCL1A	1.45E-29	1.191313204	0.478	0.089	2.28E-25	7
EZH2	4.99E-29	0.491518799	0.304	0.035	7.81E-25	7
ITGB3BP	1.06E-28	0.528138656	0.406	0.06	1.67E-24	7
MCM5	1.45E-27	0.723040795	0.391	0.06	2.28E-23	7
CDCA7L	1.81E-26	0.545503441	0.362	0.053	2.83E-22	7
MAD2L1	4.60E-26	0.690393219	0.377	0.058	7.22E-22	7
NUDT1	9.34E-26	0.617769287	0.493	0.093	1.46E-21	7
RANBP1	7.34E-25	0.806610228	0.87	0.295	1.15E-20	7
IGLC6	1.27E-24	1.457675486	0.609	0.17	1.99E-20	7
PTMA	1.32E-24	0.661983799	1	0.999	2.07E-20	7
CENPM	4.51E-24	0.475574168	0.275	0.034	7.07E-20	7
DTYMK	5.57E-24	0.472899767	0.362	0.056	8.73E-20	7
HMGB1	1.03E-23	0.995879835	1	0.853	1.62E-19	7
CENPH	2.71E-22	0.4204181	0.261	0.033	4.25E-18	7
FCRLA	4.63E-22	0.687406974	0.362	0.064	7.26E-18	7

Table S2 Continued

Gene_marker	*P*_val	Avg_logFC	Pct.1	Pct.2	*P*_val_adj	Cluster
RNASEH2A	1.66E-21	0.370755648	0.275	0.038	2.60E-17	7
SYK	4.38E-21	0.456424694	0.275	0.039	6.86E-17	7
CBX5	7.23E-21	0.557193772	0.42	0.083	1.13E-16	7
TUBA1C	1.12E-20	0.432267697	0.29	0.043	1.75E-16	7
TRAF4	1.38E-20	0.607528269	0.348	0.062	2.16E-16	7
RPA3	2.84E-20	0.682117268	0.594	0.158	4.45E-16	7
DUT	2.93E-20	0.925991613	0.696	0.248	4.59E-16	7
FCRL2	2.12E-19	0.710630797	0.449	0.105	3.32E-15	7
CLECL1	3.61E-19	0.845711061	0.464	0.116	5.66E-15	7
DEK	4.12E-19	0.813493668	0.855	0.372	6.45E-15	7
HAT1	5.40E-19	0.496125869	0.493	0.114	8.47E-15	7
HIST1H4C	5.64E-19	1.720015824	0.623	0.206	8.85E-15	7
ST14	1.31E-18	0.485120512	0.275	0.044	2.06E-14	7
H3F3A	3.50E-18	0.674090574	1	0.881	5.49E-14	7
MARCKS	3.58E-18	0.640822089	0.391	0.085	5.60E-14	7
SMC4	3.97E-18	0.646065524	0.435	0.101	6.23E-14	7
ANP32B	1.50E-17	0.813950802	0.797	0.36	2.36E-13	7
TUBB4B	1.63E-17	0.768852022	0.42	0.099	2.55E-13	7
H2AFY	3.54E-17	0.63406173	0.696	0.225	5.55E-13	7
PDLIM1	3.91E-17	0.728366894	0.507	0.139	6.13E-13	7
ANP32E	5.56E-17	0.677142684	0.565	0.163	8.72E-13	7
H2AFV	6.40E-17	0.811738345	0.71	0.273	1.00E-12	7
MFAP1	7.15E-17	0.288048205	0.261	0.042	1.12E-12	7
MS4A1	1.73E-16	1.162497982	0.638	0.248	2.71E-12	7
RPL39L	1.76E-16	0.443357636	0.29	0.053	2.76E-12	7
MARCKSL1	2.48E-16	0.757168944	0.652	0.231	3.89E-12	7
SMIM14	2.94E-16	0.863055509	0.551	0.18	4.61E-12	7
HLA-DRA	4.54E-16	0.746101745	0.986	0.612	7.12E-12	7
LSM5	6.94E-16	0.730726681	0.739	0.291	1.09E-11	7
MYL6B	1.19E-15	0.356762504	0.304	0.059	1.87E-11	7

Table S2 Continued

Gene_marker	*P*_val	Avg_logFC	Pct.1	Pct.2	*P*_val_adj	Cluster
CBX3	1.48E-15	0.666868285	0.884	0.467	2.32E-11	7
SAE1	1.80E-15	0.34975176	0.333	0.068	2.82E-11	7
VPREB3	2.36E-15	0.830338971	0.565	0.188	3.69E-11	7
GTF3C5	2.80E-15	0.284049832	0.29	0.054	4.39E-11	7
SMC6	3.11E-15	0.396768656	0.348	0.076	4.88E-11	7
CCND1	3.44E-15	0.969993681	0.565	0.201	5.39E-11	7
FABP5	4.82E-15	0.6818172	0.435	0.117	7.56E-11	7
HMGN1	5.28E-15	0.625142124	0.942	0.661	8.28E-11	7
ANXA5	5.76E-15	0.525765388	0.667	0.221	9.03E-11	7
CD24	7.42E-15	0.638663763	0.449	0.124	1.16E-10	7
PKM	9.05E-15	0.69468829	0.899	0.431	1.42E-10	7
NUCKS1	9.14E-15	0.741354925	0.754	0.285	1.43E-10	7
GAPDH	1.88E-14	0.743400516	0.971	0.88	2.95E-10	7
CXXC5	2.40E-14	0.455411318	0.319	0.07	3.75E-10	7
SIVA1	2.62E-14	0.640679949	0.696	0.272	4.11E-10	7
SPTBN1	4.62E-14	0.429494144	0.261	0.05	7.24E-10	7
RAN	4.84E-14	0.671980636	0.855	0.404	7.59E-10	7
BCL2L12	1.67E-13	0.290647805	0.261	0.051	2.62E-09	7
IGLC7	1.81E-13	1.055631663	0.565	0.224	2.84E-09	7
DNAJC9	1.91E-13	0.411049099	0.348	0.083	3.00E-09	7
FADS3	2.15E-13	0.583577161	0.362	0.092	3.38E-09	7
CDCA4	2.18E-13	0.37121698	0.275	0.056	3.42E-09	7
YWHAH	3.43E-13	0.4772426	0.565	0.18	5.38E-09	7
TFDP1	4.93E-13	0.503599219	0.377	0.099	7.72E-09	7
CD74	4.96E-13	0.573761984	1	0.937	7.77E-09	7
SMC3	5.31E-13	0.372753958	0.42	0.111	8.32E-09	7
TMPO	5.78E-13	0.451050867	0.391	0.104	9.05E-09	7
ACADM	1.14E-12	0.691655439	0.507	0.172	1.78E-08	7
SMARCB1	1.26E-12	0.464889278	0.565	0.188	1.98E-08	7
ARL6IP6	1.36E-12	0.474507723	0.42	0.118	2.14E-08	7

Table S2 Continued

Gene_marker	*P*_val	Avg_logFC	Pct.1	Pct.2	*P*_val_adj	Cluster
CBX1	1.55E-12	0.345660125	0.362	0.091	2.43E-08	7
ARHGAP24	1.78E-12	0.518551733	0.362	0.096	2.80E-08	7
ZNF444	1.88E-12	0.338663816	0.261	0.054	2.94E-08	7
DNMT1	2.10E-12	0.352555576	0.377	0.097	3.30E-08	7
EXOSC8	2.14E-12	0.420322446	0.348	0.087	3.35E-08	7
MZB1	2.62E-12	0.416479765	0.551	0.186	4.11E-08	7
CCDC50	2.97E-12	0.573917832	0.348	0.093	4.66E-08	7
HNRNPA2B1	3.65E-12	0.560379411	0.942	0.718	5.72E-08	7
ACTL6A	3.68E-12	0.287968313	0.261	0.055	5.77E-08	7
BANK1	4.11E-12	0.471088403	0.333	0.086	6.44E-08	7
CENPK	4.34E-12	0.322883634	0.29	0.065	6.80E-08	7
ILF2	6.51E-12	0.474013466	0.522	0.176	1.02E-07	7
KIF20B	6.63E-12	0.455623876	0.304	0.074	1.04E-07	7
GSTP1	6.69E-12	0.615119335	0.913	0.526	1.05E-07	7
CTBP1	6.75E-12	0.381322042	0.362	0.096	1.06E-07	7
MRPL37	8.45E-12	0.256987448	0.29	0.066	1.33E-07	7
CD79B	8.49E-12	0.803707812	0.667	0.31	1.33E-07	7
HSBP1	9.59E-12	0.399347775	0.493	0.152	1.50E-07	7
CD79A	1.05E-11	0.816270422	0.609	0.267	1.64E-07	7
HAUS1	1.18E-11	0.289587987	0.304	0.072	1.84E-07	7
MDH1	1.18E-11	0.4848274	0.551	0.19	1.85E-07	7
AP3B1	1.37E-11	0.489824403	0.406	0.122	2.14E-07	7
GGCT	1.48E-11	0.364550286	0.275	0.063	2.31E-07	7
MPDU1	1.48E-11	0.301304717	0.333	0.084	2.31E-07	7
LUC7L2	1.64E-11	0.400490869	0.42	0.122	2.57E-07	7
PRDX3	1.88E-11	0.444983153	0.435	0.134	2.95E-07	7
LMO4	1.92E-11	0.331749811	0.42	0.121	3.01E-07	7
SET	2.27E-11	0.734089086	0.768	0.373	3.56E-07	7
BAZ1B	2.47E-11	0.390270988	0.348	0.093	3.87E-07	7
LGALS3BP	3.19E-11	0.3602246	0.304	0.076	5.00E-07	7

Table S2 Continued

Gene_marker	*P*_val	Avg_logFC	Pct.1	Pct.2	*P*_val_adj	Cluster
EGR1	3.54E-11	0.533288008	0.536	0.198	5.56E-07	7
ALOX5	3.98E-11	0.448632934	0.29	0.073	6.24E-07	7
RNGTT	4.39E-11	0.460468939	0.304	0.077	6.89E-07	7
RBX1	5.57E-11	0.514109239	0.768	0.341	8.72E-07	7
RUVBL2	5.80E-11	0.271200127	0.261	0.059	9.09E-07	7
VDAC3	5.99E-11	0.453990093	0.536	0.187	9.39E-07	7
LY86	6.98E-11	0.435916536	0.348	0.097	1.09E-06	7
NSL1	7.51E-11	0.340363171	0.377	0.107	1.18E-06	7
HLA-DMA	7.94E-11	0.454660346	0.71	0.296	1.25E-06	7
MT-CO3	8.42E-11	0.387814293	1	0.994	1.32E-06	7
CPSF6	8.89E-11	0.329502875	0.319	0.084	1.39E-06	7
GUSB	9.27E-11	0.378489546	0.319	0.085	1.45E-06	7
CH17-373J23.1	1.32E-10	0.694891122	0.565	0.246	2.07E-06	7
SMC1A	1.32E-10	0.337613628	0.261	0.061	2.07E-06	7
LSM3	1.95E-10	0.487299025	0.667	0.272	3.06E-06	7
MZT1	2.06E-10	0.37355015	0.406	0.123	3.23E-06	7
NUDT5	2.11E-10	0.29235217	0.42	0.125	3.31E-06	7
QRSL1	2.35E-10	0.355327428	0.333	0.092	3.69E-06	7
CD19	3.39E-10	0.373585508	0.377	0.115	5.32E-06	7
IGLC3	3.53E-10	0.668078306	0.783	0.487	5.53E-06	7
SRSF10	3.90E-10	0.533017948	0.58	0.24	6.11E-06	7
UBE2E3	4.03E-10	0.426377386	0.435	0.142	6.32E-06	7
NAP1L1	4.13E-10	0.494014083	0.884	0.575	6.47E-06	7
ITGAE	5.02E-10	0.367799504	0.377	0.115	7.87E-06	7
EAPP	5.10E-10	0.404896093	0.522	0.189	7.99E-06	7
LSM2	5.23E-10	0.359722102	0.536	0.19	8.20E-06	7
EWSR1	5.62E-10	0.426566178	0.638	0.261	8.81E-06	7
CAPRIN1	5.63E-10	0.285523762	0.348	0.098	8.82E-06	7
CCT2	8.28E-10	0.331989248	0.435	0.139	1.30E-05	7
PHACTR1	9.00E-10	0.427742264	0.275	0.073	1.41E-05	7

Table S2 Continued

Gene_marker	*P*_val	Avg_logFC	Pct.1	Pct.2	*P*_val_adj	Cluster
IGHM	1.05E-09	0.279378157	0.797	0.451	1.65E-05	7
TACC1	1.09E-09	0.291259684	0.362	0.106	1.70E-05	7
ITM2C	1.12E-09	0.437668318	0.478	0.173	1.76E-05	7
KHDRBS1	1.21E-09	0.465594335	0.667	0.287	1.89E-05	7
HNRNPA1	1.26E-09	0.371964168	0.971	0.91	1.97E-05	7
EBNA1BP2	1.31E-09	0.293377941	0.275	0.071	2.06E-05	7
CHI3L2	1.32E-09	0.35591517	0.406	0.129	2.07E-05	7
CLNS1A	1.45E-09	0.344626962	0.58	0.214	2.27E-05	7
PDZD11	1.49E-09	0.315101025	0.275	0.072	2.33E-05	7
MAD2L2	1.57E-09	0.302414876	0.333	0.095	2.45E-05	7
TMEM107	1.89E-09	0.459133115	0.536	0.207	2.96E-05	7
SNRPD1	2.11E-09	0.471687728	0.652	0.287	3.30E-05	7
UBE2E1	2.11E-09	0.325871352	0.362	0.111	3.31E-05	7
SNRPF	2.13E-09	0.429581453	0.739	0.319	3.33E-05	7
SLC25A5	2.20E-09	0.544830983	0.696	0.337	3.44E-05	7
SUMO2	2.23E-09	0.430121774	0.913	0.7	3.49E-05	7
SLBP	2.29E-09	0.463816851	0.435	0.154	3.58E-05	7
HNRNPR	2.34E-09	0.455527536	0.681	0.281	3.67E-05	7
PMAIP1	2.35E-09	0.394852672	0.391	0.125	3.68E-05	7
IGHD	2.72E-09	0.376283578	0.333	0.102	4.27E-05	7
PPIL3	2.77E-09	0.39686032	0.348	0.107	4.34E-05	7
LTA4H	2.78E-09	0.317617703	0.377	0.119	4.35E-05	7
PAFAH1B3	2.81E-09	0.308747588	0.304	0.085	4.41E-05	7
SFPQ	2.84E-09	0.533010868	0.609	0.261	4.45E-05	7
PSMA4	3.03E-09	0.60092662	0.783	0.435	4.74E-05	7
RNASE6	4.60E-09	0.333377862	0.261	0.069	7.21E-05	7
HIF1A	4.63E-09	0.338039505	0.333	0.101	7.26E-05	7
CD9	4.69E-09	0.417237825	0.333	0.105	7.36E-05	7
ATP5C1	4.87E-09	0.413297401	0.667	0.298	7.63E-05	7
IDH2	6.19E-09	0.529367394	0.667	0.33	9.70E-05	7

Table S2 Continued

Gene_marker	*P*_val	Avg_logFC	Pct.1	Pct.2	*P*_val_adj	Cluster
SNRPE	6.37E-09	0.443621435	0.681	0.302	9.99E-05	7
PLGRKT	6.64E-09	0.272380536	0.348	0.106	0.000104057	7
GRN	6.81E-09	0.535520559	0.362	0.129	0.000106737	7
TIMMDC1	7.17E-09	0.290563137	0.319	0.094	0.000112324	7
NONO	7.30E-09	0.400955446	0.536	0.209	0.00011442	7
PSMB6	7.46E-09	0.384684935	0.681	0.288	0.000116866	7
IMPDH2	8.02E-09	0.261792309	0.29	0.08	0.000125696	7
HMGA1	8.08E-09	0.390578958	0.42	0.148	0.000126702	7
CLTA	8.80E-09	0.279720881	0.464	0.156	0.000137894	7
MRPL13	9.03E-09	0.312792465	0.304	0.09	0.000141614	7
HADHB	9.68E-09	0.2671593	0.362	0.113	0.000151693	7
RALGPS2	9.85E-09	0.544963027	0.29	0.087	0.000154389	7
COX5A	1.03E-08	0.408230836	0.696	0.33	0.00016109	7
LYN	1.05E-08	0.399079967	0.275	0.079	0.000165363	7
NME1	1.06E-08	0.273565429	0.333	0.102	0.000165721	7
FAM96A	1.08E-08	0.309192969	0.42	0.142	0.000169422	7
HSP90AA1	1.09E-08	0.555445462	0.855	0.61	0.000171374	7
HPRT1	1.11E-08	0.304803999	0.362	0.115	0.000173562	7
RBM42	1.12E-08	0.290442406	0.348	0.108	0.000175899	7
TNFRSF13C	1.16E-08	0.46380158	0.377	0.13	0.000181361	7
SKA2	1.27E-08	0.353296402	0.348	0.109	0.000198683	7
HDAC2	1.58E-08	0.457888425	0.391	0.14	0.000247887	7
IMMP1L	1.61E-08	0.266258435	0.29	0.083	0.000252005	7
NDUFAB1	1.71E-08	0.39972064	0.478	0.184	0.000267332	7
DNAJA1	1.71E-08	0.35001283	0.565	0.222	0.000268188	7
MTCH2	1.75E-08	0.255402634	0.29	0.082	0.00027383	7
HLA-DQA1	1.78E-08	0.366395083	0.681	0.289	0.000278879	7
METTL9	1.97E-08	0.423394525	0.58	0.235	0.000308709	7
VDAC1	2.09E-08	0.335900467	0.522	0.199	0.000328303	7
MPLKIP	2.24E-08	0.279526899	0.348	0.11	0.000350862	7

Table S2 Continued

Gene_marker	*P*_val	Avg_logFC	Pct.1	Pct.2	*P*_val_adj	Cluster
PSMD14	2.35E-08	0.303010441	0.333	0.104	0.00036839	7
ATP6V1D	2.46E-08	0.272462645	0.319	0.097	0.000385769	7
YWHAE	2.83E-08	0.472748828	0.507	0.213	0.000443964	7
TPM4	2.94E-08	0.541634727	0.435	0.166	0.000460457	7
RBBP7	3.15E-08	0.351463762	0.406	0.143	0.000494362	7
SRSF3	3.42E-08	0.483144499	0.884	0.535	0.000536175	7
RBBP4	3.42E-08	0.394696297	0.623	0.265	0.000536787	7
APLP2	3.52E-08	0.423727208	0.29	0.09	0.0005524	7
FDPS	3.64E-08	0.27470876	0.478	0.173	0.00057004	7
PTCH2	3.79E-08	0.45338405	0.29	0.09	0.000594279	7
GNAI3	3.91E-08	0.272260005	0.406	0.14	0.000612174	7
DYNLL1	3.96E-08	0.487394274	0.739	0.433	0.000621096	7
CACYBP	4.08E-08	0.393907683	0.464	0.178	0.000639536	7
HLA-DMB	4.19E-08	0.340925612	0.449	0.168	0.000657321	7
PARP15	4.64E-08	0.306849055	0.261	0.074	0.000727603	7
LGALS9	4.89E-08	0.326042422	0.362	0.124	0.00076588	7
ILF3	4.91E-08	0.35336583	0.478	0.183	0.000769104	7
DDX39A	5.10E-08	0.357648748	0.42	0.153	0.000800126	7
HSPB11	5.49E-08	0.334819871	0.58	0.241	0.000860048	7
MIS18BP1	5.54E-08	0.366147721	0.319	0.104	0.000868192	7
CD72	6.62E-08	0.401131928	0.275	0.085	0.001037142	7
RAD21	7.62E-08	0.565586975	0.449	0.187	0.001194232	7
FOS	7.68E-08	0.736622697	0.768	0.472	0.001204124	7
BTF3L4	8.60E-08	0.253250444	0.377	0.129	0.001347434	7
DRAM2	8.70E-08	0.334878653	0.478	0.185	0.001364165	7
RFC1	9.54E-08	0.272645386	0.333	0.108	0.001495935	7
TMEM126A	1.03E-07	0.295362855	0.333	0.11	0.001613874	7
BCL7B	1.06E-07	0.251734381	0.377	0.127	0.001667135	7
MMADHC	1.07E-07	0.299228385	0.522	0.203	0.001679937	7
SPIB	1.12E-07	0.270746748	0.261	0.077	0.001750626	7

Table S2 Continued

Gene_marker	*P*_val	Avg_logFC	Pct.1	Pct.2	*P*_val_adj	Cluster
TXNDC12	1.15E-07	0.330413238	0.435	0.163	0.001799652	7
MTF2	1.24E-07	0.334849751	0.377	0.134	0.001939293	7
XRCC5	1.34E-07	0.397055268	0.594	0.262	0.002100816	7
UQCRQ	1.39E-07	0.400264855	0.826	0.444	0.002179395	7
NDUFB5	1.39E-07	0.297527195	0.565	0.232	0.002183348	7
VIM	1.46E-07	0.652968527	0.812	0.564	0.002283269	7
C14orf166	1.51E-07	0.484905964	0.681	0.352	0.002365068	7
CD69	1.54E-07	0.497351735	0.884	0.602	0.002417141	7
BABAM1	1.72E-07	0.30865408	0.406	0.149	0.002701	7
CETN3	1.79E-07	0.292990039	0.29	0.091	0.002809052	7
VPS29	1.82E-07	0.354419447	0.536	0.226	0.002850729	7
ZCCHC10	1.82E-07	0.361174969	0.348	0.12	0.002854221	7
ACTG1	1.85E-07	0.496028394	0.971	0.913	0.002903723	7
PHF14	1.92E-07	0.352054316	0.478	0.189	0.003004871	7
SRP9	1.93E-07	0.426555922	0.739	0.473	0.003030682	7
PPIA	1.99E-07	0.466190625	0.913	0.782	0.003122553	7
PRDX6	2.14E-07	0.318528543	0.551	0.231	0.003349933	7
RNASEH2C	2.14E-07	0.296357793	0.522	0.211	0.003360888	7
PRDX2	2.39E-07	0.382748274	0.71	0.354	0.003752864	7
NMI	2.45E-07	0.279389118	0.304	0.099	0.00384441	7
GNG11	2.70E-07	0.469516895	0.29	0.1	0.004224798	7
METAP2	2.81E-07	0.39582139	0.449	0.182	0.004412357	7
PDCD5	2.83E-07	0.273327643	0.464	0.179	0.004429983	7
MT-CO2	3.00E-07	0.290975996	1	0.997	0.004697858	7
AP1S1	3.05E-07	0.253151463	0.29	0.092	0.004775011	7
PSMC3	3.13E-07	0.278139438	0.464	0.182	0.004899689	7
CCT4	3.34E-07	0.311682867	0.536	0.222	0.005242862	7
MED30	3.43E-07	0.267589482	0.348	0.12	0.005371519	7
PNN	3.75E-07	0.487776689	0.681	0.326	0.005871312	7
ANAPC11	3.92E-07	0.337329316	0.638	0.288	0.006149584	7

Table S2 Continued

Gene_marker	*P*_val	Avg_logFC	Pct.1	Pct.2	*P*_val_adj	Cluster
USP22	4.07E-07	0.353446168	0.261	0.082	0.006379255	7
PMF1	4.69E-07	0.261099099	0.348	0.123	0.007348743	7
SPINT2	5.05E-07	0.31162442	0.275	0.09	0.007915062	7
TMEM109	5.35E-07	0.272787545	0.42	0.159	0.008386941	7
FAM111A	5.40E-07	0.270375255	0.275	0.087	0.00846981	7
TUBA1A	5.82E-07	0.536773737	0.638	0.323	0.009126802	7
HNRNPF	6.32E-07	0.466355905	0.783	0.469	0.009900876	7
PPIH	6.59E-07	0.251710991	0.261	0.081	0.010327436	7
NFKBIZ	7.02E-07	0.29285361	0.275	0.091	0.01099859	7
VMP1	7.13E-07	0.295150488	0.493	0.199	0.011170288	7
MT-ND4	7.91E-07	0.335452182	0.986	0.986	0.012395405	7
PARP1	8.26E-07	0.341073658	0.536	0.236	0.012955249	7
TCP1	8.85E-07	0.274122308	0.406	0.156	0.013866483	7
ZSCAN16-AS1	9.38E-07	0.268319717	0.362	0.135	0.014707248	7
MAGOH	9.46E-07	0.273041801	0.536	0.223	0.014825453	7
CALM3	9.74E-07	0.54443666	0.681	0.438	0.015269706	7
TCF4	9.82E-07	0.265035331	0.261	0.085	0.015396697	7
MRPL52	1.00E-06	0.360245463	0.536	0.242	0.015707671	7
HNRNPA3	1.05E-06	0.442318548	0.739	0.415	0.016388471	7
SRSF2	1.05E-06	0.461751471	0.783	0.472	0.016469072	7
STRA13	1.15E-06	0.39131581	0.377	0.145	0.01804004	7
DNAJB11	1.19E-06	0.287979051	0.304	0.107	0.018638707	7
PMVK	1.19E-06	0.298394362	0.275	0.092	0.018699046	7
IGLC2	1.24E-06	0.632427227	1	0.946	0.0195094	7
SF3B2	1.27E-06	0.306151046	0.623	0.297	0.019981253	7
RP5-1171I10.5	1.28E-06	0.434824248	0.623	0.3	0.020006228	7
CHMP5	1.34E-06	0.311473092	0.449	0.181	0.020938622	7
RPLP0	1.34E-06	0.275621897	1	0.967	0.021003869	7
ADI1	1.37E-06	0.296772606	0.319	0.115	0.021486032	7
NAE1	1.40E-06	0.278350377	0.319	0.113	0.021941591	7

Table S2 Continued

Gene_marker	*P*_val	Avg_logFC	Pct.1	Pct.2	*P*_val_adj	Cluster
PPP1CB	1.42E-06	0.313304924	0.58	0.265	0.022287684	7
COX8A	1.52E-06	0.460077464	0.754	0.488	0.023858714	7
CNTRL	1.52E-06	0.402788047	0.319	0.115	0.023901783	7
PSAP	1.63E-06	0.475256603	0.725	0.403	0.025492903	7
UBE2I	1.64E-06	0.379219288	0.696	0.348	0.025736993	7
PSMA1	1.65E-06	0.32374472	0.667	0.323	0.025942407	7
UPF3B	1.72E-06	0.25801893	0.275	0.092	0.027021618	7
SF3A3	1.73E-06	0.298007233	0.275	0.094	0.027133839	7
CALM2	1.80E-06	0.443965276	0.87	0.714	0.028191671	7
CCDC167	1.85E-06	0.369398677	0.377	0.153	0.028973861	7
RBM8A	1.94E-06	0.312454461	0.696	0.347	0.03046036	7
DECR1	2.03E-06	0.322055158	0.406	0.166	0.03180033	7
SDCBP	2.13E-06	0.260120027	0.406	0.16	0.033368858	7
BTG2	2.14E-06	0.592411483	0.565	0.312	0.033526951	7
USP1	2.14E-06	0.287098975	0.275	0.095	0.033594661	7
BLOC1S1	2.20E-06	0.352503597	0.739	0.401	0.034434496	7
PSMB7	2.20E-06	0.264052928	0.565	0.244	0.034474519	7
HMG20B	2.24E-06	0.321191869	0.29	0.102	0.035191848	7
MZT2B	2.46E-06	0.355841313	0.754	0.39	0.038619036	7
UBL7	2.47E-06	0.250570795	0.304	0.108	0.038791541	7
NSA2	2.56E-06	0.359874048	0.536	0.246	0.040078182	7
LSM4	2.62E-06	0.316997402	0.449	0.192	0.041024777	7
HNRNPH1	2.73E-06	0.468582629	0.71	0.376	0.042808188	7
DNPH1	2.74E-06	0.250060033	0.333	0.124	0.043022974	7
ATOX1	3.14E-06	0.271887185	0.348	0.135	0.049261558	7
NUDC	3.27E-06	0.315200787	0.478	0.212	0.051203231	7
SNRPB	3.27E-06	0.375084998	0.696	0.371	0.051267379	7
EIF1AX	3.37E-06	0.298494894	0.449	0.191	0.052816136	7
ATP5G3	3.39E-06	0.364219874	0.739	0.433	0.053187405	7
ANP32A	3.53E-06	0.335668961	0.536	0.249	0.05526164	7

Table S2 Continued

Gene_marker	*P*_val	Avg_logFC	Pct.1	Pct.2	*P*_val_adj	Cluster
UCP2	3.64E-06	0.346869587	0.87	0.528	0.056988514	7
NAA38	4.06E-06	0.339964212	0.667	0.319	0.063601287	7
HNRNPA0	4.14E-06	0.415754911	0.841	0.528	0.064826823	7
DDAH2	4.19E-06	0.273538022	0.275	0.096	0.065736989	7
MRPL51	4.58E-06	0.35439017	0.536	0.263	0.071859963	7
GNAI2	5.08E-06	0.345315017	0.391	0.168	0.079655798	7
HNRNPUL1	5.39E-06	0.283383194	0.464	0.197	0.084425243	7
YBX1	5.40E-06	0.33355616	0.957	0.805	0.084653462	7
PPP4C	5.43E-06	0.372910129	0.58	0.307	0.085047972	7
SHFM1	5.98E-06	0.344082267	0.696	0.361	0.093819132	7
ANKRD10	6.13E-06	0.297251749	0.391	0.164	0.096111924	7
SSRP1	6.32E-06	0.309432864	0.333	0.13	0.099003128	7
RBM23	6.71E-06	0.310502691	0.42	0.174	0.105111096	7
TMEM243	6.95E-06	0.252450528	0.507	0.228	0.108952701	7
UQCRC1	7.16E-06	0.317012724	0.377	0.156	0.112296953	7
KTN1	7.22E-06	0.305410627	0.638	0.319	0.113166807	7
HNRNPC	7.43E-06	0.368046022	0.812	0.536	0.116432799	7
DNAJC15	7.82E-06	0.288786011	0.464	0.204	0.122655976	7
APH1A	7.85E-06	0.251360941	0.478	0.208	0.12303607	7
SNRPG	7.97E-06	0.399103905	0.725	0.422	0.124874483	7
MPC2	8.12E-06	0.308187348	0.377	0.159	0.127361623	7
EIF4H	8.32E-06	0.28272294	0.464	0.201	0.130495721	7
NDUFB6	9.01E-06	0.264337934	0.406	0.169	0.1412795	7
NUDT21	1.03E-05	0.255634132	0.319	0.123	0.161565378	7
HDAC1	1.08E-05	0.335086774	0.464	0.211	0.16903506	7
RHOB	1.14E-05	0.395518104	0.29	0.115	0.178148512	7
EIF5B	1.14E-05	0.279695629	0.638	0.311	0.17855746	7
HNRNPD	1.20E-05	0.369809379	0.42	0.193	0.188609794	7
AP2S1	1.33E-05	0.261435581	0.594	0.277	0.208810476	7
PSMB2	1.58E-05	0.308006688	0.536	0.266	0.24837671	7

Table S2 Continued

Gene_marker	*P*_val	Avg_logFC	Pct.1	Pct.2	*P*_val_adj	Cluster
TXNDC17	1.60E-05	0.265972195	0.333	0.135	0.250725533	7
APEX1	1.62E-05	0.259133849	0.406	0.177	0.253612359	7
GSTO1	1.64E-05	0.274338555	0.42	0.185	0.256446	7
DNAJC8	1.81E-05	0.349492559	0.565	0.29	0.28333065	7
ODC1	1.81E-05	0.250439715	0.319	0.126	0.283720494	7
UBE2N	1.88E-05	0.346247688	0.594	0.299	0.294022878	7
MT-ND1	1.92E-05	0.299832206	0.957	0.794	0.30170201	7
XRCC6	1.93E-05	0.351305753	0.536	0.261	0.30283261	7
HNRNPM	1.94E-05	0.40395902	0.696	0.398	0.303656028	7
BANF1	1.96E-05	0.293425871	0.522	0.254	0.307149737	7
NDUFA2	2.18E-05	0.307306958	0.58	0.313	0.341380372	7
RBM17	2.29E-05	0.360560786	0.406	0.187	0.359445027	7
CHD9	2.88E-05	0.28064463	0.362	0.156	0.451535766	7
TRA2B	3.01E-05	0.328240008	0.725	0.407	0.471124418	7
SMS	3.35E-05	0.262788246	0.42	0.19	0.524820006	7
MORF4L1	3.60E-05	0.293934019	0.841	0.534	0.563850342	7
PSMA2	3.78E-05	0.441032862	0.638	0.37	0.592977218	7
UQCRH	3.82E-05	0.32431114	0.841	0.524	0.598609144	7
HADHA	3.98E-05	0.255173982	0.478	0.225	0.623992897	7
ENY2	4.01E-05	0.318802672	0.493	0.245	0.62890234	7
PABPN1	4.45E-05	0.346340009	0.333	0.147	0.698005904	7
SLC25A3	4.58E-05	0.365636669	0.652	0.372	0.717637213	7
HN1	4.67E-05	0.348705777	0.493	0.252	0.732753384	7
ZFP36L1	4.97E-05	0.546820586	0.652	0.422	0.778397657	7
POLR2K	5.00E-05	0.29346934	0.551	0.275	0.784032017	7
CFL1	5.07E-05	0.333318438	0.986	0.961	0.794099261	7
ATP5F1	5.21E-05	0.290689385	0.623	0.335	0.817387052	7
RP11-386I14.4	5.47E-05	0.50652331	0.551	0.333	0.857209359	7
LBR	5.68E-05	0.363652199	0.449	0.223	0.890927681	7
DBI	5.86E-05	0.35624066	0.768	0.474	0.918861474	7

Table S2 Continued

Gene_marker	*P*_val	Avg_logFC	Pct.1	Pct.2	*P*_val_adj	Cluster
EIF5	5.89E-05	0.251914211	0.449	0.21	0.922594751	7
DCK	5.95E-05	0.275504017	0.565	0.29	0.93307198	7
POMP	6.28E-05	0.360741675	0.754	0.444	0.985000532	7
CYB561A3	6.38E-05	0.303797738	0.304	0.13	0.999848679	7
RCSD1	6.89E-05	0.282631952	0.551	0.284	1	7
CAPZA2	7.83E-05	0.264952889	0.536	0.262	1	7
COX17	7.83E-05	0.280820232	0.594	0.316	1	7
NDUFA12	8.26E-05	0.274408063	0.507	0.255	1	7
RHOA	8.40E-05	0.314551776	0.812	0.539	1	7
CDK5RAP3	8.72E-05	0.268433285	0.391	0.179	1	7
ROMO1	8.97E-05	0.309039196	0.623	0.342	1	7
NFATC2IP	9.04E-05	0.324652824	0.275	0.113	1	7
CALR	9.17E-05	0.439133182	0.71	0.449	1	7
PSMA3	9.53E-05	0.257282232	0.377	0.176	1	7
PSIP1	9.79E-05	0.275491934	0.565	0.292	1	7
DCTN3	0.000102792	0.297806975	0.464	0.225	1	7
TMX1	0.000104584	0.294095276	0.464	0.224	1	7
IER2	0.000104985	0.590995203	0.754	0.574	1	7
ATP5B	0.000106321	0.266071889	0.638	0.355	1	7
IFI16	0.000112358	0.267277954	0.638	0.359	1	7
WDR74	0.000119948	0.266246512	0.377	0.176	1	7
GDI2	0.000133182	0.303008435	0.696	0.404	1	7
COX6C	0.000138342	0.32692813	0.899	0.715	1	7
BUB3	0.000151011	0.317840467	0.58	0.323	1	7
EIF4E	0.000151641	0.276733422	0.348	0.16	1	7
DDX46	0.000163867	0.255670361	0.507	0.253	1	7
PARK7	0.00016686	0.317581166	0.739	0.469	1	7
OCIAD2	0.000186929	0.251564636	0.609	0.326	1	7
NDUFV2	0.000188135	0.291862711	0.478	0.251	1	7
C12orf57	0.000201986	0.260497404	0.812	0.506	1	7

Table S2 Continued

Gene_marker	*P*_val	Avg_logFC	Pct.1	Pct.2	*P*_val_adj	Cluster
EIF3L	0.000216185	0.307332885	0.841	0.553	1	7
NDUFA4	0.000220464	0.257921767	0.928	0.684	1	7
SLC38A2	0.000269172	0.274922086	0.507	0.261	1	7
NPM1	0.000272205	0.25008318	0.928	0.783	1	7
DUSP2	0.000340834	0.48481331	0.797	0.563	1	7
NCL	0.000351312	0.312461283	0.681	0.397	1	7
YWHAQ	0.00036325	0.273207632	0.768	0.477	1	7
COX7B	0.000367226	0.251913765	0.754	0.442	1	7
HLA-DRB1	0.0003953	0.257636891	0.928	0.654	1	7
APOBEC3C	0.000428003	0.25910315	0.42	0.218	1	7
ACTB	0.000455211	0.383984309	1	1	1	7
PRDX1	0.000590008	0.299965852	0.565	0.328	1	7
HMGN3	0.000620109	0.285532075	0.58	0.331	1	7
RP11-160E2.6	0.000633455	0.394381313	0.449	0.246	1	7
CHCHD2	0.000655064	0.323478157	0.855	0.696	1	7
RTN4	0.000666408	0.288252342	0.507	0.29	1	7
ARPC5	0.001046699	0.358182663	0.783	0.517	1	7
ANXA2	0.001064642	0.367069367	0.478	0.274	1	7
HNRNPK	0.001175312	0.253876715	0.87	0.646	1	7
FOXP1	0.001178888	0.252805983	0.391	0.207	1	7
ATP5J	0.001190537	0.257199131	0.725	0.454	1	7
PLP2	0.001316349	0.259940952	0.42	0.232	1	7
TPI1	0.001619564	0.306392307	0.71	0.475	1	7
NDUFB3	0.001680835	0.306731248	0.435	0.257	1	7
SEPT7	0.001708641	0.373152194	0.812	0.597	1	7
LDHA	0.002226134	0.269127768	0.638	0.414	1	7
UBXN4	0.002309522	0.261291264	0.507	0.28	1	7
PSMB8	0.002989552	0.297154942	0.652	0.482	1	7
JMJD1C	0.003777176	0.251472365	0.275	0.143	1	7
POU2F2	0.003828913	0.322637478	0.275	0.147	1	7

Table S2 Continued

Gene_marker	*P*_val	Avg_logFC	Pct.1	Pct.2	*P*_val_adj	Cluster
COTL1	0.003996602	0.451528528	0.826	0.681	1	7
H1FX	0.004528928	0.3006313	0.304	0.168	1	7
SERBP1	0.004782458	0.270192442	0.594	0.39	1	7
RPS26	0.00482023	0.253523402	0.884	0.744	1	7
ARL6IP1	0.007074741	0.772260095	0.638	0.445	1	7
MDM4	0.008503603	0.271422859	0.42	0.259	1	7
PSMA7	0.00915575	0.320918203	0.812	0.64	1	7
FCN1	0	2.860100925	0.98	0.01	0	8
SERPINA1	0	2.671569858	1	0.007	0	8
MS4A7	0	2.522592873	0.98	0.015	0	8
CFD	0	2.339139765	0.94	0.01	0	8
LINC01272	0	2.204156896	0.92	0.003	0	8
CD68	0	2.164736139	0.96	0.014	0	8
S100A9	0	2.160319107	0.9	0.015	0	8
TMEM176B	0	1.665358491	0.88	0.002	0	8
LILRB2	0	1.537165798	0.84	0.008	0	8
VMO1	0	1.501564266	0.52	0.003	0	8
LRRC25	0	1.49501645	0.76	0.002	0	8
HCK	0	1.459964011	0.68	0.006	0	8
PILRA	0	1.3853189	0.82	0.003	0	8
CSF1R	0	1.366855929	0.74	0.003	0	8
CLEC12A	0	1.344483471	0.68	0.004	0	8
CLEC7A	0	1.313019878	0.72	0.001	0	8
LILRA5	0	1.291385234	0.68	0.001	0	8
LILRA1	0	1.204739295	0.6	0.001	0	8
CPVL	0	1.17609639	0.68	0.002	0	8
CD300E	0	1.126396129	0.54	0.001	0	8
LILRB3	0	1.096368286	0.6	0.002	0	8
CDKN1C	0	1.094244225	0.54	0.003	0	8
C5AR1	0	1.067412458	0.52	0.001	0	8

Table S2 Continued

Gene_marker	*P*_val	Avg_logFC	Pct.1	Pct.2	*P*_val_adj	Cluster
LILRA2	0	0.99304584	0.56	0.002	0	8
MS4A4A	0	0.98525475	0.5	0.001	0	8
APOBEC3A	0	0.944988123	0.46	0.001	0	8
CSTA	0	0.939556137	0.44	0.001	0	8
TMEM176A	0	0.918517506	0.52	0.002	0	8
GPBAR1	0	0.857675288	0.56	0.001	0	8
FPR1	0	0.834721331	0.4	0	0	8
SLC31A2	2.41E-307	1.126455526	0.64	0.006	3.78E-303	8
KLF4	1.92E-304	1.044444172	0.46	0.002	3.01E-300	8
HK3	3.27E-297	0.792898866	0.38	0.001	5.13E-293	8
CXCL16	3.79E-291	1.079526426	0.52	0.004	5.94E-287	8
FGL2	1.72E-286	1.262466325	0.68	0.009	2.70E-282	8
CST3	5.13E-269	3.094793865	1	0.03	8.05E-265	8
TCF7L2	4.78E-268	1.220962789	0.72	0.012	7.49E-264	8
IGSF6	6.64E-268	1.022729276	0.5	0.004	1.04E-263	8
RP11-362F19.1	7.01E-263	0.708612463	0.34	0.001	1.10E-258	8
SLC11A1	4.30E-260	1.089181269	0.66	0.01	6.75E-256	8
NCF2	8.33E-257	1.175254129	0.62	0.008	1.31E-252	8
CLEC4A	5.61E-255	0.669025104	0.4	0.002	8.79E-251	8
PLAUR	1.63E-249	0.970776948	0.54	0.006	2.56E-245	8
HES4	2.31E-237	0.705046219	0.34	0.001	3.63E-233	8
HMOX1	3.04E-234	0.964224339	0.56	0.007	4.76E-230	8
FAM26F	1.76E-233	2.013257254	0.98	0.034	2.75E-229	8
CTSL	1.13E-221	0.903055305	0.4	0.003	1.77E-217	8
SPI1	1.66E-219	2.080816672	0.94	0.033	2.60E-215	8
MAFB	1.32E-214	0.927826608	0.4	0.003	2.07E-210	8
LYZ	1.91E-212	1.243118387	0.62	0.012	2.99E-208	8
RGS18	8.64E-212	0.607243309	0.28	0.001	1.35E-207	8
CFP	1.61E-205	1.262863037	0.66	0.014	2.53E-201	8
SECTM1	3.88E-199	0.621855764	0.34	0.002	6.08E-195	8

Table S2 Continued

Gene_marker	*P*_val	Avg_logFC	Pct.1	Pct.2	*P*_val_adj	Cluster
CD302	1.33E-198	0.689522609	0.36	0.003	2.08E-194	8
ADGRE2	1.36E-198	0.693957372	0.34	0.002	2.13E-194	8
MNDA	5.94E-188	1.617733818	0.8	0.027	9.31E-184	8
C19orf38	1.25E-187	1.113888738	0.66	0.017	1.96E-183	8
PRAM1	5.58E-185	0.920804671	0.4	0.004	8.74E-181	8
RETN	9.78E-185	0.915668746	0.4	0.004	1.53E-180	8
IFI30	1.55E-178	0.978802169	0.54	0.011	2.43E-174	8
FCER1G	5.74E-169	3.269127652	1	0.057	9.00E-165	8
RAB31	1.71E-168	0.612628017	0.36	0.004	2.67E-164	8
FCGR2A	3.19E-166	0.926556969	0.46	0.008	5.00E-162	8
BATF3	1.67E-160	0.648734475	0.28	0.002	2.63E-156	8
PPM1N	1.14E-157	1.330522424	0.72	0.026	1.78E-153	8
CD300LF	1.56E-145	0.53343948	0.26	0.002	2.44E-141	8
RNF144B	2.24E-145	0.818229322	0.36	0.005	3.51E-141	8
S100A8	6.17E-144	1.624988557	0.62	0.021	9.68E-140	8
ADGRE1	5.48E-143	0.805083989	0.42	0.008	8.59E-139	8
CAMK1	1.33E-140	0.767444848	0.46	0.01	2.08E-136	8
IFITM3	1.22E-138	3.14950733	1	0.073	1.92E-134	8
LST1	1.06E-133	3.713815859	1	0.078	1.67E-129	8
CD86	1.12E-132	0.803368936	0.46	0.011	1.76E-128	8
IL3RA	7.65E-126	0.503292118	0.26	0.003	1.20E-121	8
TYROBP	1.58E-114	3.137018335	1	0.094	2.48E-110	8
MPEG1	1.08E-109	0.972064658	0.54	0.021	1.69E-105	8
AIF1	2.59E-108	3.459377283	1	0.102	4.06E-104	8
DOCK5	1.50E-107	0.601556138	0.28	0.004	2.35E-103	8
CEBPA	5.90E-107	0.493849006	0.26	0.004	9.25E-103	8
FCGR3A	4.07E-103	2.390812576	1	0.106	6.38E-99	8
TYMP	2.75E-102	2.11930838	0.98	0.1	4.32E-98	8
WARS	1.23E-101	1.133973017	0.6	0.029	1.92E-97	8
SAMD4A	3.90E-99	0.433723128	0.26	0.004	6.11E-95	8

Table S2 Continued

Gene_marker	*P*_val	Avg_logFC	Pct.1	Pct.2	*P*_val_adj	Cluster
IRAK3	2.05E-98	0.628181066	0.36	0.009	3.21E-94	8
DUSP6	8.48E-92	0.868489884	0.56	0.028	1.33E-87	8
LILRB1	1.05E-88	1.123027018	0.6	0.034	1.64E-84	8
THEMIS2	2.75E-86	0.881475128	0.48	0.022	4.31E-82	8
PECAM1	4.16E-85	1.237113722	0.8	0.067	6.52E-81	8
P2RX1	4.54E-85	0.515193125	0.28	0.006	7.11E-81	8
LYN	4.00E-82	1.270752793	0.82	0.073	6.28E-78	8
SIGLEC10	7.22E-81	0.925535401	0.5	0.026	1.13E-76	8
CPPED1	1.03E-80	0.903456642	0.52	0.028	1.61E-76	8
ICAM4	4.57E-77	0.582025715	0.28	0.007	7.16E-73	8
SLC2A6	2.84E-75	0.882508114	0.5	0.028	4.44E-71	8
CDH23	8.76E-75	0.446398509	0.26	0.006	1.37E-70	8
RAB24	1.51E-72	0.800303667	0.6	0.041	2.37E-68	8
SCIMP	8.11E-71	0.745022891	0.4	0.018	1.27E-66	8
TNFSF13B	6.24E-66	0.648191017	0.42	0.022	9.78E-62	8
SOD2	8.66E-65	1.281383165	0.76	0.079	1.36E-60	8
LGALS3	1.11E-64	0.902181761	0.52	0.036	1.74E-60	8
GCA	7.04E-63	0.896023147	0.56	0.043	1.10E-58	8
ASAH1	1.20E-62	1.259915825	0.88	0.115	1.88E-58	8
TIMP1	1.80E-60	1.330983482	0.84	0.106	2.82E-56	8
ARRB1	1.88E-59	0.427030805	0.28	0.01	2.94E-55	8
RNF130	4.48E-59	1.016938286	0.66	0.063	7.02E-55	8
SLC7A7	9.97E-59	0.882320195	0.54	0.042	1.56E-54	8
TBXAS1	2.61E-58	0.789346606	0.46	0.031	4.09E-54	8
OAS1	7.42E-58	0.9523526	0.66	0.064	1.16E-53	8
BRI3	1.04E-57	1.114026269	0.68	0.07	1.62E-53	8
ATP6V1B2	1.67E-57	0.905392252	0.62	0.056	2.61E-53	8
NPC2	1.79E-57	1.769279211	0.98	0.194	2.80E-53	8
CUX1	4.23E-57	0.816940863	0.52	0.04	6.63E-53	8
BEST1	1.24E-56	0.808917725	0.56	0.046	1.95E-52	8

Table S2 Continued

Gene_marker	*P*_val	Avg_logFC	Pct.1	Pct.2	*P*_val_adj	Cluster
STX11	2.76E-55	0.927816637	0.5	0.039	4.33E-51	8
CEBPD	6.86E-55	0.804945808	0.38	0.022	1.08E-50	8
STXBP2	1.50E-52	1.734442856	0.9	0.168	2.35E-48	8
MAPKAPK3	1.98E-52	0.835943354	0.64	0.064	3.10E-48	8
NUP214	2.76E-52	0.75176873	0.48	0.037	4.33E-48	8
TKT	4.99E-52	1.518017518	0.9	0.158	7.82E-48	8
NAMPT	1.12E-50	0.802692516	0.48	0.038	1.76E-46	8
SMCO4	4.61E-49	0.87018692	0.6	0.061	7.22E-45	8
CYBB	1.00E-47	0.704511872	0.42	0.031	1.57E-43	8
TNFSF10	1.12E-47	1.334461738	0.76	0.114	1.76E-43	8
CEBPB	1.30E-47	1.350220261	0.94	0.19	2.04E-43	8
CTSS	1.62E-47	2.40944544	1	0.324	2.54E-43	8
ITGAX	8.44E-47	0.464706986	0.26	0.012	1.32E-42	8
UNC119	1.02E-43	0.911042182	0.58	0.064	1.60E-39	8
SULT1A1	1.99E-42	0.558933772	0.32	0.02	3.12E-38	8
RALB	2.24E-42	0.691440221	0.52	0.052	3.52E-38	8
SCPEP1	5.91E-42	0.569638996	0.38	0.029	9.27E-38	8
PSAP	1.23E-41	2.102174646	1	0.401	1.92E-37	8
MAP3K7CL	3.23E-41	0.627346682	0.26	0.014	5.06E-37	8
HHEX	3.23E-41	0.555177482	0.34	0.024	5.07E-37	8
S100A11	2.20E-40	2.006788942	1	0.411	3.44E-36	8
SAT1	8.06E-40	2.380784655	1	0.435	1.26E-35	8
C15orf39	1.28E-39	0.504515352	0.32	0.022	2.01E-35	8
ODF3B	6.98E-39	0.574366902	0.34	0.025	1.09E-34	8
C1orf162	8.81E-39	1.333235891	0.78	0.153	1.38E-34	8
HVCN1	1.37E-38	0.549692788	0.38	0.031	2.14E-34	8
LY96	9.51E-38	1.031228754	0.64	0.095	1.49E-33	8
BID	1.95E-37	0.996609214	0.66	0.098	3.06E-33	8
RIN3	3.40E-36	0.71815728	0.44	0.045	5.33E-32	8
ZFAND5	3.40E-36	1.131006142	0.76	0.148	5.33E-32	8

Table S2 Continued

Gene_marker	*P*_val	Avg_logFC	Pct.1	Pct.2	*P*_val_adj	Cluster
C20orf27	4.68E-36	0.755180729	0.52	0.062	7.34E-32	8
RGS2	1.77E-35	1.130006501	0.64	0.102	2.78E-31	8
ATG3	3.41E-34	1.084119802	0.68	0.118	5.35E-30	8
FTH1	4.57E-34	2.402480627	1	0.971	7.17E-30	8
FTL	4.73E-34	2.031734182	1	0.98	7.42E-30	8
FKBP1A	5.92E-33	1.200480973	0.98	0.355	9.29E-29	8
BLVRA	2.31E-32	0.732630607	0.46	0.054	3.63E-28	8
BLVRB	2.33E-31	0.505689319	0.34	0.031	3.65E-27	8
BCL2A1	4.23E-31	1.049520206	0.66	0.12	6.63E-27	8
TESC	4.57E-31	0.98310888	0.6	0.096	7.16E-27	8
MARCH1	8.70E-31	0.694631154	0.56	0.08	1.36E-26	8
S100A6	1.21E-30	1.596372859	1	0.843	1.90E-26	8
LGALS9	2.66E-30	0.85671542	0.68	0.121	4.18E-26	8
LGALS1	4.68E-30	1.480099062	1	0.437	7.33E-26	8
IFITM2	1.39E-29	1.586633872	0.98	0.59	2.18E-25	8
C10orf54	3.49E-29	1.252504808	0.86	0.242	5.47E-25	8
RARA	5.12E-29	0.623431119	0.38	0.041	8.03E-25	8
SNX27	8.58E-29	0.605829724	0.4	0.046	1.35E-24	8
CD300A	9.75E-29	0.677404343	0.44	0.055	1.53E-24	8
TNFRSF1B	2.59E-28	1.036229939	0.68	0.142	4.06E-24	8
IFNGR1	6.87E-28	0.63579641	0.5	0.07	1.08E-23	8
S100A4	6.95E-28	1.283928319	1	0.872	1.09E-23	8
RHOC	1.16E-27	1.052749458	0.76	0.177	1.81E-23	8
NAPRT	1.33E-27	0.511939014	0.34	0.035	2.09E-23	8
NADK	3.37E-27	0.630757253	0.42	0.053	5.28E-23	8
CD55	4.95E-27	0.643552947	0.5	0.073	7.76E-23	8
SDCBP	7.59E-27	0.891509301	0.74	0.157	1.19E-22	8
C9orf72	8.13E-27	0.578675131	0.32	0.032	1.27E-22	8
UBXN11	1.04E-26	0.630274595	0.48	0.068	1.64E-22	8
COTL1	3.07E-26	1.086102857	1	0.679	4.81E-22	8

Table S2 Continued

Gene_marker	*P*_val	Avg_logFC	Pct.1	Pct.2	*P*_val_adj	Cluster
IFNGR2	4.04E-26	0.680855783	0.4	0.052	6.33E-22	8
VMP1	9.86E-26	1.070509468	0.78	0.197	1.54E-21	8
EVI2B	1.02E-25	1.279423535	0.98	0.44	1.60E-21	8
MSRB1	1.11E-25	0.582725998	0.4	0.051	1.74E-21	8
FAM49A	1.88E-25	0.514193276	0.32	0.034	2.95E-21	8
AP1S2	2.97E-25	0.91133967	0.72	0.161	4.66E-21	8
OAZ1	4.25E-25	0.887656228	1	0.901	6.67E-21	8
GRN	8.44E-25	0.729660929	0.64	0.126	1.32E-20	8
PYCARD	8.70E-25	1.108458716	0.9	0.292	1.36E-20	8
CARD16	2.30E-24	1.152908908	0.92	0.335	3.60E-20	8
NUMB	3.11E-24	0.610796469	0.46	0.069	4.87E-20	8
PAK1	3.26E-24	0.456712787	0.28	0.028	5.11E-20	8
SNX10	4.18E-24	0.79911623	0.52	0.092	6.55E-20	8
UBE2D1	4.57E-24	0.721756106	0.52	0.089	7.16E-20	8
FAM45A	8.01E-24	0.592286905	0.56	0.097	1.26E-19	8
DPEP2	1.31E-23	0.556108541	0.34	0.041	2.06E-19	8
CAPZA2	3.42E-23	0.852405342	0.88	0.259	5.36E-19	8
TSPO	5.51E-23	1.06884555	0.88	0.328	8.64E-19	8
ARAP1	1.13E-22	0.480864996	0.36	0.046	1.78E-18	8
NUDT16	1.21E-22	0.499709996	0.3	0.034	1.90E-18	8
NAGK	2.87E-22	0.668971175	0.44	0.071	4.50E-18	8
ARL4A	4.99E-22	0.638857851	0.46	0.076	7.82E-18	8
ANXA5	7.20E-22	0.750200383	0.82	0.221	1.13E-17	8
CASP1	9.14E-22	0.982400087	0.76	0.209	1.43E-17	8
HLA-DPA1	1.71E-21	1.029041056	1	0.681	2.69E-17	8
RNH1	1.79E-21	0.856405451	0.78	0.222	2.80E-17	8
PRKCD	1.91E-21	0.470871084	0.3	0.035	2.99E-17	8
ARRB2	2.18E-21	0.865382246	0.84	0.247	3.42E-17	8
VAMP5	2.37E-21	1.071662008	0.84	0.295	3.72E-17	8
GNGT2	2.79E-21	0.418803904	0.32	0.039	4.38E-17	8

Table S2 Continued

Gene_marker	*P*_val	Avg_logFC	Pct.1	Pct.2	*P*_val_adj	Cluster
NEAT1	4.90E-21	1.088630499	1	0.64	7.69E-17	8
RTN3	8.84E-21	0.563494768	0.44	0.072	1.39E-16	8
JAML	8.94E-21	0.625899577	0.42	0.068	1.40E-16	8
VASP	9.14E-21	0.918792429	0.76	0.213	1.43E-16	8
CD4	2.33E-20	0.459864791	0.38	0.056	3.66E-16	8
LTA4H	2.75E-20	0.811727219	0.56	0.118	4.32E-16	8
GLUL	3.30E-20	0.827517237	0.5	0.097	5.18E-16	8
MBD2	4.06E-20	0.405446052	0.3	0.037	6.36E-16	8
FAM110A	9.04E-20	0.588218574	0.32	0.043	1.42E-15	8
NFKBIZ	1.30E-19	0.584240621	0.48	0.089	2.04E-15	8
SLC1A5	1.51E-19	0.395754036	0.26	0.029	2.37E-15	8
FGR	1.53E-19	0.818320377	0.7	0.184	2.40E-15	8
RNPEP	2.05E-19	0.489296066	0.34	0.048	3.21E-15	8
DUSP1	2.19E-19	0.955371745	1	0.734	3.43E-15	8
LYL1	4.13E-19	0.66480188	0.38	0.062	6.48E-15	8
NAP1L1	5.33E-19	0.951224388	1	0.575	8.36E-15	8
RELT	9.58E-19	0.424939747	0.3	0.039	1.50E-14	8
SNX18	1.05E-18	0.596084137	0.36	0.056	1.64E-14	8
HLA-DRA	1.19E-18	0.944127206	1	0.614	1.86E-14	8
MTSS1	1.49E-18	0.595655714	0.38	0.062	2.34E-14	8
MT2A	1.70E-18	1.412777551	0.92	0.468	2.67E-14	8
ALOX5	1.71E-18	0.535621596	0.42	0.072	2.69E-14	8
PPT1	2.04E-18	0.654342516	0.56	0.125	3.19E-14	8
CALM2	3.05E-18	0.911842072	0.98	0.714	4.78E-14	8
UBE2J1	3.77E-18	0.605901203	0.58	0.131	5.91E-14	8
NINJ1	3.97E-18	0.572229682	0.42	0.074	6.22E-14	8
ATP6V0D1	4.01E-18	0.602236768	0.54	0.117	6.28E-14	8
RNF13	4.20E-18	0.759704851	0.5	0.108	6.59E-14	8
MT-CO1	1.59E-17	0.522470568	1	0.998	2.49E-13	8
TCIRG1	1.87E-17	0.679347184	0.58	0.142	2.93E-13	8

Table S2 Continued

Gene_marker	*P*_val	Avg_logFC	Pct.1	Pct.2	*P*_val_adj	Cluster
BTK	2.12E-17	0.521880518	0.4	0.072	3.32E-13	8
HOTAIRM1	2.21E-17	0.482527865	0.36	0.058	3.47E-13	8
ZYX	2.50E-17	0.646003736	0.66	0.17	3.92E-13	8
IMPDH1	2.82E-17	0.511248597	0.44	0.083	4.42E-13	8
PGLS	2.86E-17	0.692405357	0.68	0.182	4.49E-13	8
NANS	5.10E-17	0.445440138	0.32	0.049	7.99E-13	8
H3F3A	9.13E-17	0.68404998	0.98	0.882	1.43E-12	8
DRAP1	9.84E-17	0.766733154	0.96	0.475	1.54E-12	8
VIM	1.55E-16	0.807906408	0.98	0.563	2.44E-12	8
AGTRAP	1.62E-16	0.684911806	0.5	0.109	2.54E-12	8
RPS9	3.77E-16	0.446450038	1	0.995	5.91E-12	8
APLP2	3.92E-16	0.542933814	0.44	0.089	6.14E-12	8
SERTAD3	4.34E-16	0.321101518	0.26	0.035	6.80E-12	8
SNAP23	6.45E-16	0.639300847	0.64	0.168	1.01E-11	8
RNF149	8.92E-16	0.655855157	0.68	0.193	1.40E-11	8
ANXA2	9.42E-16	0.851478894	0.76	0.271	1.48E-11	8
GPX1	1.53E-15	0.972518421	0.82	0.323	2.41E-11	8
SNX2	1.75E-15	0.589760983	0.48	0.107	2.75E-11	8
POU2F2	1.86E-15	0.762772322	0.54	0.144	2.92E-11	8
ATOX1	2.25E-15	0.632117025	0.54	0.133	3.53E-11	8
LINC00936	2.37E-15	0.697959831	0.42	0.089	3.72E-11	8
HSBP1	2.63E-15	0.758361698	0.56	0.153	4.12E-11	8
CNIH4	3.40E-15	0.584619913	0.54	0.13	5.33E-11	8
UNC93B1	3.64E-15	0.485852976	0.3	0.048	5.71E-11	8
RGS19	3.96E-15	0.753303382	0.72	0.228	6.21E-11	8
EIF4EBP1	4.34E-15	0.46127398	0.28	0.043	6.81E-11	8
RHOG	4.77E-15	0.744443819	0.78	0.267	7.47E-11	8
CDC42EP3	5.76E-15	0.559954221	0.6	0.152	9.02E-11	8
HN1	8.08E-15	0.666683238	0.78	0.249	1.27E-10	8
NOTCH2	9.95E-15	0.43594245	0.26	0.038	1.56E-10	8

Table S2 Continued

Gene_marker	*P*_val	Avg_logFC	Pct.1	Pct.2	*P*_val_adj	Cluster
SGPP1	1.31E-14	0.387379002	0.28	0.043	2.05E-10	8
TALDO1	1.40E-14	0.741144033	0.68	0.219	2.19E-10	8
RNASET2	1.58E-14	0.783470356	0.9	0.422	2.48E-10	8
PABPC4	1.88E-14	0.60058448	0.58	0.153	2.94E-10	8
MEF2C	2.64E-14	0.587556736	0.36	0.073	4.14E-10	8
CYBA	2.92E-14	0.549819073	1	0.926	4.58E-10	8
OAZ2	3.12E-14	0.517568708	0.46	0.104	4.90E-10	8
PGK1	3.55E-14	0.738448475	0.86	0.343	5.56E-10	8
PTPN6	3.68E-14	0.734806828	0.76	0.283	5.77E-10	8
FCGRT	3.69E-14	0.677019897	0.5	0.128	5.79E-10	8
GDI2	4.04E-14	0.761335669	0.82	0.404	6.34E-10	8
AOAH	4.52E-14	0.411282928	0.4	0.08	7.08E-10	8
SCO2	7.30E-14	0.371656764	0.26	0.04	1.14E-09	8
ATP6V0B	8.50E-14	0.693411994	0.84	0.317	1.33E-09	8
ATP1B3	1.19E-13	0.582156299	0.62	0.181	1.87E-09	8
ARPC3	1.23E-13	0.647134665	1	0.733	1.93E-09	8
GBP2	1.27E-13	0.609678377	0.68	0.207	1.99E-09	8
CTSZ	3.45E-13	0.402368109	0.3	0.054	5.41E-09	8
CSTB	3.49E-13	0.825114307	0.86	0.473	5.47E-09	8
LAPTM4A	4.15E-13	0.590966986	0.8	0.276	6.51E-09	8
CX3CR1	9.03E-13	0.493339463	0.44	0.104	1.42E-08	8
WSB1	1.26E-12	0.765471525	0.64	0.223	1.97E-08	8
MTPN	1.26E-12	0.462274653	0.56	0.148	1.98E-08	8
CACUL1	1.46E-12	0.402431871	0.36	0.075	2.28E-08	8
TMSB4X	1.89E-12	0.277221408	1	1	2.97E-08	8
ZFP36	2.09E-12	0.804047001	0.88	0.426	3.27E-08	8
NOP10	2.09E-12	0.706339608	0.84	0.388	3.28E-08	8
NAAA	2.60E-12	0.64571223	0.4	0.097	4.07E-08	8
LAMTOR4	2.72E-12	0.677319874	0.94	0.48	4.26E-08	8
HLA-DRB1	3.31E-12	0.671248131	0.96	0.655	5.18E-08	8

Table S2 Continued

Gene_marker	*P*_val	Avg_logFC	Pct.1	Pct.2	*P*_val_adj	Cluster
ABI3	3.62E-12	0.669688651	0.6	0.199	5.68E-08	8
INSIG1	3.94E-12	0.487935261	0.42	0.1	6.18E-08	8
NACA	4.04E-12	0.49208631	1	0.909	6.34E-08	8
SLC25A6	4.30E-12	0.532329483	1	0.84	6.75E-08	8
CMTM6	4.55E-12	0.535207231	0.66	0.204	7.14E-08	8
ARPC1B	5.09E-12	0.521373049	0.98	0.702	7.98E-08	8
PRELID1	6.39E-12	0.686972542	0.8	0.36	1.00E-07	8
DOCK2	6.94E-12	0.397886726	0.36	0.078	1.09E-07	8
GABARAP	7.93E-12	0.653525604	0.8	0.346	1.24E-07	8
ITM2B	8.91E-12	0.662407391	0.98	0.829	1.40E-07	8
YBX1	9.00E-12	0.571360953	1	0.805	1.41E-07	8
STX10	9.36E-12	0.470411785	0.52	0.142	1.47E-07	8
GNG5	1.56E-11	0.652388586	0.78	0.329	2.45E-07	8
ACTB	1.73E-11	0.490301291	1	1	2.71E-07	8
H2AFY	1.75E-11	0.543675616	0.66	0.228	2.74E-07	8
LEPROT	1.88E-11	0.47453175	0.38	0.09	2.94E-07	8
ZNF106	1.91E-11	0.36567573	0.36	0.079	2.99E-07	8
ANXA4	2.00E-11	0.399621851	0.28	0.054	3.14E-07	8
ADRBK1	2.05E-11	0.434697148	0.4	0.096	3.22E-07	8
TMSB10	2.29E-11	0.344070047	1	1	3.59E-07	8
ZEB2	2.30E-11	0.522990233	0.7	0.251	3.61E-07	8
ACP5	2.43E-11	0.541215025	0.5	0.143	3.81E-07	8
RBX1	2.44E-11	0.57159991	0.84	0.342	3.82E-07	8
SYNGR2	4.91E-11	0.609514589	0.6	0.204	7.70E-07	8
CECR1	5.54E-11	0.404372717	0.34	0.077	8.68E-07	8
C11orf21	5.87E-11	0.470981267	0.38	0.092	9.20E-07	8
ESRRA	7.88E-11	0.348856506	0.32	0.069	1.23E-06	8
CTSB	8.08E-11	0.541044202	0.44	0.123	1.27E-06	8
LAIR1	8.55E-11	0.3424936	0.28	0.055	1.34E-06	8
CTSD	8.91E-11	0.606412457	0.66	0.245	1.40E-06	8

Table S2 Continued

Gene_marker	*P*_val	Avg_logFC	Pct.1	Pct.2	*P*_val_adj	Cluster
TUBA1B	9.55E-11	0.615468567	0.8	0.373	1.50E-06	8
HLA-DQA2	1.03E-10	0.725772886	0.82	0.383	1.62E-06	8
VPS29	1.15E-10	0.534186344	0.64	0.226	1.80E-06	8
CARD19	1.29E-10	0.372016318	0.28	0.056	2.02E-06	8
GCH1	1.77E-10	0.44626444	0.32	0.073	2.78E-06	8
DOK2	1.85E-10	0.575420816	0.82	0.325	2.90E-06	8
RHOB	1.90E-10	0.451051181	0.42	0.114	2.97E-06	8
RPS19	1.97E-10	0.273806962	1	1	3.09E-06	8
CNPY3	2.00E-10	0.524629255	0.62	0.219	3.13E-06	8
MYL6	2.21E-10	0.464767998	1	0.933	3.46E-06	8
MGAT1	2.30E-10	0.413381532	0.48	0.136	3.60E-06	8
ADA	2.67E-10	0.450735946	0.52	0.156	4.19E-06	8
SAMHD1	2.79E-10	0.469320256	0.52	0.155	4.38E-06	8
PIK3AP1	2.85E-10	0.420817924	0.3	0.066	4.46E-06	8
CD74	2.96E-10	0.440648127	1	0.938	4.64E-06	8
RPP25L	3.02E-10	0.334971318	0.3	0.065	4.73E-06	8
ICAM2	3.71E-10	0.54647426	0.5	0.155	5.81E-06	8
ARHGAP27	3.72E-10	0.43405006	0.28	0.06	5.83E-06	8
PQLC3	4.13E-10	0.513229785	0.5	0.154	6.48E-06	8
FOS	4.42E-10	0.807143685	0.86	0.473	6.94E-06	8
CTSH	4.88E-10	0.385509284	0.34	0.081	7.64E-06	8
AP2S1	5.01E-10	0.603823649	0.7	0.277	7.86E-06	8
ID2	5.29E-10	0.630673386	0.84	0.433	8.29E-06	8
EFHD2	5.61E-10	0.419938193	0.54	0.16	8.79E-06	8
LAP3	6.29E-10	0.389477068	0.28	0.061	9.86E-06	8
LAPTM5	7.57E-10	0.500334906	0.96	0.705	1.19E-05	8
HLA-DPB1	8.21E-10	0.580807353	0.98	0.779	1.29E-05	8
RAB10	8.25E-10	0.587078206	0.5	0.169	1.29E-05	8
SKAP2	8.35E-10	0.38236173	0.3	0.068	1.31E-05	8
LYST	8.42E-10	0.461249356	0.4	0.109	1.32E-05	8

Table S2 Continued

Gene_marker	*P*_val	Avg_logFC	Pct.1	Pct.2	*P*_val_adj	Cluster
PSMA4	8.67E-10	0.597439316	0.86	0.436	1.36E-05	8
HLA-DQA1	1.21E-09	0.516028862	0.74	0.29	1.90E-05	8
EIF4E2	1.35E-09	0.401479888	0.46	0.133	2.12E-05	8
NRBF2	1.53E-09	0.364660863	0.32	0.076	2.40E-05	8
GRINA	2.43E-09	0.336140147	0.26	0.055	3.81E-05	8
UPP1	2.90E-09	0.282604687	0.34	0.083	4.55E-05	8
ARPC5	3.08E-09	0.611100768	0.88	0.517	4.83E-05	8
CD48	3.21E-09	0.578098124	0.86	0.532	5.03E-05	8
ATG16L2	3.51E-09	0.362177621	0.3	0.071	5.50E-05	8
AP2A1	3.58E-09	0.386003182	0.28	0.064	5.62E-05	8
GLRX	3.73E-09	0.536739969	0.56	0.203	5.85E-05	8
SLIRP	4.17E-09	0.468499989	0.62	0.224	6.54E-05	8
TUBA1A	4.29E-09	0.574824351	0.72	0.323	6.72E-05	8
CYSLTR1	4.39E-09	0.259710326	0.28	0.062	6.88E-05	8
GSTO1	4.44E-09	0.512900464	0.54	0.185	6.96E-05	8
ITGB2	4.80E-09	0.527872042	1	0.668	7.53E-05	8
TLE4	4.84E-09	0.373781788	0.42	0.119	7.59E-05	8
BCCIP	4.94E-09	0.265218184	0.26	0.056	7.74E-05	8
PLEKHO1	5.57E-09	0.399983965	0.26	0.058	8.72E-05	8
CAPNS1	5.80E-09	0.387282799	0.38	0.106	9.09E-05	8
FAM96A	5.98E-09	0.443687528	0.46	0.143	9.38E-05	8
PTEN	6.54E-09	0.306218803	0.32	0.078	0.000102507	8
MT-ATP6	6.84E-09	0.467936472	1	0.901	0.000107258	8
MYD88	6.98E-09	0.407390008	0.32	0.082	0.00010938	8
RABGAP1L	7.63E-09	0.441944873	0.46	0.143	0.000119654	8
CTSC	7.77E-09	0.536359172	0.8	0.375	0.000121878	8
ENY2	7.91E-09	0.48851061	0.64	0.244	0.000123943	8
GRB2	9.43E-09	0.44137146	0.58	0.207	0.000147798	8
LAMP2	9.87E-09	0.386821876	0.34	0.089	0.000154682	8
GMFG	9.94E-09	0.47310163	0.98	0.697	0.000155884	8

Table S2 Continued

Gene_marker	*P*_val	Avg_logFC	Pct.1	Pct.2	*P*_val_adj	Cluster
EPSTI1	1.11E-08	0.418003028	0.38	0.109	0.000174761	8
SMAP2	1.16E-08	0.363978133	0.38	0.107	0.000181989	8
SNX3	1.21E-08	0.510592076	0.84	0.455	0.000190431	8
PSMC2	1.23E-08	0.312172839	0.38	0.104	0.000193175	8
SERF2	1.24E-08	0.355955757	1	0.967	0.000194178	8
VNN2	1.59E-08	0.431349921	0.36	0.102	0.000248759	8
DNAJB12	1.80E-08	0.257535841	0.26	0.058	0.000282261	8
CHCHD10	2.54E-08	0.483845583	0.6	0.234	0.000397541	8
MYADM	2.54E-08	0.424758966	0.3	0.078	0.000398578	8
BLOC1S1	2.60E-08	0.565342665	0.8	0.402	0.000407989	8
SHKBP1	2.62E-08	0.446623727	0.54	0.19	0.000411186	8
RAC1	2.75E-08	0.51062693	0.78	0.363	0.000430782	8
SRA1	2.89E-08	0.322455538	0.3	0.076	0.000453038	8
AGPAT2	3.48E-08	0.337215869	0.28	0.069	0.000545332	8
VAMP8	3.56E-08	0.551241137	0.8	0.437	0.000557897	8
HDAC5	3.81E-08	0.294731576	0.28	0.068	0.000596604	8
SRGN	4.36E-08	0.476090101	0.96	0.707	0.000683211	8
EPN1	5.42E-08	0.343921805	0.28	0.07	0.000849989	8
ASCL2	5.53E-08	0.354211132	0.28	0.07	0.000867145	8
UBE2R2	5.91E-08	0.361336802	0.28	0.072	0.000927008	8
SERP1	5.94E-08	0.589907086	0.84	0.49	0.000931351	8
COX5B	6.43E-08	0.47585461	0.98	0.617	0.001007523	8
TPI1	6.63E-08	0.533305381	0.88	0.474	0.001039801	8
CBWD1	7.67E-08	0.262152418	0.28	0.07	0.001202391	8
ZNF706	7.72E-08	0.457064439	0.66	0.268	0.001210235	8
MOB1A	8.93E-08	0.463598387	0.64	0.252	0.001399958	8
HAGH	9.02E-08	0.35073274	0.42	0.133	0.001413675	8
OSTF1	9.75E-08	0.450270099	0.72	0.309	0.001528616	8
OSBPL8	9.99E-08	0.266712055	0.36	0.102	0.00156606	8
POLE4	1.06E-07	0.473032263	0.48	0.171	0.00166052	8

Table S2 Continued

Gene_marker	*P*_val	Avg_logFC	Pct.1	Pct.2	*P*_val_adj	Cluster
ERP44	1.11E-07	0.32759855	0.5	0.168	0.001736801	8
ADAM10	1.25E-07	0.3460711	0.36	0.105	0.00195667	8
SPINT2	1.25E-07	0.337969473	0.32	0.091	0.001959672	8
SSH2	1.43E-07	0.308136494	0.3	0.08	0.00223823	8
MT-ND1	1.50E-07	0.435973053	0.96	0.795	0.002357446	8
PLSCR1	1.57E-07	0.361535943	0.26	0.066	0.002454809	8
MT-CYB	1.62E-07	0.33810049	1	0.964	0.002539839	8
ATP2B1	1.73E-07	0.312960185	0.4	0.123	0.002710346	8
POLD4	1.83E-07	0.453376042	0.54	0.204	0.002864157	8
COX8A	2.06E-07	0.533877732	0.84	0.488	0.003232028	8
SMS	2.26E-07	0.444630619	0.5	0.19	0.003549139	8
GPR65	2.42E-07	0.461128752	0.46	0.166	0.003789736	8
SH3BP2	2.72E-07	0.33825055	0.3	0.083	0.004256449	8
NMI	2.93E-07	0.340925166	0.34	0.1	0.004590302	8
UTRN	3.24E-07	0.303860401	0.38	0.118	0.005086081	8
PLIN2	3.61E-07	0.289727272	0.28	0.076	0.005664355	8
ATP5E	3.68E-07	0.302194048	1	0.976	0.005765681	8
MT-ND4L	3.72E-07	0.50603249	0.68	0.327	0.005823819	8
HIGD2A	3.86E-07	0.474346902	0.76	0.447	0.006046175	8
CCPG1	4.72E-07	0.316908688	0.26	0.069	0.007392702	8
NAPA	4.74E-07	0.300086472	0.48	0.164	0.007425334	8
LSP1	4.86E-07	0.468986388	0.94	0.675	0.007613708	8
MT-CO3	5.15E-07	0.268293024	1	0.994	0.008071474	8
NR1H2	5.86E-07	0.325862599	0.36	0.112	0.009190026	8
ERICH1	6.66E-07	0.39075597	0.5	0.183	0.010437248	8
HSD17B11	7.05E-07	0.389563247	0.62	0.247	0.011047114	8
CTNNBL1	9.51E-07	0.274591347	0.28	0.078	0.01490554	8
IL10RB	9.60E-07	0.335273748	0.34	0.107	0.015052988	8
UQCRC1	1.00E-06	0.364019716	0.44	0.156	0.015690696	8
ARPC2	1.01E-06	0.372178801	0.98	0.802	0.015780264	8

Table S2 Continued

Gene_marker	*P*_val	Avg_logFC	Pct.1	Pct.2	*P*_val_adj	Cluster
HK1	1.12E-06	0.297172217	0.26	0.071	0.017523559	8
EIF1	1.19E-06	0.258584735	1	0.994	0.018652705	8
CDC42	1.23E-06	0.444075811	0.96	0.701	0.019263988	8
CDKN2D	1.29E-06	0.286697323	0.44	0.152	0.0201724	8
EIF6	1.32E-06	0.352733059	0.52	0.193	0.02068116	8
REEP5	1.47E-06	0.463357098	0.56	0.251	0.023027649	8
GSTP1	1.57E-06	0.473640039	0.86	0.528	0.02460639	8
NDUFS6	1.73E-06	0.419145292	0.62	0.275	0.027101984	8
CTSA	1.82E-06	0.277366408	0.36	0.117	0.028564087	8
ALDOA	1.82E-06	0.459249235	0.88	0.678	0.028600927	8
GNAI2	1.96E-06	0.330223223	0.46	0.168	0.030666462	8
WDR83OS	2.10E-06	0.452455358	0.78	0.418	0.032877569	8
TCEB2	2.17E-06	0.447471327	0.94	0.697	0.03406016	8
YWHAE	2.26E-06	0.369717679	0.54	0.214	0.035443747	8
ARRDC1	2.32E-06	0.32228957	0.42	0.15	0.036311874	8
WAS	2.61E-06	0.367484162	0.62	0.257	0.040968047	8
CHP1	2.71E-06	0.34123758	0.26	0.075	0.042467797	8
IRF7	2.74E-06	0.373132483	0.34	0.114	0.042962784	8
GLIPR1	2.89E-06	0.574582547	0.5	0.228	0.045289849	8
HMGN2	2.91E-06	0.400694789	0.94	0.668	0.045575251	8
LRRFIP1	3.10E-06	0.405306638	0.74	0.361	0.048644854	8
TMEM167A	3.42E-06	0.30604365	0.46	0.171	0.053599998	8
GYG1	3.71E-06	0.32321792	0.38	0.132	0.058203913	8
MAP3K11	3.76E-06	0.313376011	0.26	0.076	0.05893236	8
PFKL	3.80E-06	0.271637075	0.4	0.138	0.059579762	8
MIIP	3.85E-06	0.277751767	0.28	0.083	0.060404345	8
SH3BGRL	3.90E-06	0.481678961	0.76	0.392	0.061074697	8
ACAA1	4.27E-06	0.3533854	0.4	0.146	0.066914517	8
AKR1A1	4.51E-06	0.29455062	0.42	0.152	0.070658525	8
WASF2	4.54E-06	0.302702692	0.68	0.293	0.071121292	8

Table S2 Continued

Gene_marker	*P*_val	Avg_logFC	Pct.1	Pct.2	*P*_val_adj	Cluster
GNB2	4.61E-06	0.441092884	0.64	0.298	0.072283545	8
MX2	5.40E-06	0.253489258	0.26	0.075	0.084681609	8
NARS	5.42E-06	0.250219741	0.36	0.12	0.084984017	8
ACTR2	6.13E-06	0.336514128	0.74	0.335	0.096123203	8
SQRDL	6.20E-06	0.349023225	0.34	0.114	0.097122537	8
C1orf122	6.55E-06	0.254257165	0.3	0.094	0.102607867	8
ITGB2-AS1	7.00E-06	0.272275489	0.4	0.142	0.109680085	8
DNAJA1	7.11E-06	0.313132062	0.56	0.223	0.111415384	8
COX5A	7.25E-06	0.362208853	0.68	0.332	0.113636772	8
PSME2	7.45E-06	0.412637388	0.88	0.587	0.116786398	8
FAM32A	7.91E-06	0.325413254	0.42	0.156	0.124062643	8
POMP	8.12E-06	0.510358854	0.74	0.446	0.127324994	8
RTN4	8.83E-06	0.330529582	0.64	0.289	0.138397503	8
LAMTOR2	9.07E-06	0.356374746	0.54	0.225	0.142119552	8
ZFAS1	9.58E-06	0.34724812	0.82	0.42	0.150213113	8
USP15	9.65E-06	0.357312722	0.44	0.173	0.15121291	8
LY6E	9.68E-06	0.457169336	0.9	0.619	0.151698086	8
CD37	9.70E-06	0.334829898	1	0.726	0.152099796	8
RPS27L	1.06E-05	0.505677003	0.8	0.458	0.166483303	8
PFDN5	1.15E-05	0.280519679	1	0.96	0.17953964	8
PABPC1	1.16E-05	0.293106958	0.98	0.908	0.181447949	8
CCT5	1.16E-05	0.361714599	0.34	0.121	0.181638161	8
PRDX3	1.16E-05	0.273051395	0.38	0.137	0.182047916	8
JUNB	1.20E-05	0.48362366	0.94	0.729	0.187514949	8
PGAM1	1.22E-05	0.330885833	0.64	0.295	0.191181598	8
ATP5G2	1.30E-05	0.395291678	0.96	0.788	0.203429195	8
MPC1	1.37E-05	0.313469802	0.3	0.1	0.215515333	8
RAB5C	1.44E-05	0.393616135	0.62	0.292	0.226326705	8
AKIRIN2	1.50E-05	0.288689274	0.32	0.109	0.235243945	8
VPS28	1.52E-05	0.341178254	0.72	0.336	0.237958921	8

Table S2 Continued

Gene_marker	*P*_val	Avg_logFC	Pct.1	Pct.2	*P*_val_adj	Cluster
CALHM2	1.73E-05	0.267844476	0.26	0.08	0.271762026	8
FXYD5	1.88E-05	0.313997888	0.96	0.603	0.295173744	8
TMBIM4	1.99E-05	0.391267896	0.7	0.366	0.312107062	8
S100A10	2.00E-05	0.402000605	0.92	0.597	0.31387085	8
C7orf50	2.02E-05	0.326333334	0.44	0.174	0.316644633	8
FBXL5	2.12E-05	0.362462934	0.32	0.115	0.332626943	8
MT-ND2	2.33E-05	0.274847628	1	0.98	0.365216166	8
GPR155	2.41E-05	0.267860033	0.26	0.082	0.377889906	8
NCOA4	2.49E-05	0.290211264	0.36	0.132	0.39104019	8
ADI1	2.52E-05	0.322068887	0.32	0.116	0.395294366	8
CHMP5	2.73E-05	0.258428864	0.46	0.183	0.427741158	8
NAA38	2.90E-05	0.308193244	0.68	0.32	0.455307412	8
H2AFZ	2.98E-05	0.441648599	0.78	0.479	0.467491042	8
MRPL23	3.00E-05	0.264224746	0.38	0.143	0.470635202	8
MAP2K3	3.09E-05	0.280915173	0.3	0.102	0.484634054	8
TIMM8B	3.17E-05	0.319218658	0.38	0.149	0.496569801	8
C1orf43	3.23E-05	0.27227352	0.44	0.176	0.507024368	8
POLR1D	3.37E-05	0.255393895	0.58	0.246	0.528383863	8
ADGRE5	3.42E-05	0.413562115	0.42	0.179	0.536758477	8
CFL1	3.45E-05	0.256717833	1	0.961	0.540863646	8
SUPT4H1	3.50E-05	0.410975038	0.58	0.272	0.548527171	8
ATP5J2	3.54E-05	0.420392622	0.82	0.555	0.554847953	8
KLF3	4.07E-05	0.298772565	0.3	0.106	0.637836783	8
SERPINB1	4.38E-05	0.251212776	0.52	0.217	0.687179192	8
PSMB10	4.39E-05	0.312172591	0.54	0.241	0.688701086	8
FAM192A	4.40E-05	0.263939094	0.36	0.135	0.689051251	8
YWHAH	4.50E-05	0.353773624	0.44	0.184	0.705221206	8
ATP5C1	4.50E-05	0.364131122	0.64	0.301	0.706142489	8
SLC25A5	4.89E-05	0.453078452	0.64	0.339	0.766384085	8
NDUFS7	5.45E-05	0.264564581	0.68	0.31	0.854928073	8

Table S2 Continued

Gene_marker	*P*_val	Avg_logFC	Pct.1	Pct.2	*P*_val_adj	Cluster
PRR13	5.88E-05	0.331119572	0.88	0.577	0.922060336	8
DNASE2	6.03E-05	0.301736536	0.32	0.119	0.945872163	8
COX6B1	6.35E-05	0.357823002	0.96	0.682	0.995387771	8
NDUFAF3	6.59E-05	0.369959409	0.46	0.217	1	8
EMP3	6.73E-05	0.347854347	0.9	0.688	1	8
PPDPF	7.16E-05	0.322885234	0.88	0.595	1	8
CD44	7.49E-05	0.286129179	0.78	0.417	1	8
IQGAP1	7.64E-05	0.349773646	0.54	0.244	1	8
C4orf48	8.06E-05	0.330082511	0.54	0.247	1	8
PSMB9	8.11E-05	0.342212918	0.9	0.685	1	8
C14orf2	8.20E-05	0.316889911	0.94	0.73	1	8
DYNLL1	8.54E-05	0.417596101	0.76	0.434	1	8
VPS35	8.59E-05	0.315973115	0.34	0.133	1	8
SEC11A	8.60E-05	0.364599067	0.62	0.313	1	8
PSMB3	9.08E-05	0.309125253	0.74	0.423	1	8
NDUFB5	9.33E-05	0.275952616	0.52	0.234	1	8
COX7B	9.42E-05	0.334397412	0.8	0.442	1	8
ATP5L	9.77E-05	0.288455544	0.98	0.85	1	8
ATP1A1	0.00011633	0.355989209	0.46	0.212	1	8
RP5-1171I10.5	0.000116991	0.329658227	0.58	0.302	1	8
MRPS18C	0.000124826	0.271802772	0.38	0.156	1	8
NDUFA6	0.00012818	0.331229882	0.56	0.275	1	8
DYNLT1	0.000132233	0.281717981	0.42	0.18	1	8
DDX21	0.000140198	0.268851312	0.44	0.19	1	8
HLA-B	0.000150099	0.253637499	1	0.911	1	8
VTI1B	0.000165005	0.276279214	0.36	0.147	1	8
VMA21	0.000165175	0.260104798	0.28	0.103	1	8
PDCD6IP	0.000184123	0.25016734	0.26	0.093	1	8
CAPZA1	0.000198288	0.417556491	0.7	0.382	1	8
ERGIC3	0.000206932	0.278781973	0.56	0.272	1	8

Table S2 Continued

Gene_marker	*P*_val	Avg_logFC	Pct.1	Pct.2	*P*_val_adj	Cluster
AURKAIP1	0.000218149	0.294256665	0.6	0.307	1	8
IRF1	0.000227986	0.336164766	0.68	0.392	1	8
ATP6AP2	0.000230337	0.336423514	0.58	0.286	1	8
VAPA	0.000232365	0.282650652	0.68	0.333	1	8
HLA-DQB1	0.000234346	0.276119946	0.58	0.284	1	8
M6PR	0.00024382	0.267506884	0.58	0.281	1	8
LRPAP1	0.000244509	0.304636658	0.44	0.194	1	8
IRF2	0.000256783	0.281671628	0.36	0.153	1	8
GPX4	0.00025765	0.277974551	0.78	0.406	1	8
TMEM179B	0.000280817	0.337451397	0.4	0.178	1	8
EIF1B	0.000306579	0.325769727	0.52	0.261	1	8
MYO1G	0.00031896	0.373304868	0.64	0.341	1	8
DBNL	0.000345286	0.267135144	0.54	0.255	1	8
CYTIP	0.00034864	0.273477404	0.46	0.216	1	8
MINOS1	0.000363874	0.298198734	0.68	0.351	1	8
RHOA	0.00038178	0.346325232	0.82	0.54	1	8
RAP1B	0.000399521	0.25321701	0.88	0.518	1	8
CSK	0.00041578	0.257916881	0.56	0.265	1	8
COX7A2	0.00042972	0.290370052	0.96	0.69	1	8
KDELR2	0.0004676	0.250586821	0.42	0.194	1	8
COX17	0.000482971	0.299971916	0.6	0.317	1	8
MRPL27	0.00050383	0.261837892	0.26	0.101	1	8
CAP1	0.000620654	0.293097026	0.76	0.512	1	8
TMEM165	0.000638287	0.250488929	0.44	0.205	1	8
NFKBIA	0.000705637	0.472810165	0.4	0.2	1	8
PRDX1	0.000729282	0.28096065	0.6	0.329	1	8
CLIC1	0.001102871	0.286309982	0.92	0.67	1	8
ATP5J	0.001228671	0.263566312	0.78	0.454	1	8
MGST3	0.001353256	0.266436576	0.42	0.2	1	8
DAZAP2	0.001531941	0.262128038	0.9	0.602	1	8

Table S2 Continued

Gene_marker	*P*_val	Avg_logFC	Pct.1	Pct.2	*P*_val_adj	Cluster
COX6A1	0.002580698	0.279757106	0.84	0.591	1	8
ABRACL	0.003088824	0.296515455	0.72	0.415	1	8
ARL6IP4	0.005859661	0.257631282	0.74	0.484	1	8
USMG5	0.005893312	0.285060757	0.82	0.57	1	8
PTGES3	0.006795499	0.265015246	0.7	0.432	1	8
IFI35	0.007688743	0.258712416	0.28	0.137	1	8
ATP5G3	0.007972003	0.255634846	0.68	0.435	1	8
CPNE5	5.14E-126	1.405203589	0.6	0.016	8.05E-122	9
CD86	4.29E-92	0.864319519	0.457	0.013	6.72E-88	9
GNG7	1.80E-88	1.191470838	0.543	0.02	2.82E-84	9
AL928768.3	7.83E-84	2.16565284	0.457	0.015	1.23E-79	9
SSPN	6.56E-77	0.670458385	0.257	0.004	1.03E-72	9
KYNU	6.68E-63	0.64783338	0.343	0.011	1.05E-58	9
MEF2C	1.47E-61	1.437322381	0.8	0.07	2.31E-57	9
PPP1R14A	1.15E-57	1.047148138	0.4	0.017	1.80E-53	9
BLK	3.31E-55	1.017677452	0.714	0.057	5.19E-51	9
FCER2	2.34E-50	0.714626556	0.286	0.01	3.67E-46	9
ZBTB32	7.21E-50	0.547690448	0.314	0.012	1.13E-45	9
ANXA4	1.55E-47	0.92045491	0.629	0.051	2.42E-43	9
KIAA0125	2.13E-47	0.902316085	0.343	0.015	3.34E-43	9
SCIMP	8.43E-43	0.722925898	0.371	0.02	1.32E-38	9
HVCN1	1.33E-41	0.853639775	0.457	0.032	2.08E-37	9
HCK	1.83E-41	0.496442489	0.286	0.012	2.87E-37	9
EAF2	2.08E-41	0.957141558	0.4	0.025	3.27E-37	9
IRF8	7.64E-41	0.78886217	0.543	0.044	1.20E-36	9
HHEX	8.35E-41	0.624520711	0.4	0.024	1.31E-36	9
KLK1	1.98E-38	0.588547725	0.286	0.013	3.11E-34	9
GAPT	1.27E-36	0.789682934	0.486	0.04	2.00E-32	9
FGD2	9.57E-36	0.489593271	0.286	0.014	1.50E-31	9
SCPEP1	3.20E-34	0.836042615	0.4	0.03	5.02E-30	9

Table S2 Continued

Gene_marker	*P*_val	Avg_logFC	Pct.1	Pct.2	*P*_val_adj	Cluster
TNFRSF13B	1.53E-33	0.793599689	0.429	0.035	2.39E-29	9
RASGRP3	1.80E-33	0.528065309	0.257	0.012	2.82E-29	9
CLECL1	8.34E-32	0.899005362	0.8	0.116	1.31E-27	9
EBF1	2.17E-30	0.57083391	0.286	0.017	3.40E-26	9
BANK1	4.76E-30	1.145762049	0.629	0.085	7.47E-26	9
CXCR5	3.42E-29	0.454981513	0.314	0.021	5.36E-25	9
LAT2	1.01E-27	0.960048803	0.571	0.075	1.58E-23	9
AIM2	2.23E-27	0.608543491	0.371	0.032	3.50E-23	9
SPIB	1.14E-26	0.920681384	0.571	0.075	1.79E-22	9
ARID3A	2.23E-26	0.674960049	0.4	0.038	3.49E-22	9
CCDC50	8.20E-26	0.872589033	0.629	0.092	1.29E-21	9
UBE2J1	4.83E-25	1.035181246	0.714	0.131	7.58E-21	9
CD79A	9.07E-25	1.377298675	0.971	0.267	1.42E-20	9
DUS2	9.93E-25	0.410940055	0.286	0.021	1.56E-20	9
CD180	6.15E-24	0.454076658	0.257	0.018	9.64E-20	9
HLA-DRA	5.53E-23	1.802287676	1	0.616	8.68E-19	9
POU2AF1	9.29E-23	0.776395057	0.4	0.044	1.46E-18	9
CYBB	1.90E-22	0.6523017	0.343	0.033	2.98E-18	9
POU2F2	2.02E-22	0.902221275	0.743	0.144	3.16E-18	9
HLA-DMB	3.08E-22	1.038590087	0.8	0.167	4.82E-18	9
SYK	1.57E-21	0.706988595	0.371	0.04	2.46E-17	9
CD74	3.43E-21	1.523332208	1	0.938	5.38E-17	9
MS4A1	7.41E-21	1.193373882	0.914	0.249	1.16E-16	9
HLA-DOB	7.83E-21	0.706885332	0.429	0.055	1.23E-16	9
SPI1	1.03E-20	0.639972395	0.371	0.042	1.62E-16	9
CD19	1.13E-20	0.965030779	0.629	0.115	1.77E-16	9
SNX2	2.91E-20	0.927191253	0.6	0.108	4.56E-16	9
SH2B2	7.69E-20	0.546019246	0.314	0.031	1.20E-15	9
CAPG	1.35E-19	0.923215012	0.6	0.112	2.11E-15	9
HLA-DPB1	2.63E-19	1.290823725	1	0.779	4.12E-15	9

Table S2 Continued

Gene_marker	*P*_val	Avg_logFC	Pct.1	Pct.2	*P*_val_adj	Cluster
CTSH	6.72E-19	0.643961283	0.514	0.08	1.05E-14	9
TLE1	7.48E-19	0.386278033	0.286	0.027	1.17E-14	9
CD79B	3.72E-18	0.993824382	0.971	0.31	5.83E-14	9
SND1	6.34E-18	0.789407235	0.514	0.088	9.94E-14	9
LY86	7.34E-18	0.901869535	0.543	0.098	1.15E-13	9
JCHAIN	8.42E-18	0.972853389	0.571	0.121	1.32E-13	9
DAPP1	1.53E-17	0.708209332	0.4	0.056	2.39E-13	9
HLA-DQB1	1.58E-17	1.135912876	0.857	0.283	2.47E-13	9
TMEM156	4.98E-17	0.87343292	0.457	0.076	7.81E-13	9
HLA-DQA2	5.42E-17	1.095393741	0.943	0.384	8.50E-13	9
GCSAM	9.04E-17	0.554889575	0.286	0.031	1.42E-12	9
PNOC	3.08E-16	0.457719245	0.286	0.032	4.82E-12	9
NCF4	1.08E-15	0.549269113	0.457	0.076	1.69E-11	9
IFT57	1.09E-15	0.629799275	0.429	0.069	1.70E-11	9
FCGR2B	1.83E-15	0.608301447	0.343	0.048	2.86E-11	9
HLA-DPA1	1.89E-15	1.180582682	0.971	0.682	2.97E-11	9
TBC1D22A	3.61E-15	0.481449916	0.371	0.053	5.66E-11	9
DAAM1	3.97E-15	0.413147432	0.286	0.033	6.23E-11	9
BTK	6.89E-15	0.584455401	0.429	0.073	1.08E-10	9
PXK	1.77E-14	0.502720181	0.314	0.042	2.78E-10	9
HLA-DQA1	3.41E-14	0.970303743	0.829	0.291	5.35E-10	9
SIGLEC10	4.43E-14	0.413606975	0.257	0.03	6.94E-10	9
HSPA4	6.09E-14	0.632973373	0.371	0.059	9.55E-10	9
HLA-DOA	8.64E-14	0.406731405	0.257	0.03	1.35E-09	9
PKIG	2.29E-13	0.407194974	0.257	0.031	3.59E-09	9
GUCD1	2.41E-13	0.609389708	0.343	0.054	3.79E-09	9
IGKC	5.80E-13	0.448295928	1	0.995	9.09E-09	9
TCF4	6.26E-13	0.815397135	0.429	0.085	9.82E-09	9
CYSLTR1	1.02E-12	0.494040947	0.371	0.062	1.60E-08	9
SUB1	1.35E-12	1.020298531	0.971	0.703	2.12E-08	9

Table S2 Continued

Gene_marker	*P*_val	Avg_logFC	Pct.1	Pct.2	*P*_val_adj	Cluster
TKT	2.00E-12	0.737284673	0.629	0.163	3.14E-08	9
ACP5	2.81E-12	0.687687046	0.571	0.144	4.40E-08	9
PLAC8	3.01E-12	1.043250572	0.657	0.197	4.72E-08	9
P2RX5	3.63E-12	0.631255548	0.4	0.075	5.69E-08	9
LAPTM5	8.15E-12	0.950190236	0.943	0.706	1.28E-07	9
HLA-DMA	1.18E-11	0.815836231	0.8	0.299	1.85E-07	9
HLA-DRB1	1.20E-11	0.89917861	1	0.656	1.88E-07	9
PAX5	1.30E-11	0.444380729	0.257	0.036	2.03E-07	9
GABARAPL2	1.66E-11	0.745667136	0.886	0.369	2.60E-07	9
PDLIM1	2.19E-11	0.558123126	0.571	0.141	3.43E-07	9
TMEM154	5.18E-11	0.610711136	0.457	0.104	8.12E-07	9
LYN	6.60E-11	0.436064232	0.4	0.08	1.03E-06	9
CYB561A3	9.86E-11	0.595869122	0.514	0.13	1.55E-06	9
SEL1L3	9.87E-11	0.521447806	0.371	0.074	1.55E-06	9
SNX3	1.17E-10	0.864487117	0.886	0.456	1.83E-06	9
MARCH1	1.19E-10	0.581421762	0.4	0.084	1.87E-06	9
DNAJC10	1.29E-10	0.434092933	0.4	0.081	2.02E-06	9
MRPS18A	1.40E-10	0.397326275	0.371	0.072	2.19E-06	9
SYNGR2	1.46E-10	0.611715231	0.686	0.204	2.28E-06	9
STX7	1.90E-10	0.71295084	0.514	0.136	2.97E-06	9
FCRLA	2.05E-10	0.682572368	0.343	0.067	3.21E-06	9
PLPP5	2.44E-10	0.515389073	0.429	0.095	3.82E-06	9
PLEKHO1	2.46E-10	0.582038532	0.314	0.058	3.86E-06	9
CD37	2.52E-10	0.731690298	1	0.727	3.96E-06	9
BCAS4	5.70E-10	0.661068887	0.457	0.11	8.94E-06	9
RPLP1	6.78E-10	0.373029247	1	1	1.06E-05	9
OAZ1	7.59E-10	0.577225947	1	0.901	1.19E-05	9
PLCG2	9.73E-10	0.467776618	0.286	0.051	1.53E-05	9
LYPLAL1	1.03E-09	0.499785578	0.257	0.042	1.62E-05	9
LINC00936	1.17E-09	0.585223287	0.4	0.091	1.83E-05	9

Table S2 Continued

Gene_marker	*P*_val	Avg_logFC	Pct.1	Pct.2	*P*_val_adj	Cluster
CD22	1.25E-09	0.507264982	0.286	0.051	1.96E-05	9
KCNN4	1.56E-09	0.374819217	0.314	0.059	2.45E-05	9
ZFAND6	1.72E-09	0.656343756	0.714	0.236	2.70E-05	9
ADK	2.37E-09	0.576045765	0.286	0.053	3.71E-05	9
CAMK1D	2.98E-09	0.291832173	0.257	0.042	4.67E-05	9
CHD7	3.25E-09	0.288558396	0.286	0.051	5.09E-05	9
FAM26F	3.33E-09	0.352383128	0.257	0.044	5.22E-05	9
CTSZ	5.19E-09	0.506075488	0.286	0.055	8.13E-05	9
SP140	6.45E-09	0.489362787	0.457	0.119	0.000101138	9
SPINT2	1.30E-08	0.631937909	0.371	0.091	0.000203091	9
DRAM2	1.30E-08	0.587165837	0.6	0.187	0.000203779	9
PPA1	1.30E-08	0.505487327	0.6	0.184	0.000203969	9
EEF2	1.73E-08	0.522022166	1	0.892	0.000271479	9
GNB2L1	2.20E-08	0.420458952	1	0.983	0.000344699	9
CD82	2.40E-08	0.59956749	0.514	0.153	0.000376282	9
ORAI2	2.42E-08	0.473406651	0.514	0.145	0.000379534	9
CDCA7L	3.33E-08	0.313310049	0.286	0.056	0.000521362	9
UVRAG	3.33E-08	0.330513654	0.257	0.048	0.000521751	9
BORCS5	3.83E-08	0.32697772	0.257	0.048	0.00060045	9
NANS	5.39E-08	0.411486327	0.257	0.051	0.000844796	9
ITGAE	9.05E-08	0.471918721	0.429	0.117	0.001417957	9
RABEP2	1.43E-07	0.470562824	0.286	0.062	0.002239069	9
SMIM14	1.75E-07	0.644983543	0.543	0.183	0.002748747	9
MTSS1	2.59E-07	0.428618623	0.286	0.064	0.00405765	9
TNFRSF13C	2.80E-07	0.378259419	0.457	0.132	0.004391649	9
MT-CO3	3.53E-07	0.410602597	1	0.994	0.005530943	9
AP1S2	3.76E-07	0.477369096	0.514	0.165	0.005901338	9
ASAH1	3.96E-07	0.421197082	0.429	0.122	0.00620383	9
CCDC167	4.08E-07	0.515412179	0.486	0.154	0.006394154	9
CALM2	4.43E-07	0.543555456	1	0.714	0.006948408	9

Table S2 Continued

Gene_marker	*P*_val	Avg_logFC	Pct.1	Pct.2	*P*_val_adj	Cluster
GRHPR	4.47E-07	0.507805889	0.4	0.115	0.007010947	9
LYL1	5.55E-07	0.314262943	0.286	0.064	0.008696652	9
UBE2N	5.77E-07	0.640167862	0.686	0.3	0.009039783	9
GPX1	6.88E-07	0.532845191	0.743	0.326	0.010781581	9
GRINA	7.62E-07	0.337222947	0.257	0.056	0.01194402	9
TFEB	8.34E-07	0.306637936	0.257	0.055	0.013080496	9
MTPN	8.72E-07	0.414364935	0.486	0.15	0.013663728	9
CMTR2	1.11E-06	0.285149969	0.286	0.066	0.017471408	9
MRPL40	1.19E-06	0.47334008	0.343	0.092	0.018731055	9
BLNK	1.43E-06	0.359736991	0.314	0.08	0.022354154	9
LSP1	1.43E-06	0.542944301	0.914	0.676	0.022369248	9
ANKRD13A	1.45E-06	0.326154928	0.314	0.078	0.022697978	9
PARP1	1.48E-06	0.526846533	0.6	0.238	0.023259653	9
MT-CO1	1.49E-06	0.33774151	1	0.998	0.023299704	9
ATP2B1	1.54E-06	0.504257472	0.4	0.124	0.024202094	9
ODC1	1.58E-06	0.340621664	0.429	0.126	0.024726348	9
TAF9	1.60E-06	0.423684559	0.457	0.143	0.025106032	9
NCR3	1.68E-06	0.321012925	0.257	0.057	0.026357496	9
DBNL	1.74E-06	0.5828485	0.657	0.255	0.027220777	9
SYPL1	1.86E-06	0.4942558	0.457	0.15	0.029215443	9
SKAP2	2.07E-06	0.320685337	0.286	0.069	0.032407802	9
PABPC4	2.14E-06	0.526814998	0.457	0.155	0.033617116	9
IDI1	2.24E-06	0.341128525	0.486	0.152	0.035125303	9
CRIP1	2.31E-06	0.937604584	0.886	0.56	0.036243566	9
SNRNP25	2.32E-06	0.328106844	0.286	0.069	0.036323748	9
OAS1	2.32E-06	0.295441171	0.286	0.069	0.036382887	9
NOB1	3.19E-06	0.302818204	0.343	0.092	0.05006747	9
CAPN1	3.41E-06	0.379538792	0.429	0.133	0.053419979	9
IRF7	3.47E-06	0.651262423	0.371	0.114	0.054443502	9
SMAGP	3.49E-06	0.302956414	0.314	0.081	0.054729607	9

Table S2 Continued

Gene_marker	*P*_val	Avg_logFC	Pct.1	Pct.2	*P*_val_adj	Cluster
CYB5R3	3.75E-06	0.365041348	0.343	0.095	0.05878333	9
SWAP70	4.16E-06	0.36451882	0.257	0.062	0.065207761	9
NAP1L1	4.31E-06	0.649465155	0.829	0.578	0.067500729	9
ARHGAP27	4.35E-06	0.296354312	0.257	0.061	0.068154169	9
LMO4	4.44E-06	0.378276219	0.4	0.124	0.069555267	9
WDR46	4.87E-06	0.286556614	0.314	0.083	0.076377016	9
CIB1	4.92E-06	0.956834571	0.886	0.461	0.077101292	9
QRSL1	5.48E-06	0.265709673	0.343	0.094	0.085879838	9
PTPN6	5.84E-06	0.838844948	0.6	0.286	0.091524893	9
CD52	5.88E-06	0.39674136	1	0.996	0.092163508	9
RPL18A	5.94E-06	0.283641324	1	1	0.093100665	9
MPDU1	6.91E-06	0.321559884	0.314	0.086	0.108292926	9
FUT8	6.99E-06	0.326522588	0.257	0.063	0.109645728	9
TPM4	8.19E-06	0.384872909	0.486	0.168	0.128326543	9
COPB1	8.88E-06	0.360264116	0.343	0.1	0.139268027	9
FAM96A	9.39E-06	0.458817457	0.429	0.144	0.147153098	9
DDAH2	9.66E-06	0.317021929	0.343	0.097	0.151484501	9
UBE2A	1.06E-05	0.383849154	0.429	0.143	0.166511895	9
CTSS	1.08E-05	0.503189994	0.686	0.33	0.168578309	9
COPS2	1.11E-05	0.328844297	0.314	0.089	0.173257212	9
GEMIN7	1.19E-05	0.342407784	0.371	0.115	0.186143095	9
CDC123	1.23E-05	0.325734826	0.371	0.115	0.193242649	9
UQCRH	1.28E-05	0.505231117	0.857	0.527	0.200096518	9
LTB	1.54E-05	1.059066414	0.8	0.56	0.241663307	9
SHMT2	1.84E-05	0.35537011	0.286	0.08	0.28804385	9
RASGRP2	1.97E-05	0.559817217	0.6	0.253	0.309531741	9
ATP6V0D1	2.00E-05	0.364238671	0.371	0.12	0.314181972	9
ANXA2	2.05E-05	0.502614403	0.629	0.274	0.321725443	9
LSM10	2.30E-05	0.388083847	0.514	0.19	0.360625336	9
RIOK3	2.33E-05	0.328769881	0.286	0.08	0.365996002	9

Table S2 Continued

Gene_marker	*P*_val	Avg_logFC	Pct.1	Pct.2	*P*_val_adj	Cluster
UPF3B	2.88E-05	0.386280117	0.314	0.093	0.4507689	9
PTK2B	2.94E-05	0.323401615	0.371	0.119	0.460560743	9
CHMP4B	3.15E-05	0.409471462	0.343	0.11	0.493184636	9
EVI2A	3.33E-05	0.46952359	0.6	0.253	0.522490387	9
SMAP2	3.52E-05	0.322908047	0.343	0.108	0.551927068	9
GRN	3.80E-05	0.444772093	0.371	0.131	0.595804276	9
CYBA	4.15E-05	0.349461349	1	0.926	0.651160258	9
PARVG	4.42E-05	0.302493098	0.4	0.133	0.692473746	9
LRMP	5.22E-05	0.269517212	0.257	0.069	0.817785168	9
RPS8	5.40E-05	0.296153203	1	0.999	0.845827097	9
RNASET2	5.81E-05	0.407580788	0.829	0.424	0.910066347	9
ADD1	5.86E-05	0.259478738	0.343	0.107	0.919184147	9
MBD4	6.33E-05	0.473508404	0.343	0.116	0.992908526	9
NSA2	6.65E-05	0.4588642	0.571	0.248	1	9
PTPN18	6.92E-05	0.39916202	0.343	0.112	1	9
PNPLA8	7.12E-05	0.384681425	0.257	0.074	1	9
HSH2D	7.38E-05	0.310771868	0.314	0.1	1	9
CXXC5	7.61E-05	0.268681249	0.257	0.073	1	9
ARPC3	8.25E-05	0.413431509	0.914	0.735	1	9
ATP5F1	8.46E-05	0.475123161	0.714	0.336	1	9
CMTM6	8.53E-05	0.354237616	0.514	0.207	1	9
RALGPS2	8.59E-05	0.370386328	0.286	0.089	1	9
TCEA1	9.01E-05	0.442410879	0.743	0.382	1	9
PLEKHF2	9.67E-05	0.275700598	0.343	0.112	1	9
PTPN1	0.000100762	0.25125528	0.343	0.112	1	9
MRPL23	0.000101579	0.345307047	0.4	0.144	1	9
NCF1	0.000103595	0.428606498	0.486	0.205	1	9
LINC00926	0.000108969	0.372264078	0.257	0.077	1	9
CSK	0.000110931	0.411702226	0.6	0.266	1	9
NUDT4	0.000111287	0.329469899	0.257	0.075	1	9

Table S2 Continued

Gene_marker	*P*_val	Avg_logFC	Pct.1	Pct.2	*P*_val_adj	Cluster
SRP72	0.000113843	0.263115567	0.429	0.153	1	9
AIDA	0.000137129	0.305013831	0.314	0.102	1	9
ATP5A1	0.000137255	0.463574046	0.686	0.363	1	9
CNDP2	0.00014802	0.250882674	0.4	0.141	1	9
TMEM219	0.000150361	0.250020112	0.543	0.212	1	9
UPF2	0.000151658	0.282171258	0.571	0.235	1	9
ATP2A3	0.000191205	0.250032569	0.343	0.117	1	9
RPL29	0.000200949	0.256494327	1	0.991	1	9
ABI1	0.000217179	0.384700885	0.514	0.226	1	9
FGR	0.000221661	0.492363759	0.457	0.188	1	9
PPP3CA	0.000235104	0.313987653	0.343	0.119	1	9
MOB1A	0.000242845	0.322845372	0.6	0.254	1	9
GRB2	0.000243702	0.379699443	0.486	0.209	1	9
NT5C	0.000244927	0.355771296	0.514	0.216	1	9
LIMD2	0.000275666	0.352364985	0.943	0.734	1	9
ST13	0.000325288	0.35453207	0.829	0.409	1	9
PPM1K	0.00034277	0.417400858	0.429	0.178	1	9
MMADHC	0.000362122	0.385240403	0.486	0.206	1	9
PRDX1	0.000368173	0.472163544	0.629	0.33	1	9
YPEL5	0.000378709	0.35279716	0.6	0.28	1	9
MT-CO2	0.000397515	0.277051552	1	0.997	1	9
EVI2B	0.000397815	0.503249439	0.771	0.444	1	9
TMEM256	0.000404331	0.34481894	0.514	0.23	1	9
LY96	0.000418417	0.37174029	0.286	0.101	1	9
NACA	0.000422361	0.310204543	0.971	0.909	1	9
STK38	0.000479426	0.308296952	0.314	0.115	1	9
PTDSS1	0.000519863	0.330221949	0.257	0.084	1	9
SLC25A6	0.000536597	0.32309577	0.971	0.84	1	9
GGA2	0.000591785	0.267502705	0.314	0.113	1	9
AP1M1	0.000596455	0.291644527	0.314	0.113	1	9

Table S2 Continued

Gene_marker	*P*_val	Avg_logFC	Pct.1	Pct.2	*P*_val_adj	Cluster
OLA1	0.000610461	0.284956572	0.286	0.099	1	9
EEF1B2	0.000626952	0.315550954	0.971	0.801	1	9
NDUFB9	0.000651294	0.367591081	0.771	0.436	1	9
HMGA1	0.000677398	0.398283579	0.371	0.15	1	9
NT5C3A	0.000693024	0.30648252	0.343	0.132	1	9
CCT8	0.000695436	0.302572563	0.543	0.241	1	9
OXA1L	0.000696924	0.273338664	0.457	0.192	1	9
GDI2	0.000724219	0.495447813	0.686	0.407	1	9
GLUL	0.000754275	0.281161358	0.286	0.101	1	9
MRPL52	0.000761893	0.283340784	0.543	0.245	1	9
JAGN1	0.000763913	0.297489909	0.257	0.088	1	9
ATP5B	0.000774385	0.396356207	0.714	0.357	1	9
EIF3D	0.000842622	0.424791954	0.686	0.395	1	9
PFDN4	0.000878498	0.265434986	0.457	0.193	1	9
ARPC1B	0.000885727	0.332566759	0.914	0.704	1	9
YWHAH	0.000894813	0.331788928	0.429	0.185	1	9
PABPC1	0.000904162	0.294197552	1	0.908	1	9
PPP1CC	0.000941138	0.357256521	0.686	0.357	1	9
DYNLL1	0.000955433	0.393826381	0.686	0.436	1	9
PTP4A1	0.000993315	0.294155976	0.314	0.119	1	9
MRPS15	0.001003921	0.262973015	0.4	0.161	1	9
ELOVL5	0.001135136	0.265330435	0.429	0.18	1	9
EPS15	0.001164698	0.303181041	0.371	0.149	1	9
CAST	0.001164953	0.283110891	0.514	0.226	1	9
SP100	0.001178403	0.37009103	0.571	0.274	1	9
IGKV3-20	0.001219153	0.273712909	0.314	0.121	1	9
CD24	0.001249085	0.466832907	0.314	0.128	1	9
SHKBP1	0.001250338	0.342708185	0.429	0.192	1	9
MYCBP2	0.00129475	0.374241793	0.371	0.156	1	9
C1QBP	0.001297641	0.314286658	0.486	0.23	1	9

Table S2 Continued

Gene_marker	*P*_val	Avg_logFC	Pct.1	Pct.2	*P*_val_adj	Cluster
ARL5A	0.001400323	0.26753719	0.6	0.297	1	9
ZFAS1	0.001419005	0.377073999	0.714	0.423	1	9
MT-ND1	0.001584912	0.26237101	0.943	0.796	1	9
UBXN1	0.001611274	0.324231049	0.771	0.46	1	9
UCP2	0.001655313	0.410382476	0.771	0.531	1	9
S1PR4	0.001656997	0.359625657	0.6	0.311	1	9
LMBRD1	0.001684844	0.302088673	0.257	0.095	1	9
POLD4	0.001891638	0.277885956	0.457	0.206	1	9
MTMR14	0.001991676	0.347697075	0.257	0.1	1	9
ECHS1	0.002017975	0.308322175	0.371	0.158	1	9
ITSN2	0.002088547	0.31635367	0.4	0.176	1	9
UBL7	0.002091011	0.266403202	0.286	0.11	1	9
EZR	0.002193001	0.438420606	0.6	0.32	1	9
TMEM134	0.002470961	0.351707131	0.371	0.165	1	9
PPP2R1A	0.002488377	0.357072408	0.429	0.209	1	9
PGLS	0.002674473	0.369315834	0.4	0.186	1	9
HOPX	0.002752195	0.281635785	0.514	0.231	1	9
UBE2D2	0.002763638	0.334363598	0.743	0.429	1	9
CAPZB	0.002770322	0.348105551	0.771	0.556	1	9
GLRX	0.003147253	0.308488687	0.429	0.205	1	9
HN1	0.003177719	0.345250049	0.514	0.253	1	9
UBE2I	0.003205503	0.360183663	0.657	0.352	1	9
CLIC1	0.003358083	0.382630247	0.829	0.672	1	9
CLNS1A	0.003383791	0.287776461	0.457	0.218	1	9
PLP2	0.003504081	0.384717513	0.457	0.233	1	9
ATP5L	0.003876584	0.251768127	1	0.851	1	9
RGS2	0.004002401	0.477240672	0.257	0.107	1	9
IDS	0.004062923	0.268944261	0.514	0.267	1	9
STX8	0.004092212	0.254318951	0.314	0.132	1	9
HSPD1	0.004229722	0.292909317	0.343	0.149	1	9

Table S2 Continued

Gene_marker	*P*_val	Avg_logFC	Pct.1	Pct.2	*P*_val_adj	Cluster
PSMC5	0.004315253	0.268182863	0.543	0.292	1	9
ARPC2	0.004435013	0.255957416	0.943	0.803	1	9
ERH	0.004777474	0.278130389	0.686	0.384	1	9
RP5-1171I10.5	0.00523856	0.445836656	0.571	0.303	1	9
TESC	0.005374488	0.252794672	0.257	0.101	1	9
SCAND1	0.005683619	0.272362578	0.657	0.336	1	9
ARPC5	0.005760634	0.347180054	0.829	0.519	1	9
TES	0.005817062	0.254175392	0.343	0.152	1	9
PYCARD	0.005922218	0.26710986	0.543	0.297	1	9
SLC25A5	0.005932609	0.279862432	0.657	0.34	1	9
GHITM	0.006164743	0.272170323	0.657	0.377	1	9
CD53	0.006184047	0.459291812	0.743	0.61	1	9
GAPDH	0.006199705	0.288076658	0.971	0.881	1	9
ILK	0.006410951	0.268222281	0.314	0.138	1	9
RGS19	0.006463511	0.292180379	0.457	0.233	1	9
ISG20	0.007184007	0.25850704	0.8	0.54	1	9
FCMR	0.007617984	0.272318768	0.486	0.251	1	9
ARPC5L	0.008773498	0.285817638	0.543	0.314	1	9
TBCB	0.009010597	0.345431957	0.6	0.331	1	9
CCT7	0.009011124	0.262447728	0.371	0.178	1	9
MTDH	0.009768061	0.331051471	0.6	0.396	1	9

Table S3 Types of cell subpopulations

Cluster	Cell type	Gene
0	Type I effector CD8+ memory T (Tem) cell	CD2/CD27/CD3D/CD3E/CD3G/CD8A/PTPRC
1	Type II effector CD8+ memory T (Tem) cell	FGFBP2/GZMB/GZMH/KLRD1
2	Naive CD8+ T cell	LDHB/RPS8
3	Type I B cell (CCND1+ CD79A+ TNFRSF13C+)	CD19/CD24/CD37/CD74/CD79A/CD79B/MS4A1
4	Type II B cell (CCND1+ CD79A+)	CD74/CD79A/CD79B/MS4A1
5	Natural killer cell	GNLY/GZMB/KLRB1/KLRC1/KLRD1/KLRF1/NKG7
6	Plasmacytoid dendritic cell	CREB3L2/DERL3/FKBP2/GAS6/GNG7/HERPUD1/HSP90B1/ITM2C/LMAN1/MZB1/NPC2/NUCB2/PDIA4/SEC11C/SEL1L3/SPCS1/SSR4/TXNDC5/UBE2J1
7	Type III B cell	CD74/MS4A1
8	CD1C-CD141- dendritic cell	ABI3/AIF1/APOBEC3A/ARRB2/ASAH1/ATP1B3/BCL2A1/BID/C5AR1/CAMK1/CASP1/CD300E/CD68/CDC42EP3/CDKN1C/CFD/CLEC12A/CLEC7A/CPPED1/CSF1R/CTSS/CXCL16/DOK2/DRAP1/DUSP1/DUSP6/FCGR2A/FCGR3A/FCN1/FGR/FTH1/FTL/GBP2/GPBAR1/HCK/HMOX1/HSBP1/IFI30/IFITM2/IFITM3/ITM2B/LGALS3/LILRA2/LILRA5/LILRB1/LILRB2/LRRC25/LST1/LTA4H/LYN/MS4A4A/MS4A7/MT2A/NAMPT/NAP1L1/NCF2/OAS1/PECAM1/PILRA/POU2F2/PSAP/PTPN6/RAB24/RALB/RHOC/RNF149/S100A11/S100A4/SAT1/SDCBP/SERPINA1/SIGLEC10/SLC11A1/SLC2A6/SLC31A2/SLC7A7/SOD2/STX11/STXBP2/TBXAS1/TCF7L2/TCIRG1/TESC/TIMP1/TKT/TMEM176B/TNFRSF1B/TNFSF10/TUBA1A/TYMP/TYROBP/VAMP5/VASP/VMP1/WARS/WSB1/ZEB2/ZFAND5
9	Type IV B cell (CD79A+)	CD19/CD37/CD74/CD79A/CD79B/MS4A1

Table S4 Routine blood index values

Item	Result	Abnormal	Units	Reference range
κ light chain (kappa)	6.75	High	g/L	1.7–3.7
λ light chain (lambda)	3.49	High	g/L	0.9–2.1
Immunoglobulin G (lgG)	25.6	High	g/L	7.2–16.8
Immunoglobulin M (lgM)	0.48	Low	g/L	0.6–2.8
Immunoglobulin E (lgE)	10900	High	kIU/L	0–100
White blood cell/globulin ratio (A/G)	1	Low		1.5–2.5
Indirect bilirubin (|Bi|)	3.7	Low	μmol/L	5.1–13.7
Globulin (Glo)	39.4	High	g/L	20.0–30.0
Neutrophil percentage (Neut%)	33.4	Low	%	40–75
Percentage of lymphocytes (lymph%)	60.9	High	%	20–50
Red blood cell count (RBC)	3.14	Low	T/L	4.3–5.8
Hemoglobin (Hb)	93	Low	g/L	130–175
Hematocrit (Hct)	0.28	Low	L/L	0.40–0.50
Platelet count (PLT)	97	Low	G/L	125–350
Platelet specific product (PCT)	0.1	Low	ml/L	0.11–0.28
Platelet volume distribution width (PDW)	11	Low	GSD	15.2–17.4

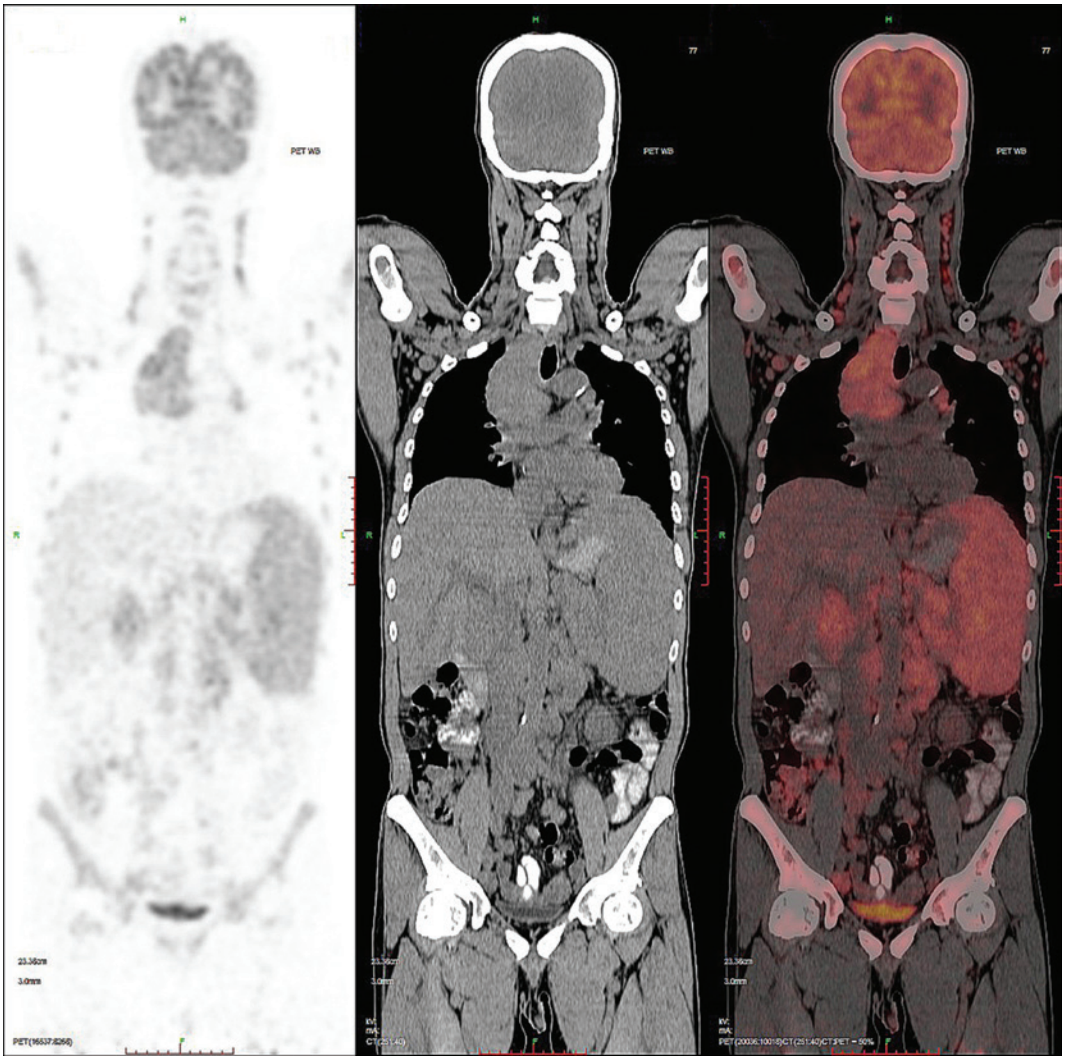

Figure S1 PET-CT of the patient in this study.

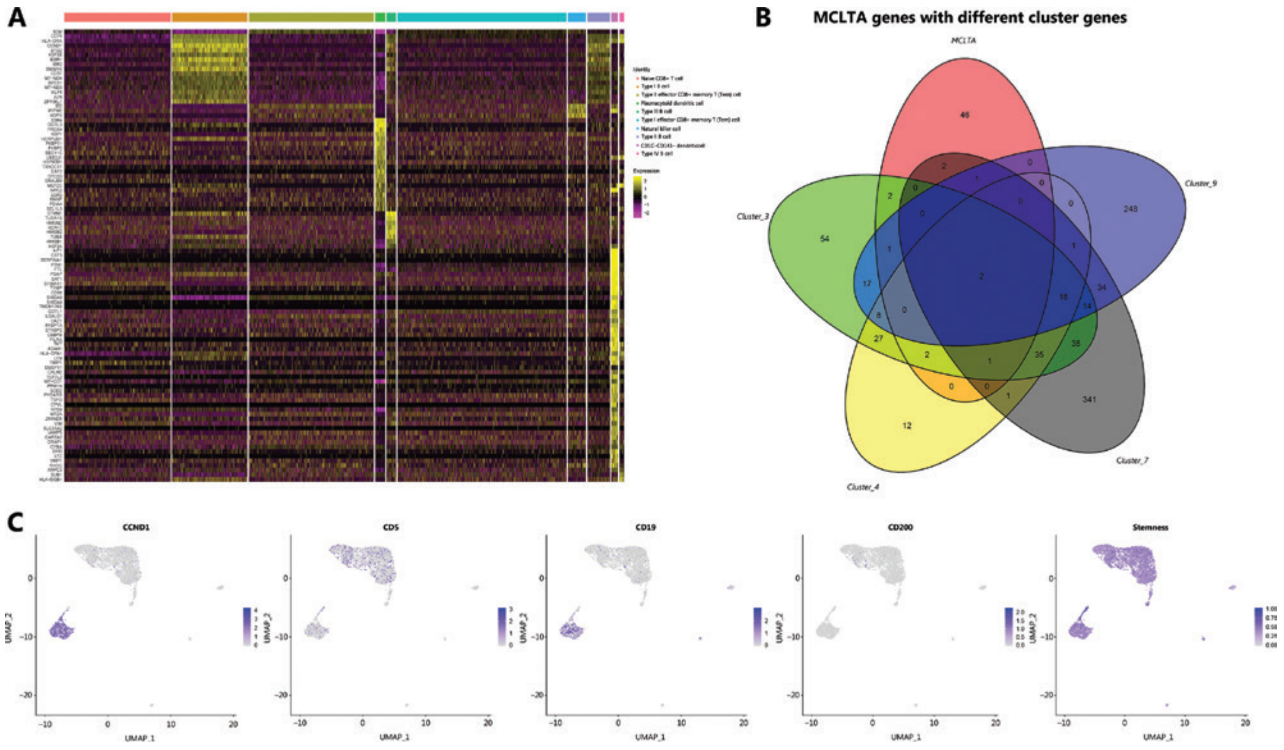

Figure S2 Identification of malignant B cell subpopulations. (A) Gene expression heat map of stem cell related genes. (B) Venn diagram. Common genes between the MCL tumor antigen and malignant B cell clusters. (C) Cell map of malignant B cell clusters showing specific markers.

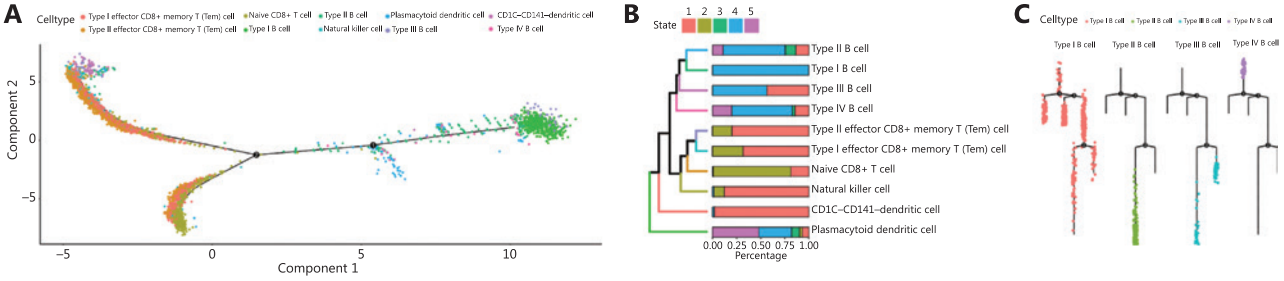

Figure S3 Pseudotime analysis of cell subpopulations in the bone marrow of a patient with mantle cell lymphoma. (A) The state distribution of different cell clusters in MCL. Each point corresponds to a single cell, and each color represents a cell type. (B) Cell cluster hierarchical clustering. Similarity ranked from high to low. (C) Pseudotime analysis of type I-IV malignant B cells.

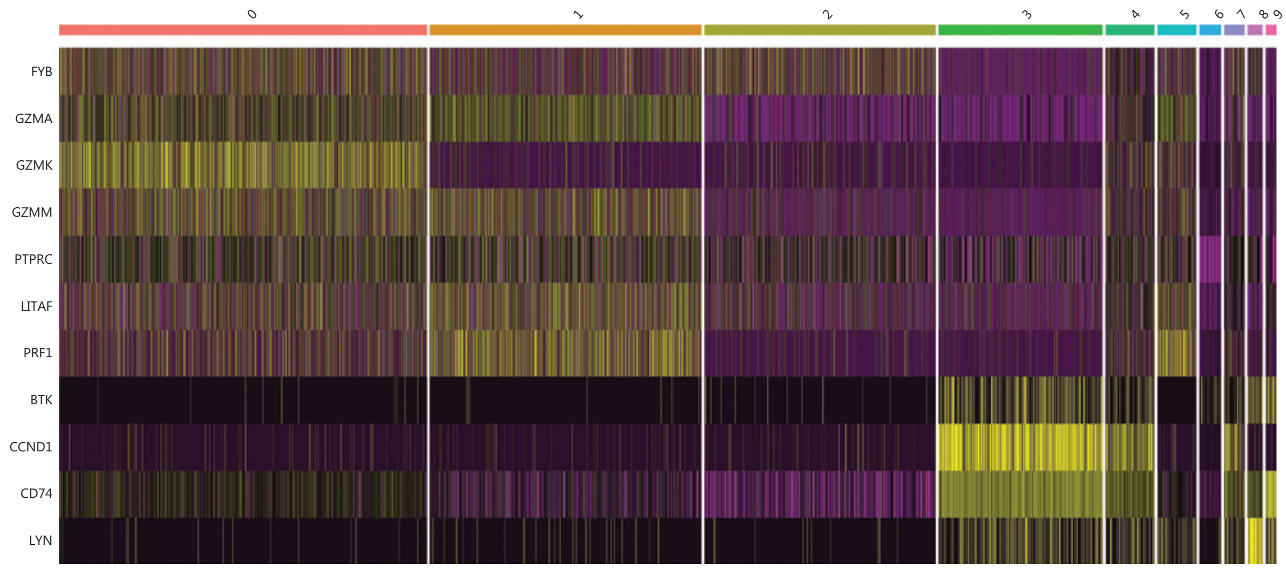

Figure S4 Expression heatmap of genes involved in immune mechanisms. Each row represents a gene, and each column represents a single cell.

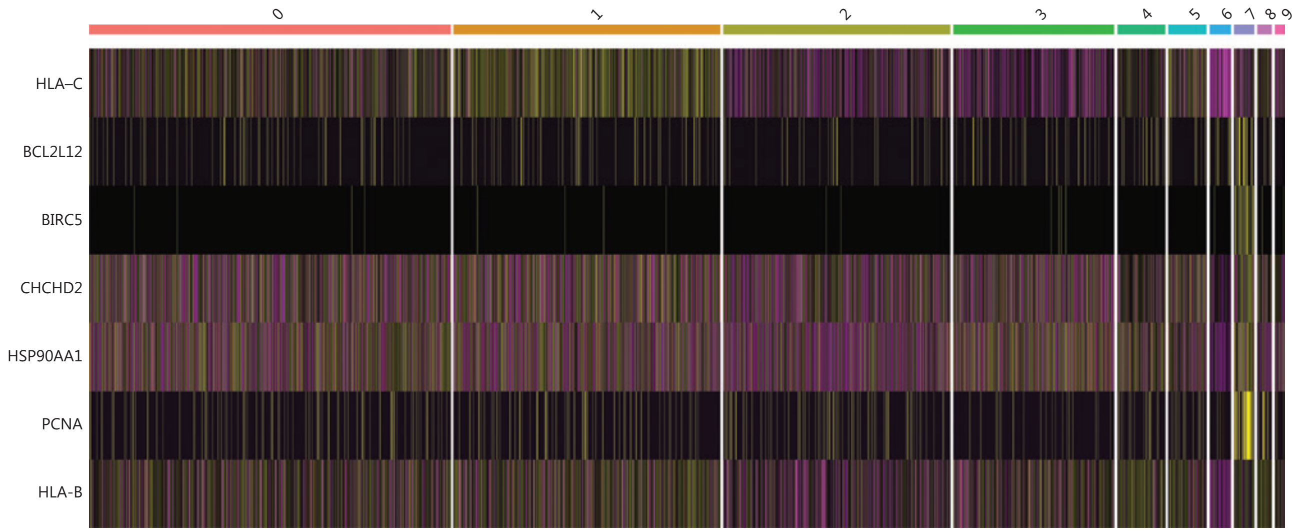

Figure S5 Expression heatmap of genes involved in the immune escape mechanism. Each row represents a gene, and each column represents a single cell.

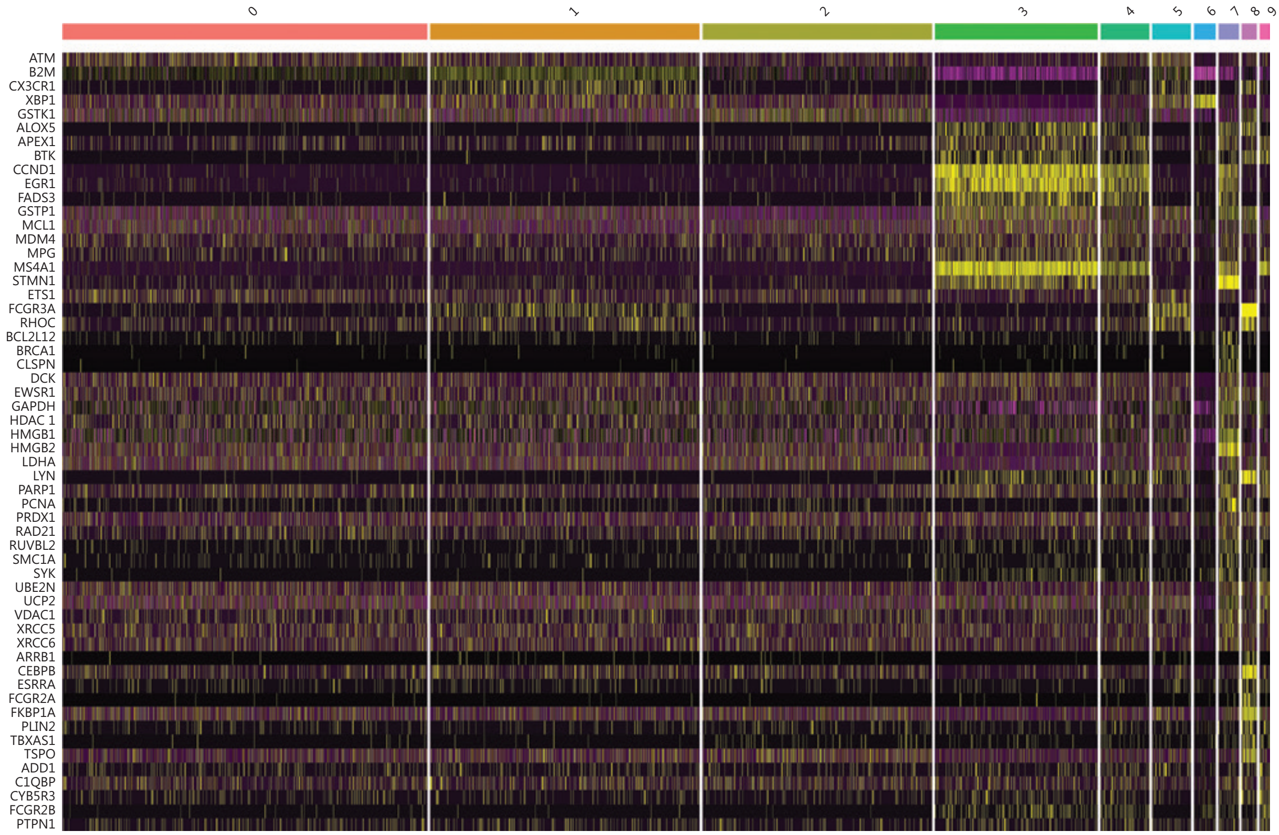

Figure S6 Expression heatmap of genes involved in drug resistance. Each row represents a gene, and each column represents a single cell.

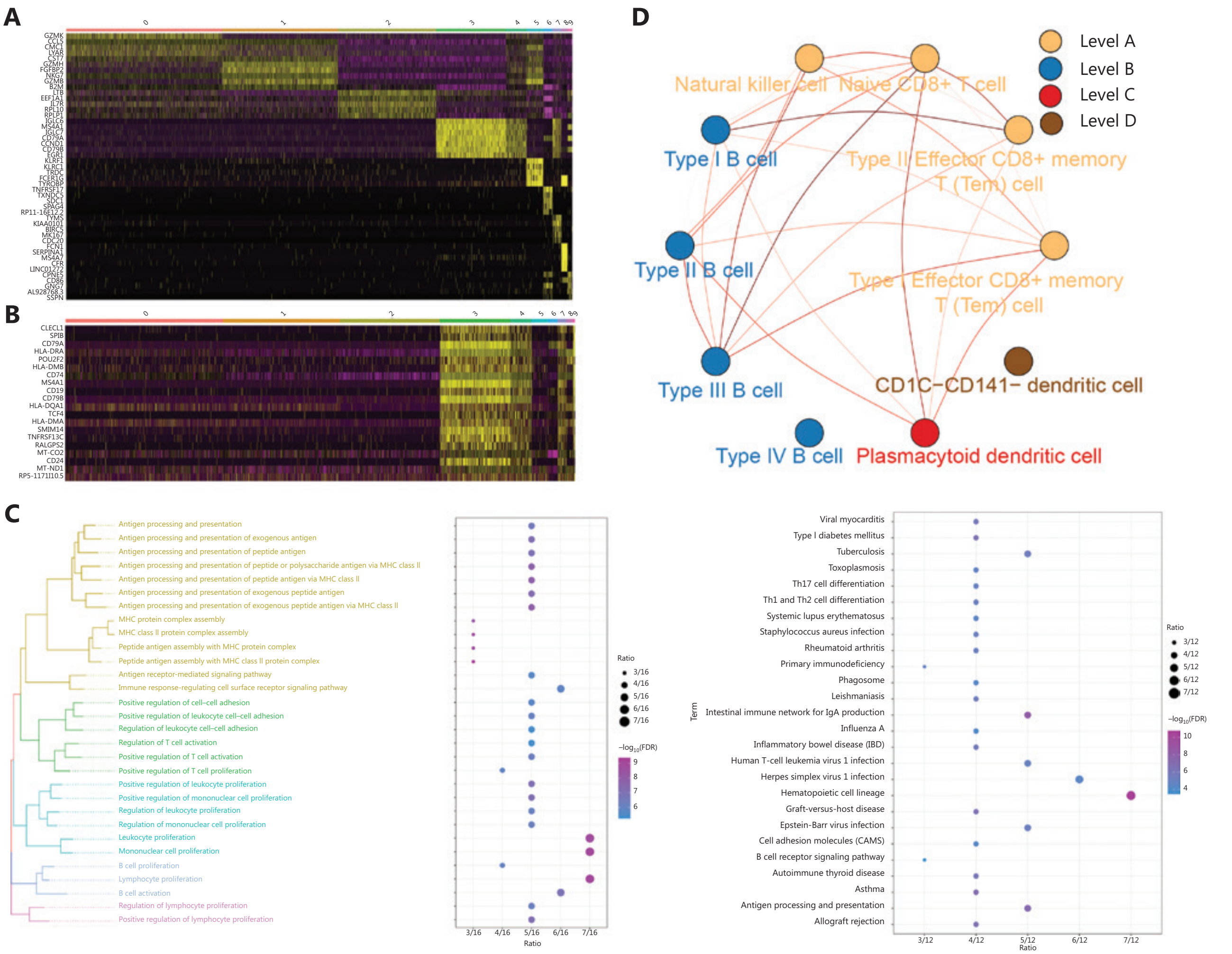

Figure S7 Functional enrichment analysis of differentially expressed genes in malignant B cells and correlation analysis between cell subpopulations. (A) Expression heatmap of differentially expressed genes for each cell subpopulation. Each row represents a gene, and each column represents a single cell. (B) Expression heatmap of genes abnormally expressed in all malignant cells. Each row represents a gene, and each column represents a single cell. (C) Functional enrichment analysis of differentially expressed genes in four malignant B cells. The significance of enrichment gradually increased from blue to purple, and the size of the dots indicates the number of differentially expressed genes in the corresponding pathways. (D) The correlation of cell clusters. The connections indicate that different cell clusters are related to each other.

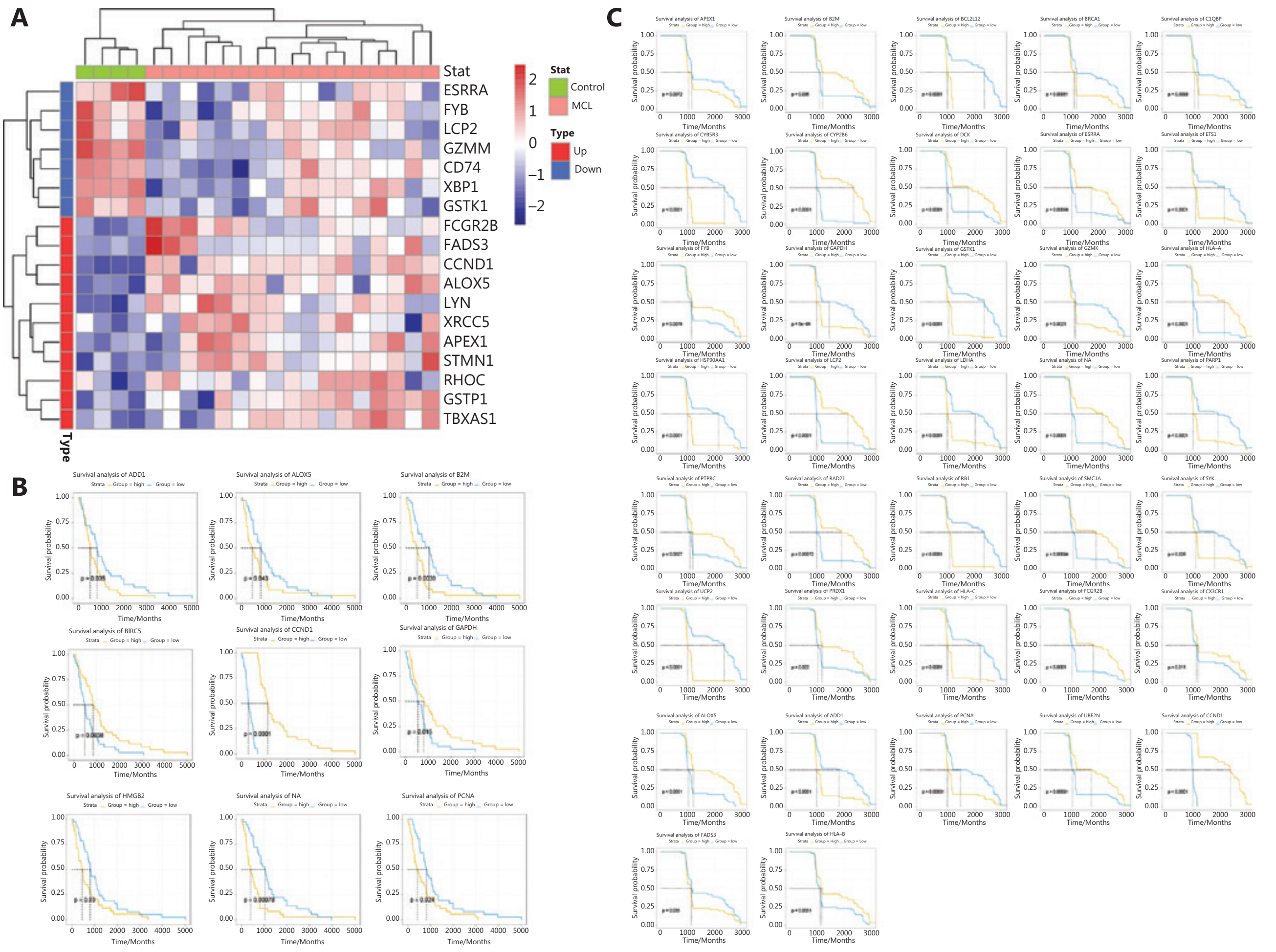

Figure S8 The conclusions regarding expression imbalance and clinical prognostic potential for key marker genes were verified in an independent clinical cohort of patients with MCL. (A) Heatmap of common genes between mechanism genes and differentially expressed genes. (B) Prognostic potential of the key marker genes in a clinical cohort of patients with MCL in the GSE10793 dataset. (C) Prognostic potential of the key marker genes in a clinical cohort of patients with MCLs in the GSE93291 dataset.
